# Psychiatric and neurological predictors of early ADHD medication discontinuation across the lifespan: a multinational study

**DOI:** 10.1136/bmjment-2025-302469

**Published:** 2026-07-02

**Authors:** Isabell Brikell, Aske Astrup, Theresa Wimberley, Masako Araki, Zheng Chang, Ditte Demontis, Zihan Dong, Stephen V Faraone, Le Gao, Malcolm B Gillies, Jan Haavik, Catharina Hartman, Henrik Larsson, Kenneth K C Man, Sallie-Anne Pearson, Harold Snieder, Melissa Vos, Ian C K Wong, Honghui Yao, Andrew SC Yuen, Yanli Zhang-James, Yiling Zhou, Helga Zoega, Anders Engeland, Søren Dalsgaard, Kari Klungsøyr

**Affiliations:** 1 Department of Medical Epidemiology and Biostatistics, Karolinska Institute, Stockholm, Stockholm County, Sweden; 2 Department of Global Public Health and Primary Care, University of Bergen, Bergen, Hordaland, Norway; 3 National Centre for Register-Based Research, Aarhus University, Aarhus, Central Denmark Region, Denmark; 4 Centre for Integrated Register-based Research, Aarhus University, Aarhus, Central Denmark Region, Denmark; 5 National Drug and Alcohol Research Centre, University of New South Wales, Sydney, New South Wales, Australia; 6 Department of Biomedicine—Human Genetics, Aarhus University, Aarhus, Central Denmark Region, Denmark; 7 Center for Genomics and Personalized Medicine, Aarhus University, Aarhus, Denmark; 8 Departments of Psychiatry and of Neuroscience and Physiology, SUNY Upstate Medical University, Syracuse, New York, USA; 9 Department of Pharmacy Administration, School of Pharmacy, Xi’an Jiaotong University, Xi’an, Shaanxi, China; 10 Department of Pharmacology and Pharmacy, Li Ka Shing Faculty of Medicine, The University of Hong Kong, Hong Kong, Hong Kong; 11 School of Population Health, Faculty of Medicine and Health, University of New South Wales, Sydney, New South Wales, Australia; 12 Department of Biomedicine, University of Bergen, Bergen, Hordaland, Norway; 13 Division of Psychiatry, Haukeland University Hospital, Bergen, Hordaland, Norway; 14 Interdisciplinary Center Psychopathology and Emotion Regulation (ICPE), Department of Psychiatry, University of Groningen, University Medical Center Groningen, Groningen, Netherlands; 15 School of Medical Sciences, Örebro University, Orebro, Sweden; 16 Research Department of Practice and Policy, School of Pharmacy, University College London, London, UK; 17 Centre for Medicines Optimisation Research and Education, University College London Hospitals NHS Foundation Trust, London, England, UK; 18 Department of Epidemiology, University of Groningen, University Medical Center Groningen, Groningen, Netherlands; 19 Aston Pharmacy School, Aston University, Birmingham, England, UK; 20 Department of Pharmacology and Pharmacy, University of Hong Kong Faculty of Medicine, Hong Kong, Hong Kong; 21 Department of Psychiatry and Behavioral Sciences, SUNY Upstate Medical University, Syracuse, New York, USA; 22 University Medical Centre Groningen, Groningen, GR, Netherlands; 23 Department of Epidemiology, University of Groningen, Groningen, GR, Netherlands; 24 Centre of Public Health Sciences, Faculty of Medicine, University of Iceland, Reykjavík, Capital Region, Iceland; 25 Department of Chronic Diseases, Norwegian Institute of Public Health, Bergen, Norway; 26 Child and Adolescent Mental Health Center, Copenhagen University Hospital Rigshospitalet, Copenhagen, Capital Region of Denmark, Denmark; 27 Department of Clinical Medicine, University of Copenhagen, Copenhagen, Capital Region of Denmark, Denmark; 28 Department of Health Promotion, Norwegian Institute of Public Health, Bergen, Norway, Norway

**Keywords:** Attention Deficit and Disruptive Behavior Disorders, Neurodevelopmental Disorders, Psychiatry, Psychopharmacology

## Abstract

**Background:**

Early discontinuation of attention-deficit/hyperactivity disorder (ADHD) medication is common and linked to worse outcomes. Identifying clinical predictors could aid personalised treatment yet evidence is inconsistent across ages and countries/regions.

**Objective:**

Investigate psychiatric and neurological comorbidity as predictors of early ADHD medication discontinuation in new ADHD medication users across age groups, sex and countries/regions.

**Methods:**

Using health records from eight countries/regions, we identified 1 000 411 (44% female) new ADHD medication users (2011–2020). Discontinuation was defined as a ≥180 day gap between dispensations. We examined 23 indicators of psychiatric or neurological comorbidity, severity and psychotropic medication use. Associations were estimated using Cox regression, pooled with random-effects meta-analyses and stratified by age-at-initiation and sex.

**Findings:**

Discontinuation rates varied widely (children 19%–61%, adolescents 37%–68%, young adults 52–67%, adults 38%–68%). In pooled analyses, earlier discontinuation in children was predicted by intellectual disability, autism and use of psychotropic medications (HR range 1.32–1.51), while conduct/oppositional defiant disorder (CD/ODD) was protective (HR 0.83, 95% CI 0.73 to 0.94). In adolescents, no indicators remained statistically significant after multiple-testing control. In young adults, CD/ODD (HR 1.42, 95% CI 1.30 to 1.55), and in adults, schizophrenia (HR 1.25, 95% CI 1.09 to 1.44) and tic disorders (HR 1.27, 95% CI 1.11 to 1.46) predicted earlier discontinuation. Statistical heterogeneity was substantial, largely driven by US estimates. In meta-analyses excluding the USA, additional associations emerged. For example, in children, OCD and anxiety disorders predicted earlier discontinuation, while eating disorders and antidepressants/anxiolytics were protective in adults. Associations with schizophrenia, tic disorders and CD/ODD were no longer significant. Country-specific analyses showed similar association patterns, except in the USA, Hong Kong and the UK. Sex differences were limited.

**Conclusions:**

Children with neuropsychiatric comorbidity and related comedication are more likely to discontinue ADHD medication early, whereas few consistent predictors were seen from adolescence onwards. Marked cross-country variation, particularly in the USA, points to system-level influences on treatment patterns.

**Clinical implications:**

Improving ADHD medication persistence will require consideration of healthcare context and age-specific strategies, including close monitoring for children with complex neuropsychiatric profiles, and consideration of broader factors in adolescents and adults, where clinical predictors were limited.

**WHAT IS ALREADY KNOWN ON THIS TOPIC**

- Psychiatric comorbidity has been linked to attention-deficit/hyperactivity disorder (ADHD) medication discontinuation, but effects vary across specific conditions and age groups. Few studies have considered regional differences, and no previous studies have examined clinical predictors across multiple countries, age groups and sex using consistent measures.

**WHAT THIS STUDY ADDS**

- We analysed health records from over one million new ADHD medication users across eight countries/regions and found generally high discontinuation rates, varying across age groups (lower rates in children) and countries (higher rates in the USA).
- Children showed the most clinical predictors of discontinuation, including autism, intellectual disability and other psychotropic medication use, whereas few predictors emerged from adolescence onwards.
- Patterns were broadly consistent across countries except for Hong Kong, the UK and the USA, while sex differences were minimal across age groups and countries.

**HOW THIS STUDY MIGHT AFFECT RESEARCH, PRACTICE OR POLICY**

- Neuropsychiatric comorbidity and related treatments are important predictors of discontinuation in children, supporting the need for enhanced monitoring.
- The limited predictive value of psychiatric and neurological indicators in older groups highlights the need to study other drivers, while cross-country differences point to the influence of healthcare systems on treatment persistence.

·

## Background

Attention-deficit/hyperactivity disorder (ADHD) is a neurodevelopmental condition affecting 5%–8% of children and adolescents, and 2%–5% of adults.^1 2^ ADHD medications are central to treatment, with high efficacy in treating core ADHD symptoms and related impairment, although response and tolerability vary.^3 4^ ADHD medication treatment is also linked to reduced risks of substance misuse, accidents, self-harm and educational failure.^5^,^7^ Conversely, meta-analyses have linked non-adherence to increased risk of adverse outcomes in youth.^8^ Despite this, 50%–80% discontinue ADHD medication within 1–2 years, with the highest rates seen in adolescence and young adulthood.^9^,^12^ A recent multinational study found that 65% of children, 47% of adolescents, 39% of young adults and 48% of adults remained on treatment after 1 year.^13^ These discontinuation rates exceed expected symptom remission within a similar timeframe,^14^ suggesting that many patients stop treatment prematurely. Identifying predictors of discontinuation that can be measured at treatment initiation could support treatment continuity and inform clinical decision-making.

Psychiatric and neurological comorbidity is common in ADHD and can influence treatment choice, efficacy and tolerability, which in turn impacts ADHD medication discontinuation.^9^,^12^ Psychiatric comorbidity has generally been linked to poorer treatment persistence, but evidence on the predictive value of specific conditions, their medical treatment and severity remain mixed and may differ by age.^9^,^12^ In children with ADHD, conduct disorder and oppositional defiant disorder (CD/ODD) have been associated with worse adherence,^10^ whereas findings for intellectual disability, anxiety and autism are mixed.^10 15 16^ Fewer studies have focused on adults, but comorbid substance use disorder (SUD) has been linked to lower adherence, efficacy and more side effects.^16^,^18^ ADHD medication treatment in individuals with symptoms or history of mania, psychosis or compulsive behaviours may be impacted by concerns of symptom exacerbation, yet few studies have examined associations with discontinuation.^9^,^1216^ Concomitant psychotropic medication use has been associated with improved adherence in children in some studies,^11^ but its role in discontinuation in adolescents and adults, where polypharmacy is more common, is poorly understood. Beyond psychiatric comorbidity, ADHD is associated with a range of neurological conditions including headaches, migraine, sleep disorders and epilepsy.^4 19^ ADHD medications have been found to both improve and exacerbate symptoms of these conditions,^19^ yet their impact on discontinuation remains largely unexplored.

### Objective

Synthesis of existing evidence is hindered by heterogeneity in the definition and measurement of predictors and discontinuation, and the lack of studies considering national, regional and health system differences.^9^,^11^ To address this, we used healthcare data from eight countries/regions, harmonised definitions and a multinational cohort design to evaluate the extent to which psychiatric and neurological indicators predict early discontinuation of ADHD medication across countries/regions, age and sex.

## Methods

### Data sources

In this multinational cohort study, we used pseudo-anonymised registers data or electronic health records from eight countries/regions to obtain data on diagnoses and ADHD medication dispensations or prescriptions. In Denmark, the Netherlands, Norway and Sweden, we used national registers capturing specialist care diagnoses and pharmacy dispensations. In Hong Kong SAR, we used electronic health records from public primary and specialist care with territory-wide coverage.^20^ In Australia, data came from a linked administrative database covering pharmacy dispensations and hospital diagnoses for all residents aged ≥18 years in New South Wales.^20^ In the UK, we used primary care records of diagnoses and prescriptions covering ~6% of the population.^21^ In the USA, we used a federated electronic health records dataset (TriNetX Research Network) including primary and specialist diagnoses and medication dispensations, covering ~1.2 million individuals with ADHD.^22^ Henceforth, the term dispensation is used to describe both prescriptions (from the UK) and dispensations. Medication data were available from 1 January 2009 to 31 December 2020 in most countries/regions, except Australia (from 1 July 2012) and Norway (until 31 December 2019). Dispensations were recorded by Anatomical Therapeutic Chemical (ATC) classification codes or mapped source-specific equivalents. Clinical diagnoses were generally available for the same period and recorded using the International Classification of Diseases (ICD) 10th revision or mapped source-specific equivalents. In the Netherlands, diagnoses were available for youth <18 years from 2011 to 2014, and for adults ≥18 from 2011 to 2020. Ethnicity data were unavailable. Healthcare setting, population coverage and medication reimbursement policies for the different countries/regions are summarised in online supplemental table S1.

### Study population

We used a multinational cohort design including new ADHD medication users, defined as individuals with a dispensation of ADHD medication between 1 January 2011 and 31 December 2020, following a 2-year washout period without any dispensation. Data permitting, we excluded individuals with a record of emigration or death prior to initiation (as these likely reflect errors), those initiating before age 4, when ADHD medication use is rare, and after age 60, due to variable coverage and potential off-label use in older populations. Country-specific exclusions are in online supplemental table S2.

### ADHD medication discontinuation

Our primary outcome was *early ADHD medication discontinuation*. In line with prior research, discontinuation was defined as a gap of ≥180 days between dispensations.^13 23^ In four out of the eight contributing countries/regions, the maximum length of a single prescription is 90 days (ie, Netherlands, Norway, Sweden and USA). We selected two times the maximum prescription length (ie, 180 days) without a dispensing, to ensure we captured clinically meaningful medication breaks, rather than shorter medication gaps. Initiation was set to the date of the first dispensation following the washout period and the date of discontinuation to the last dispensation date plus 180 days.^13^ To qualify as early discontinuation, the last dispensation had to occur within 365 days of initiation, although the calculated discontinuation date could fall beyond this period (ie, within 1.5 years of initiation). Switching between ADHD medications was not considered discontinuation if the subsequent dispensation occurred within 180 days. Only the first treatment episode during follow-up was considered. We included the six most commonly used ADHD medications, which were approved during the study period in at least two of the participating sites: methylphenidate (ATC code N06BA04), amphetamine (N06BA01), dexamphetamine (N06BA02), lisdexamphetamine (N06BA12), atomoxetine (N06BA09) and guanfacine (C02AC02). While additional ADHD medications are available mainly in the USA, the included medication classes account for over 90% of USA prescriptions during the study period.^24^ Therefore, the impact of not considering such agents on our estimates is likely modest.

### Psychiatric and neurological predictors

We selected potential psychiatric and neurological predictors based on evidence of association with ADHD treatment outcomes, relevance to ADHD and data availability.^49^,^12 16 19^ We included 14 psychiatric and 3 neurological conditions, 3 indicators of psychiatric severity (multimorbidity, inpatient admission, suicide attempt/serious self-harm) and 3 indicators of psychotropic medication use: antidepressant/anxiolytic use (as proxy for depression and anxiety treated in primary care), antipsychotic use (capturing off-label use in ADHD or comorbid conditions not captured by diagnosis) and psychotropic polypharmacy. All indicators were binary and defined from diagnoses and/or dispensations within 2 years prior to ADHD medication initiation, except for polypharmacy, which was defined within 3 months prior to initiation (online supplemental table S3).

### Statistical analysis

We used a distributed network approach to perform equivalent data management and analyses at each participating site, using a common study protocol and scripts (preregistered at https://osf.io/q6eah). Associations were estimated in each country/region, and results sent to the lead analytic site (Norway) for pooled analyses and visualisation. Analyses were conducted using R (V.4.4.3).

Follow-up began at ADHD medication initiation and ended at discontinuation, death, emigration or 1.5 years after initiation, whichever came first. We used Cox proportional hazards models to estimate the association of each indicator with discontinuation separately, within each country/region, using days since initiation as the underlying time-scale. All models were stratified by age at initiation, grouped into children (4–11 years), adolescents (12–17), young adults (18–24) and adults (25–60). Within each stratum, we further adjusted for sex, calendar year and continuous age at initiation, with the latter two modelled using natural cubic splines with four degrees of freedom. We only fitted models for exposures with at least five individuals in each discontinuation status group to facilitate interpretability and convergence. This threshold could be modified to comply with local data protection regulations. Associations are reported as HRs with 95% CIs.

### Meta-analyses

Country/region-specific estimates were pooled using inverse-variance-weighted random-effects meta-analysis, with between-study variance (τ²) estimated by Restricted Maximum Likelihood. Heterogeneity in meta-analyses was assessed with Cochran’s Q and quantified by I², with values >75%, indicating substantial heterogeneity. We also performed secondary meta-analyses, excluding the US data. This was based on high heterogeneity in primary meta-analyses, divergence in estimates relative to other countries, the large US sample size and considerable differences in healthcare system in the USA compared with other sites. As our main analyses, we applied the Benjamini–Yekutieli procedure to estimates from the meta-analyses to control the false discovery rate at 5% within each age-at-initiation group. We focus in-text reporting on estimates that remained statistically significant after multiple-testing control, with complete results provided in figures. Meta-analyses were conducted in R using ‘metafor’ package.

### Country/region specific analyses

From each country/region, we report single-exposure models as the primary analyses, with the aim of exploring differences across sites and identifying a broad set of risk markers. As secondary analyses, we also ran multivariable Cox models within each country/region, using the same strata and covariate adjustment as the single-exposure models. Multivariable analyses were included to explore whether any indicators act as independent predictors when considered jointly in the same model. To reduce multicollinearity in multivariable models, we retained the two broader ICD-10 categories of ‘neurotic, stress-related and somatoform disorders’ and ‘personality disorders’ and excluded subdiagnoses within these (anxiety disorder, obsessive-compulsive disorder, borderline personality disorder). For the country/region-specific models, we report Hazard ratios (HRs) and 95% confidence intervals (CIs) without multiple-testing control to enable comparison across settings and avoid inference based solely on arbitrary significance thresholds, consistent with current statistical reasoning.^25^,^27^

### Sensitivity analyses

We conducted three sensitivity analyses. First, we reran the main analyses (ie the country/region specific single-exposure models and meta-analyses of these) stratified by sex. Second, we repeated the main analyses, including only individuals who filled a second dispensation within 180 days of initiation, with follow-up beginning at the second dispensation. This was done to assess potential bias from including individuals who never started sustained treatment. Third, we redefined indicators in each country/region using dispensations and ICD-10 coded diagnoses at any time preceding initiation (‘lifetime’), going as far back as possible in each country. This was done to assess whether longer ascertainment periods influenced estimates. Denmark did not contribute to sensitivity analyses due to data extraction restrictions.

### Role of the funding source

The funders of the study had no role in study design, data collection, analysis, interpretation or writing of the report.

### Lived experience involvement

Analyses plans and preliminary results were discussed with representatives from ADDISS (The National Attention Deficit Disorder Information and Support Service), a UK-based ADHD charity, and presented at a patient event hosted by the TIMESPAN consortium (Management of chronic cardiometabolic disease and treatment discontinuity in adults with ADHD), ADDISS and ADHD Europe.

## Management of chronic cardiometabolic disease and treatment discontinuity in adults with ADHD

collaborator and presented at a patient event hosted by the TIMESPAN consortium, ADDISS and ADHD Europe.

## Findings

We included 1 000 411 individuals initiating ADHD medication between 2011 and 2020 across eight countries/regions (44% female). In countries with good coverage across age groups (Denmark, Norway, Sweden, USA), the median age at initiation ranged from 17 to 21 years. In primarily child/adolescent samples (Hong Kong; Netherlands <18, UK), the median was 9–11 years, and 32 years in adult-only samples (Australia; Netherlands ≥18) (table 1). Methylphenidate was the most commonly prescribed medication at initiation across countries/regions (>80% of dispensations), except in the USA and Australia where methylphenidate and amphetamine derivatives were prescribed to a similar extent (online supplementaltable S4). Number of person-years, follow-up time and descriptives of discontinuation are in online supplemental table S5. Across countries/regions, discontinuation was lowest in children (19%–61%) and higher in adolescents (37%–68%), young adults (52%–67%) and adults (38%–68%). Discontinuation was highest in the USA and Australia, and lowest in Denmark (for children and adolescents) and Sweden (for young adults and adults). Online supplemental figures S1-S8 show the proportion of individuals with each clinical indicator, by discontinuation status. Prevalence generally increased with age, except for intellectual disability, autism, CD/ODD, tic disorders and epilepsy, which were more common in children.

**Table 1:** Descriptive statistics of the study population across countries/regions

Country/region	N total	ADHD diagnosis (n, %)	Sex, females (n, %)	Children 4–11 years (n, %)	Adolescents 12–17 years (n, %)	Young adults 18–24 years (n, %)	Adults 25–60 years (n, %)	Age at first dispensing in years (mean, IQR)
Australia^*^	30 295	1337 (4.41)	17 312 (57.14)	na	na	8038 (26.53)	22 257 (73.47)	32 (25, 41)
Denmark	78 677	40 666 (51.69)	32 023 (40.70)	16 673 (21.19)	14 356 (18.25)	16 530 (21.01)	31 118 (39.55)	21 (13, 32)
Hong Kong	28 954	21 590 (74.57)	6532 (22.56)	24 048 (83.06)	3488 (12.05)	490 (1.69)	928 (3.21)	9 (8,11)
Netherlands<18**^†^**	93 057	52 449 (56.36)	27 866 (29.95)	59 719 (64.17)	33 338 (35.83)	na	na	10 (8,14)
Netherlands≥18**^†^**	183 637	95 134 (51.81)	87 165 (47.47)	na	na	54 038 (29.43)	129 599 (70.57)	32 (24, 42)
Norway	53 365	47 811 (89.59)	21 652 (40.57)	18 024 (33.77)	10 386 (19.46)	7439 (13.94)	17 516 (32.82)	17 (10, 29)
Sweden	194 591	171 431 (88.10)	81 994 (42.14)	51 412 (26.42)	52 985 (27.23)	25 813 (13.27)	64 381 (33.09)	17 (12, 29)
UK	12 515	10 021 (80.07)	2916 (23.30)	6932 (55.39)	2985 (23.85)	867 (6.93)	1731 (13.83)	11 (8, 16)
USA	352 320	254 165 (78.13)	166 846 (51.29)	90 084 (27.69)	56 393 (17.33)	39 556 (12.16)	139 287 (42.82)	21 (11, 36)

* Linked data in Australia was only available for individuals ≥ 18 years.

† In the Netherlands, linked data on children/adolescents < 18 years were available from 2011 to 2014, and data on individuals above ≥ 18 years from 2011 to 2020. Numbers are therefore shown separately.

ADHD, attention-deficit/hyperactivity disorder; na, not available.

### Meta-analyses

**Figure 1:**
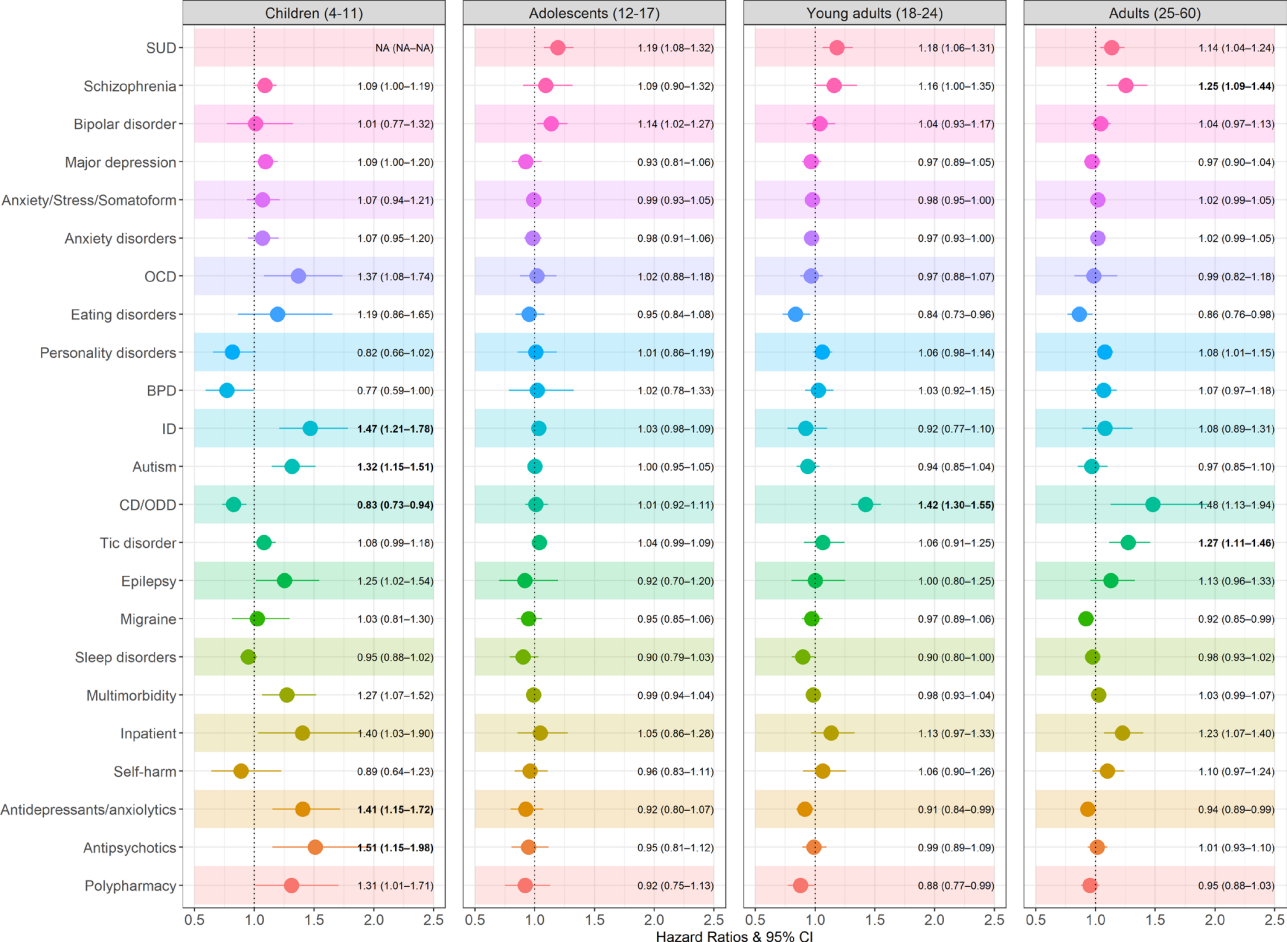
Meta-analytic associations of psychiatric and neurological indicators with ADHD medication discontinuation. HR and 95% CI show the association of each indicator with ADHD medication discontinuation. Estimates surviving false discovery rate correction are shown in bold. All indicators were defined in the two years prior to ADHD medication initiation, except for psychotropic polypharmacy that was defined within the 3 months prior to initiation. The number of contributing countries and samples size for each meta-analysis varied by clinical indicator and age groups, depending on prevalence and data availability. Forest plots showing the contributing countries, HRs, standard error, relative weight and measures of heterogeneity are provided in the supplement. ADHD, attention-deficit/hyperactivity disorder; BPD, borderline personality disorder; CD/ODD, conduct disorder/oppositional defiant disorder; ID, intellectual disability; Inpatient, psychiatric inpatient admission; OCD, obsessive compulsive disorder; Polypharmacy, psychotropic polypharmacy; SUD, substance use disorder.

Pooled results from meta-analyses including all sites are in figure 1. Among children (4–11 years), 11 of 22 indicators were associated with ADHD medication discontinuation. SUD was not assessed due to too few cases. Intellectual disability (HR 1.47, 95% CI 1.21 to 1.78), autism (HR 1.32, 95% CI 1.15 to 1.51), CD/ODD (HR 0.83, 95% CI 0.73 to 0.94) and antidepressant/anxiolytic (HR 1.41, 95% CI 1.15 to 1.72) and antipsychotic (HR 1.51, 95% CI 1.15 to 1.98) use remained statistically significant after multiple-testing control.

Among adolescents (12–17 years), only two of 23 indicators were associated with discontinuation. After multiple-testing control, no associations remained.

Among young adults (18–24 years), seven of 23 indicators were associated with discontinuation, but only the association with CD/ODD remained after multiple-testing control (HR 1.42, 95% CI 1.30 to 1.55).

Among adults (25–60 years), 9 of 23 indicators were associated with discontinuation. Associations with schizophrenia (HR 1.25, 95% CI 1.09 to 1.44) and tic disorders (HR 1.27, 95% CI 1.11 to 1.46) remained after multiple-testing control.

The number of countries/regions contributing to each meta-analysis ranged from 2 to 8, depending on indicator prevalence and data availability. Among children, 9 of 22 meta-analyses showed substantial heterogeneity (I²>75%), often driven by weaker or reversed associations in Hong Kong, the UK and the USA. In adolescents, young adults and adults, substantial heterogeneity was the norm, mainly reflecting divergent estimates in the USA compared with other countries. Forest plots with country-specific estimates, pooled results, I², τ² and Q-test p values are in online supplemental figures S9-S31.

In meta-analyses excluding US data (online supplemental figure S32), results were broadly consistent with the main meta-analyses, with some notable differences after multiple-testing control. Among children, intellectual disability, autism and antidepressant/anxiolytic showed similar strength of associations as in main meta-analyses. In addition, OCD, anxiety disorders and psychiatric multimorbidity predicted earlier discontinuation. While estimates for antipsychotic medication use and CD/ODD were similar to the main analyses, they were no longer statistically significant after multiple-testing control. Among adolescents, no association remained after multiple-testing control, consistent with the main meta-analyses. Among young adults, SUD associated with earlier discontinuation, and protective effects emerged for eating disorders, sleep disorders, antidepressants/anxiolytics and psychotropic polypharmacy. The association with CD/ODD was no longer statistically significant. Among adults, anxiety disorders and psychiatric inpatient admission associated with earlier discontinuation, while eating disorder, migraine and antidepressant/anxiolytic use showed protective effects. Associations with schizophrenia and tic disorders attenuated and were no longer statistically significant.

### Country/region specific associations

**Figure 2:**
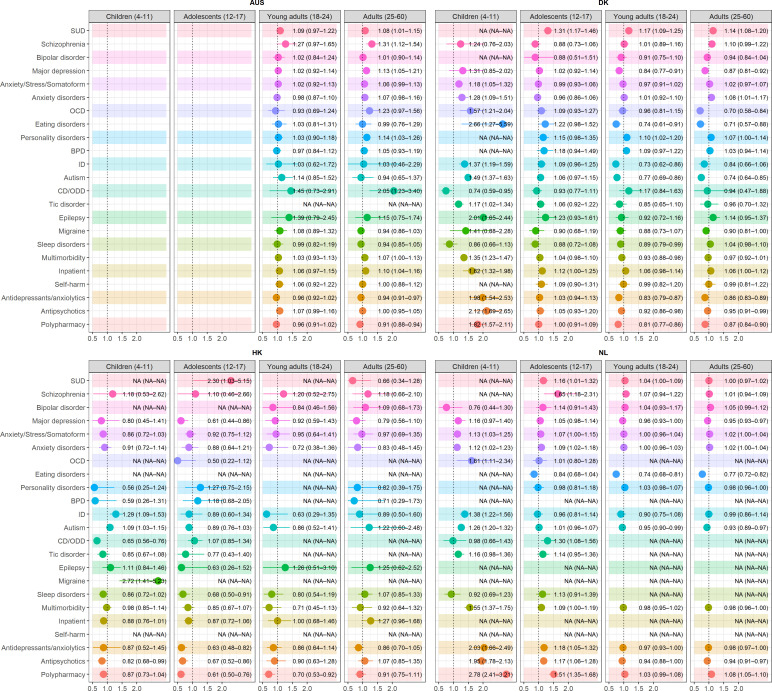
Country-specific associations of psychiatric and neurological indicators with ADHD medication discontinuation in Australia (AS), Denmark (DK), Hong Kong (HK) and the Netherlands (NL). All indicators were defined in the two years prior to ADHD medication initiation, except for psychotropic polypharmacy that was defined within the 3 months prior to initiation. ADHD, attention-deficit/hyperactivity disorder; BPD, borderline personality disorder; CD/ODD, conduct disorder/oppositional defiant disorder; ID, intellectual disability; Inpatient, psychiatric inpatient admission; OCD, obsessive compulsive disorder; Polypharmacy, psychotropic polypharmacy; SUD, substance use disorder.

Country/region-specific associations from single-exposure models are in figures2  3. In children, country/region-specific results were similar to pooled estimates from meta-analyses excluding the USA. Effects estimates tended to be larger in Denmark, Norway and Sweden, as compared with the Netherlands, Hong Kong, UK and the USA. Furthermore, while neither the primary or secondary (excluding USA) meta-analyses showed a significant association with neurotic, stress-related and somatoform disorders, these conditions were linked to higher discontinuation in all countries (HR range 1.13–1.35) except Hong Kong and the USA. Neurological conditions were generally not associated with discontinuation, except for epilepsy in Denmark, Sweden and the USA (HR range 1.26–2.01), migraine in Hong Kong and the USA (HR range 1.18–2.72) and sleep disorders in Norway (HR 1.38, 95% CI 1.06 to 1.79).

**Figure 3:**
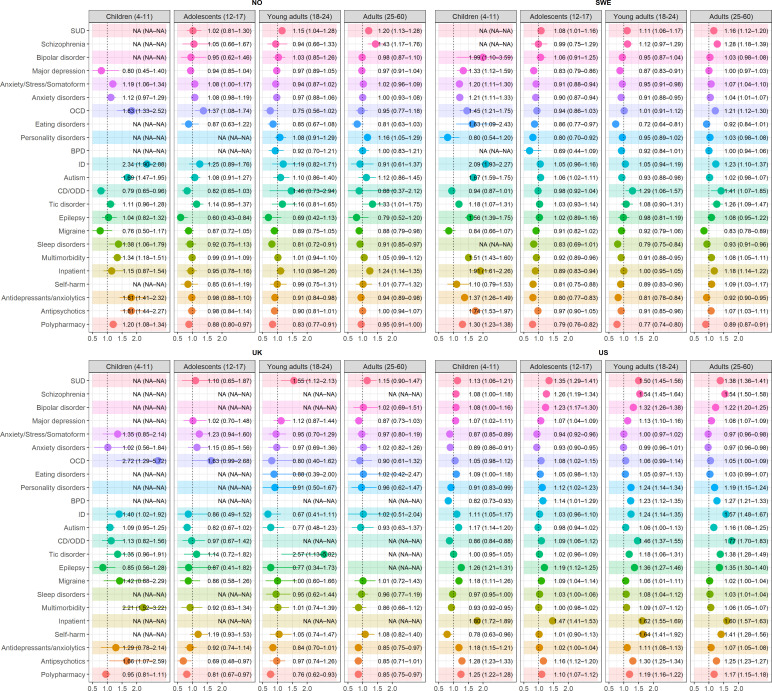
Country-specific associations of psychiatric and neurological indicators with ADHD medication discontinuation in Norway (NO), Sweden (SE), the United Kingdom (UK) and the United States (US). All indicators were defined in the 2 years prior to ADHD medication initiation, except for psychotropic polypharmacy that was defined within the 3 months prior to initiation. ADHD, attention-deficit/hyperactivity disorder; BPD, borderline personality disorder; CD/ODD, conduct disorder/oppositional defiant disorder; ID, intellectual disability; Inpatient, psychiatric inpatient admission; OCD, obsessive compulsive disorder; Polypharmacy, psychotropic polypharmacy; SUD, substance use disorder.

Country/region-specific associations in adolescents, young adults and adults (figures2  3) generally aligned with the main pooled estimates, except in the USA, were several indicators not associated in pooled analyses—and rarely in other countries—showed broader risk increasing associations. Bipolar disorder, major depression, personality disorders as a class and borderline personality disorder specifically, epilepsy and psychiatric inpatient admission were linked to higher discontinuation in all three age-at-initiation groups. Autism, intellectual disability and suicide attempt/self-harm were associated in young adults and adults only. Furthermore, all three indicators of psychotropic use were linked to higher discontinuation across ages in the USA, in contrast to most other countries where associations were null or protective from adolescence onwards. Additional protective effects, for example, for eating disorders, were observed in several other countries among young adults and adults, as reflected in the pooled analyses excluding the USA.

Results from multivariable analyses (online supplemental figures S33-S34) in each country/region were broadly consistent with the single-exposure models, although most estimates attenuated across age-groups. In children, attenuation was stronger for antidepressant/anxiolytic and antipsychotic use, while associations for autism and intellectual disability persisted. In the USA, most associations in adolescents, young adults and adults also attenuated, except for SUD and psychiatric inpatient admission, which remained associated with discontinuation.

### Sensitivity analyses

Sex-stratified pooled estimates showed minimal differences (online supplemental figure S35), consistent with country/region-specific estimates (online supplemental figures S36-S42). Across countries/regions, between 64% (in the USA) and 93% (<18-year olds in the Netherlands) of ADHD medication users refilled their initial dispensation within 180 days (online supplemental table S6). Pooled estimates in this subpopulation, with follow-up starting at the second dispensation, are in online supplemental figure S43 and country-specific estimates in online supplemental figures S44-S45. In children, estimates were directionally consistent with main analyses but attenuated, particularly for psychotropic medication use indicators. In adolescents, young adults and adults, results largely mirrored the main findings, although associations were generally stronger in the USA. Sensitivity analyses defining indicators as lifetime yielded results consistent with the main findings (online supplemental figures S46–S48).

## Discussion

Using healthcare data from eight countries/regions and harmonised definitions, we found that psychiatric and neurological comorbidities, related co-medications and indicators of higher clinical complexity predict earlier ADHD medication discontinuation in children but had less effect in adolescents and adults. Associations varied minimally by sex.

In pooled analyses including all sites, 11 of the included indicators predicted discontinuation in children. After multiple-testing control, autism, intellectual disability and use of antidepressant/anxiolytic and antipsychotic medication remained associated with earlier discontinuation. In contrast, CD/ODD showed a protective effect on discontinuation. Fewer predictors emerged for discontinuation in adolescence and adulthood. This was particularly striking in adolescents, where no associations remained after multiple-testing control. Similarly, only the associations of earlier discontinuation with CD/ODD remained in young adults, and with schizophrenia and tic disorders in adults.

Meta-analyses showed marked heterogeneity, largely attributable to the USA. Associations were fewer and weaker in US children, while several indicators not associated in other countries, predicted earlier discontinuation in US adults. The USA also showed higher rates of comorbidity and discontinuation, and proportion of patients not refilling the first dispensation (36%). We, therefore, performed meta-analyses excluding the USA. In these analyses, several additional indicators were associated with earlier discontinuation in children, and additional protective effects emerged in adults, after multiple-testing control. These pooled results more closely reflected the association patterns observed in countries other than the USA.

Specifically, among children, associations with OCD, anxiety disorders and psychiatric multimorbidity emerged as additional predictors of earlier discontinuation, while CD/ODD was no longer significantly associated with lower discontinuation. Prior research has reported mixed findings for autism and anxiety, while studies on the impact of co-occurring intellectual disability on ADHD medication discontinuation are scarce.^10 11^ The association with OCD is to our knowledge novel and may reflect the frequent co-occurrence of childhood-onset OCD with other neurodevelopmental conditions,^28^ which were consistently found to increase discontinuation. Earlier discontinuation linked to antidepressants/anxiolytics and antipsychotics may reflect their use in treating co-occurring autism, OCD and anxiety, or possible drug interactions. Assocations may also index greater clinical severity, as these medications are used more cautiously in children than in older age groups. Our findings, which represent the largest investigation to date, suggest that complex neurodevelopmental comorbidities and related treatments are associated with early ADHD medication discontinuation in children. This underscores the need for improved monitoring and adapted treatment strategies in children with ADHD and these comorbidities.

In adolescence, the absence of associated indicators persisted across meta-analyses, suggesting that early discontinuation in this age group is largely independent of the broad set of clinical indicators investigated here. As discontinuation rates peak during late adolescence^9 13^ and are linked to worse outcomes,^8^ identifying other drivers and interventions to promote sustained treatment in this age group remains a priority.

In young adults, SUD predicted earlier discontinuation after exclusion of US data, while eating disorders, sleep disorders and indicators of psychotropic medication use emerged as protective. In adults, earlier discontinuation was associated with anxiety disorders and psychiatric inpatient care, while eating disorders, migraine and antidepressant/anxiolytic use showed protective effects. Comorbid SUD has previously been linked to higher rates of discontinuation in ADHD,^16 17^ potentially reflecting dosing needs, as individuals with a history of SUD often require higher stimulant doses, and under-dosing is linked to poorer adherence.^18^ Clinicians may also discontinue stimulants in cases of relapse, misuse or diversion. Lower rates of discontinuation in persons with ADHD and co-occurring eating disorders have not been reported before. The effect may relate to perceived benefits of stimulant-related appetite suppression, alongside use of stimulants in certain eating disorders, including lisdexamfetamine for binge-eating disorder.^29^ While anxiety diagnoses predicted earlier discontinuation, antidepressants/anxiolytics use predicted later discontinuation. These opposing effects may relate to severity, as anxiety diagnoses come from specialist psychiatric care in the majority of the included countries/regions, while antidepressant/anxiolytic dispensation also captures individuals treated in primary care. It may also reflect reported positive effects of ADHD medication on depression in adults.^7^

Finally, after excluding the USA, several associations in the main meta-analyses were no longer statistically significant, including the risk-increasing effects of CD/ODD in young adults and of schizophrenia and tic disorders in adults. These diagnoses are relatively rare, and the large US sample likely had greater power to detect effects not observed elsewhere. Indeed, several indicators not significant at other sites or in pooled analyses (eg, bipolar disorder, major depression, personality disorders, autism, psychotropic medications use) predicted higher discontinuation in US adults. However, the diverging estimates found in the US analyses likely also reflects systematic differences in data coverage, clinical practice and healthcare systems. Unlike the other contributing sites, which have universal healthcare and near-complete register follow-up, the US TriNetX data aggregate electronic health records from hundreds of providers serving both insured and uninsured patients.^22^ Fragmented coverage and follow-up can inflate discontinuation estimates. US studies also show that disparities in insurance coverage and costs affect ADHD treatment and psychiatric care more broadly.^30 31^ These structural differences likely contribute to the more widespread associations between clinical complexity and earlier discontinuation observed in US adults.

### Strengths and limitations

This study has notable strengths, including the use of real-world data from eight countries/regions, harmonised definitions and coverage from childhood to mid-to-late adulthood, enabling age-specific and sex-specific analyses. There are also several limitations, including under-representation of low-income and middle-income countries, where patterns may differ, and lack of harmonised data on socioeconomic status and treatment variables (eg, prescriber, dosage, care setting) that may influence discontinuation and confound associations. We excluded individuals over age 60 due to limited coverage; as recognition of ADHD in older adults grows, treatment patterns in this group warrant further study. Finally, we lacked data on actual dispensation length and reasons for discontinuation. As such, associations may differ in studies with more precise discontinuation measures, or in analyses accounting for discontinuation cause.

### Implications and conclusion

Children with neuropsychiatric comorbidity, related co-medication and higher clinical severity discontinued ADHD medication earlier, underscoring the need for tailored support, monitoring and alternative treatment strategies. In adolescents and adults, fewer psychiatric and neurological indicators predicted discontinuation, indicating that other factors may be more important. Beyond response and tolerability, future studies should assess factors such as patient engagement, service continuity and structural barriers. The distinct US patterns emphasise the role of healthcare context and suggest that clinical complexity in adults may have greater consequences in systems without universal coverage. Improving treatment persistence, and the ability to identify those at risk of premature discontinuation, will likely require attention to both individual clinical factors and the broader organisational and social conditions shaping ADHD care.

## Supplementary material

10.1136/bmjment-2025-302469 online supplemental file 1

## Data Availability

Data may be obtained from a third party and are not publicly available.
