# Supplementary material for: Psychiatric and neurological predictors of early ADHD medication discontinuation across the lifespan: a multinational study
Source: BMJ Ment Health. 2026 Jul 2;29(1):e302469. doi: 10.1136/bmjment-2025-302469 (PMC13330961; doi:10.1136/bmjment-2025-302469)
Supplement: online supplemental file 1 [file bmjment-29-1-s001.docx]

# **Supplementary materials - Psychiatric and neurological indicators of early ADHD medication discontinuation across the lifespan**

[Table S1. Summary of healthcare setting, population coverage, and medication reimbursement policies for the different countries/region 5](#_Toc230891095)

[Table S2. Exclusion criteria and numbers retained at each step, by country/region 7](#_Toc230891096)

[Table S3. Definition of clinical characteristics 8](#_Toc230891097)

[Table S4. ADHD medication at initiation across countries/regions 9](#_Toc230891098)

[Table S5. Follow-up time and medication discontinuation summary by country and age-group 10](#_Toc230891099)

[Table S6. Descriptive statistics of individuals with a second ADHD medication dispensation within 180 days of initiation 12](#_Toc230891100)

[Table S7. Ethical approval and data sharing statement by country 14](#_Toc230891101)

[Figure S1. Percent with psychiatric and neurological indicators by discontinuation status at end of follow-up in Australia 16](#_Toc230891102)

[Figure S2. Percent with psychiatric and neurological indicators by discontinuation status at end of follow-up in Denmark 17](#_Toc230891103)

[Figure S3. Percent with psychiatric and neurological indicators by discontinuation status at end of follow-up in Hong Kong 18](#_Toc230891104)

[Figure S4. Percent with psychiatric and neurological indicators by discontinuation status at end of follow-up in the Netherlands 19](#_Toc230891105)

[Figure S5. Percent with psychiatric and neurological indicators by discontinuation status at end of follow-up in Norway 20](#_Toc230891106)

[Figure S6. Percent with psychiatric and neurological indicators by discontinuation status at end of follow-up in Sweden 21](#_Toc230891107)

[Figure S7. Percent with psychiatric and neurological indicators by discontinuation status at end of follow-up in the UK 22](#_Toc230891108)

[Figure S8. Percent with psychiatric and neurological indicators by discontinuation status at end of follow-up in US 23](#_Toc230891109)

[Figure S9: Forest plots for Children – Schizophrenia spectrum disorders; Children – Bipolar disorder; Children –Major depression; Children – Neurotic, stress related, and somatoform disorders 24](#_Toc230891110)

[Figure S10: Forest plots for Children – Anxiety disorders; Children – Obsessive compulsive disorder; Children – Eating disorders; Children – Personality disorders 25](#_Toc230891111)

[Figure S11: Forest plots for Children – Borderline personality disorder; Children – Intellectual disability; Children – Autism spectrum disorder; Children – Conduct disorders 26](#_Toc230891112)

[Figure S12: Forest plots for Children – Tic disorder; Children – Epilepsy; Children – Migraine; Children – Sleep disorders 27](#_Toc230891113)

[Figure S13: Forest plots for Children – Psychiatric multimorbidity; Children – Psychiatric inpatient hospitalization; Children – Suicide attempt/intentional self harm; Children – Antidepressants anxiolytics 28](#_Toc230891114)

[Figure S14: Forest plots for Children – Antipsychotics; Children – Psychotropic medication; Adolescents – Substance use disorders; Adolescents – Schizophrenia spectrum disorders 29](#_Toc230891115)

[Figure S15: Forest plots for Adolescents – Bipolar disorder; Adolescents – Major depression; Adolescents – Neurotic, stress related, and somatoform disorders; Adolescents – Anxiety disorders 30](#_Toc230891116)

[Figure S16: Forest plots for Adolescents – Obsessive compulsive disorder; Adolescents – Eating disorders; Adolescents – Personality disorders; Adolescents – Borderline personality disorder 31](#_Toc230891117)

[Figure S17: Forest plots for Adolescents – Intellectual disability; Adolescents – Autism spectrum disorder; Adolescents – Conduct disorders; Adolescents – Tic disorder 32](#_Toc230891118)

[Figure S18: Forest plots for Adolescents – Epilepsy; Adolescents – Migraine; Adolescents – Sleep disorders; Adolescents – Psychiatric multimorbidity 33](#_Toc230891119)

[Figure S19: Forest plots for Adolescents – Psychiatric inpatient hospitalization; Adolescents – Suicide attempt intentional self harm; Adolescents – Antidepressants anxiolytics; Adolescents – Antipsychotics 34](#_Toc230891120)

[Figure S20: Forest plots for Adolescents – Psychotropic medication; Young adults – Substance use disorders; Young adults – Schizophrenia spectrum disorders; Young adults – Bipolar disorder 35](#_Toc230891121)

[Figure S21: Forest plots for Young adults – Major depression; Young adults – Neurotic, stress related, and somatoform disorders; Young adults – Anxiety disorders; Young adults – Obsessive compulsive disorder 36](#_Toc230891122)

[Figure S22: Forest plots for Young adults – Eating disorders; Young adults – Personality disorders; Young adults – Borderline personality disorder; Young adults – Intellectual disability 37](#_Toc230891123)

[Figure S23: Forest plots for Young adults – Autism spectrum disorder; Young adults – Conduct disorders; Young adults – Tic disorder; Young adults – Epilepsy 38](#_Toc230891124)

[Figure S24: Forest plots for Young adults – Migraine; Young adults – Sleep disorders; Young adults – Psychiatric multimorbidity; Young adults – Psychiatric inpatient hospitalization 39](#_Toc230891125)

[Figure S25: Forest plots for Young adults – Suicide attempt intentional self harm; Young adults – Antidepressants anxiolytics; Young adults – Antipsychotics; Young adults – Psychotropic medication 40](#_Toc230891126)

[Figure S26: Forest plots for Adults – Substance use disorders; Adults – Schizophrenia spectrum disorders; Adults – Bipolar disorder; Adults – Major depression 41](#_Toc230891127)

[Figure S27: Forest plots for Adults – Neurotic, stress related, and somatoform disorders; Adults – Anxiety disorders; Adults – Obsessive compulsive disorder; Adults – Eating disorders 42](#_Toc230891128)

[Figure S28: Forest plots for Adults – Personality disorders; Adults – Borderline personality disorder; Adults – Intellectual disability; Adults – Autism spectrum disorder 43](#_Toc230891129)

[Figure S29: Forest plots for Adults – Conduct disorders; Adults – Tic disorder; Adults – Epilepsy; Adults – Migraine 44](#_Toc230891130)

[Figure S30: Forest plots for Adults – Sleep disorders; Adults – Psychiatric multimorbidity; Adults – Psychiatric inpatient hospitalisation; Adults – Suicide attempt intentional self harm 45](#_Toc230891131)

[Figure S31: Forest plots for Adults – Antidepressants anxiolytics; Adults – Antipsychotics; Adults – Psychotropic medication 46](#_Toc230891132)

[Figure S32. Meta-analytic associations of psychiatric and neurological indicators with ADHD medication discontinuation, excluding US data 47](#_Toc230891133)

[Figure S33. Multivariable country specific associations of psychiatric and neurological indicators with ADHD medication discontinuation (Australia, Denmark, Hong Kong, the Netherlands) 48](#_Toc230891134)

[Figure S34. Multivariable country specific associations of psychiatric and neurological indicators with ADHD medication discontinuation (Norway, Sweden, the United Kingdom, the United States) 49](#_Toc230891135)

[Figure S35. Meta-analytic associations of psychiatric and neurological indicators with ADHD medication discontinuation in males and females 50](#_Toc230891136)

[Figure S36. Sex-stratified associations of psychiatric and neurological indicators with ADHD medication discontinuation in Australia 51](#_Toc230891137)

[Figure S37. Sex-stratified associations of psychiatric and neurological indicators with ADHD medication discontinuation in Hong Kong 52](#_Toc230891138)

[Figure S38. Sex-stratified associations of psychiatric and neurological indicators with ADHD medication discontinuation in the Netherlands 53](#_Toc230891139)

[Figure S39. Sex-stratified associations of psychiatric and neurological indicators with ADHD medication discontinuation in Norway 54](#_Toc230891140)

[Figure S40. Sex-stratified associations of psychiatric and neurological indicators with ADHD medication discontinuation in Sweden 55](#_Toc230891141)

[Figure S41. Sex-stratified associations of psychiatric and neurological indicators with ADHD medication discontinuation in the UK 56](#_Toc230891142)

[Figure S42. Sex-stratified associations of psychiatric and neurological indicators with ADHD medication discontinuation in the US 57](#_Toc230891143)

[Figure S43. Meta-analytic associations of psychiatric and neurological indicators with ADHD medication discontinuation in individuals with a second ADHD medication dispensation within 180 days of initiation 58](#_Toc230891144)

[Figure S44. Country specific associations of psychiatric and neurological indicators with ADHD medication discontinuation in individuals with a 2^nd^ dispensation within 180 days of initiation (Australia, Hong Kong, the Netherlands, Norway) 59](#_Toc230891145)

[Figure S45. Country specific associations of psychiatric and neurological indicators with ADHD medication discontinuation in individuals with a 2^nd^ dispensation within 180 days of initiation (Sweden, the United Kingdom, the United States) 60](#_Toc230891146)

[Figure S46. Meta-analytic associations of psychiatric and neurological indicators defined as life-time with ADHD medication discontinuation 61](#_Toc230891147)

[Figure S47. Country specific associations of psychiatric and neurological indicators defined as life-time with ADHD medication discontinuation (Australia, Hong Kong, the Netherlands, Norway) 62](#_Toc230891148)

[Figure S48. Country specific associations of psychiatric and neurological indicators defined as life-time with ADHD medication discontinuation (Sweden, the United Kingdom, the United States) 63](#_Toc230891149)

## Table S1. Summary of healthcare setting, population coverage, and medication reimbursement policies for the different countries/region

| **Country** | **System Type** | **Primary Payer** | **ADHD reimbursement pathway** | **Patient Cost** | **Prescribing Requirements** | **Key Restrictions** |
| --- | --- | --- | --- | --- | --- | --- |
| Australia | Universal | Public | Listed on Pharmaceutical Benefits Scheme (PBS); authority approval required | Low fixed co-pay | Specialist initiation required, GP continuation allowed | Step therapy^1^; strict listing criteria^2^; diagnosis-specific reimbursement |
| Denmark | Universal + co-pay reimbursement | Public | Progressive reimbursement based on annual spend; Requires individual reimbursement application | Starts high → decreases as annual spend rises | Specialist initiation required, GP continuation allowed | Many ADHD drugs need case-by-case approval |
| Hong Kong | Mixed public/private (only public sector data included in study) | Public + out-of-pocket | Public system provides subsidized medications (limited formulary) | Low (public) / High (private) | Specialist prescribing required in public system ^3^ | Limited medication options in public system; prescribing controls |
| Netherlands | Mandatory insurance | Private insurers (regulated) | Covered under basic insurance package | Deductible applies, then covered | Specialist initiation required, GP continuation allowed | Insurer may prefer generic options |
| Norway | Universal (full or partial cost coverage) | Public | Reimbursed under approved diagnoses via national scheme | Co-pay until annual cap, then free | Specialist initiation required, GP continuation allowed | Step therapy; strict listing criteria; diagnosis-specific reimbursement; |
| Sweden | Universal (full or partial cost coverage) | Public | Included under National drug benefit scheme | Co-pay until annual cap, then free. Free for children < 18 years. | Specialist prescribing only | Step therapy; strict listing criteria; diagnosis-specific reimbursement; prescribing controls |
| UK | Universal (NHS) | Public | Covered under NHS | Low flat fee in England, free in Scotland and Wales. Free for children < 16 year, or < 19 years & in full-time education | Specialist initiation required, GP continuation allowed | Step therapy; guideline-based prescribing; local NHS formulary may add further restrictions |
| USA | Fragmented (mixed public/private /uninsured) | Private insurers; Medicaid/CHIP; Medicare; self-pay (uninsured) | Varies by payer; Covered under Medicaid/CHIP; Private insurance uses formulary tiers and prior authorization; uninsured pay out-of-pocket (possible access via discount programs or assistance) | Highly variable (minimal with Medicaid/CHIP → very high for uninsured/self-pay) | Prescribed by primary care or specialists | Prior authorization; step therapy; tiered formularies; quantity limits; state/federal controlled substance regulations; limited access for uninsured |

**Note**: ^1^ Step therapy refers to a practice where patients are required to try preferred medications first before being considered for alternative, non-preferred (i.e., not first-line) treatments. ^2^ Strict listing refers to the classification of ADHD medications as controlled substances due to abuse potential. Consequently, these medications are tightly regulated, restricting automatic or early refills and the lenght of prescirptions. ^3^ Specialist involvement is required in the diagnostic process, particularly within the public healthcare system. Further, unlike other participating countries, Hong Kong currently lacks established guidelines, specialist services, and a structured care pathway for adult ADHD.

##

## Table S2. Exclusion criteria and numbers retained at each step, by country/region

| Country | Study base | 2 years washout | Death | Emigration | Missing values | At least 4 years | Max 60 years |
| --- | --- | --- | --- | --- | --- | --- | --- |
| Australia | 48 095 | 31 288 | 31 274 | 31 274 | 31 273 | 31 273 | 30 295 |
| Denmark | 125 879 | 89 469 | 89 453 | 82 537 | 82 537 | 82 523 | 78 677 |
| Hong Kong | 39 899 | 29 507 | 29 504 | 29 504 | 29 504 | 29 499 | 28 954 |
| Netherlands <18 | 203 150 | 95 170 | 95 170 | 93 265 | 93 265 | 93 057 | 93 057 |
| Netherlands ≥18 | 291 453 | 218 910 | 218 895 | 199 071 | 199 071 | 199 071 | 183 637 |
| Norway | 82 818 | 53 543 | 53 541 | 53 375 | 53 375 | 53 365 | 53 365 |
| Sweden | 256 329 | 203 413 | 203 413 | 196 505 | 196 505 | 196 428 | 194 591 |
| United Kingdom | 32 412 | 12 637 | 12 637 | 12635 | 12 633 | 12 630 | 12 515 |
| United States | 816 712 | 355 783 | 355 344 | 355 344 | 355 344 | 354 516 | 325 320 |

**Note**: In the Netherlands, linked data on children/adolescents <18 years was available from 2011-2014, and data on individuals above ≥18 years from 2011 to 2020. Numbers are therefore shown separately.

## Table S3. Definition of clinical characteristics

| **Clinical characteristic** | **Definition ICD-10 and / or ATC** |
| --- | --- |
| Substance use disorders (SUD) | F10-F16, F18-F19 or ≥ 2 dispensation for anti-addiction agents [N07BB, N07BC] |
| Schizophrenia spectrum disorders | F20-F29 |
| Bipolar disorder | F30-31 or ≥ 2 dispensation for lithium [N05AN01] |
| Major depression | F32-F33 |
| Neurotic, stress-related, and somatoform disorders | F40-F48, F93 |
| Anxiety disorder | F40-F41, F93 |
| Obsessive compulsive disorder (OCD) | F42 |
| Eating disorders | F50 |
| Personality disorders | F60-F61 |
| Borderline personality disorder (BPD | F60.3 |
| Intellectual disability (ID) | F70-F79 |
| Autism spectrum disorder | F84 |
| Conduct disorder/Oppositional defiant disorder (CD/ODD) | F91 |
| Tic disorders | F95 |
| Epilepsy | G40-G41 |
| Migraine | G43 or ≥ 2 dispensation for triptans [N02CC] |
| Sleep disorders | G47 or ≥ 2 dispensation for benzodiazepine related sleeping medication [N05CF] |
| Psychiatric multimorbidity | ≥ 1 diagnoses in at least 2 different ICD-10 F blocks (F0, F1, F2, F3, F4, F5, F6, F7, F8, F9) excluding ADHD [F90x] |
| Psychiatric inpatient admission | ≥ 1 diagnoses with an F00-F99 code (F0, F1, F2, F3, F4, F5, F6, F7, F8, F9 excluding ADHD [F90x]) in inpatient care |
| Suicide attempt/self-harm | X60-X84, Y10-Y34, Y87.0, Y87.2 |
| Antidepressants/anxiolytics | ≥ 2 dispensations of N05B and N06A |
| Antipsychotics | ≥ 2 dispensations of antipsychotics [N05A] |
| Psychotropic polypharmacy | ≥ 1 dispensed medication in any of the following ATC groups: N03A [Antiepileptics], N05A[Antipsychotics], N05B [Anxiolytics], N05C [Hypnotics and sedatives], N06A [Antidepressants], N07B [anti-addiction agents]) in the 3 months prior to ADHD medication initiation |

**Note**: We included medication dispensations as disorder proxies for drugs with only one indication, i.e. anti-addiction medications for substance use disorders, lithium for bipolar disorder, triptans for migraine, and sleep medications for sleep disorders.

## Table S4. ADHD medication at initiation across countries/regions

| ADHD medication at initiation, n (%) | Australia^a^  N=30 295 | Denmark  N=78 677 | Hong Kong  N=28 954 | Netherlands < 18^b^  N=93 057 | Netherlands ≥18^b^  N=183 637 | Norway N=53 365 | Sweden  N=194 591 | UK  N=12 515 | USA  N=352 320 |
| --- | --- | --- | --- | --- | --- | --- | --- | --- | --- |
| Methylphenidate | 13 913 (45.93) | 68 743 (87.37) | 28 012 (96.75) | 93 057 (100.00) | 183 637 (100.00) | 49 952 (93.60) | 162 787 (83.66) | 10 818 (86.44) | 105 863 (32.54) |
| Amfetamine | na | 61 (0.08) | na | na | na | 35 (0.07) | 471(0.24) | na | 120 123 (36.92) |
| Dexamfetamine | 12 208 (40.30) | 153 (0.19) | na | na | na | 376 (0.71) | 548 (0.28) | 114 (0.91) | 111 849 (34.38) |
| Lisdexamfetamine | 1 757 (5.8) | 792 (1.01) | na | na | na | 1 294 (2.43) | 12 551 (6.45) | 552 (4.41) | 52 472 (16.13) |
| Atomoxetine | 2 277 (7.52) | 8 942 (11.37) | 1 112 (3.84) | na | na | 2 272 (4.26) | 17 007 (8.74) | 994 (7.94) | 22 571 (6.94) |
| Guanfacine | 216 (0.71) | 29 (0.04) | na | na | na | 23 (0.04) | 1 571 (0.81) | 75 (0.60) | 33 321 (10.24) |

**Note**. An individual could have more than one type of medication at initiation. ^a^ Linked data in Australia was only available individuals ≥18 years. ^b^ In the Netherlands, linked data on children/adolescents <18 years was available from 2011-2014, and data on individuals above ≥18 years from 2011 to 2020. na, not available due to lack of data in age groups [Australia, Netherlands], lack of specific ATC codes [Netherlands], or due the medication not being used for ADHD in that country/region [Australia, Hong Kong, United Kingdom].

## Table S5. Follow-up time and medication discontinuation summary by country and age-group

| Country/  Region | Age group | n total | Discontinuation (n, %) | n person-years | Follow-up time (median, IQR) | Time to discontinuation (median, IQR) |
| --- | --- | --- | --- | --- | --- | --- |
| Australia | Young adults (18-24) | 8 038 | 5 886 (73%) | 7 688 | 318 (199, 545) | 249 (180, 345) |
|  | Adults (25-60) | 22 257 | 15 056 (68%) | 22 346 | 344 (210, 545) | 253 (180, 350) |
| Denmark | Children (4-11) | 16 673 | 3 107 (19%) | 20 760 | 545 (378, 545) | 276 (207, 386) |
|  | Adolescents (12-17) | 14 356 | 5 355 (37%) | 16 067 | 495 (267, 545) | 298 (221, 401) |
|  | Young adults (18-24) | 16 530 | 8 889 (54%) | 16 109 | 338 (207, 545) | 252 (192, 354) |
|  | Adults (25-60) | 31 118 | 13 409 (43%) | 31 457 | 386 (202, 545) | 241 (180, 347) |
| Hong Kong | Children (4-11) | 24 048 | 11 719 (49%) | 25 343 | 420 (223, 545) | 278 (180, 390) |
|  | Adolescents (12-17) | 3 488 | 2 079 (60%) | 3 380 | 347 (204, 545) | 271 (180, 378) |
|  | Young adults (18-24) | 490 | 280 (57%) | 446 | 295 (180, 545) | 237 (180, 334) |
|  | Adults (25-60) | 928 | 419 (45%) | 755 | 250 (180, 529) | 222 (180, 326) |
| Netherlands | Children (4-11) | 59 719 | 16 144 (27%) | 63 764 | 457 (229, 545) | 251 (196, 362) |
|  | Adolescents (12-17) | 33 338 | 16 913 (50%) | 30 586 | 306 (201, 545) | 251 (198, 349) |
|  | Young adults (18-24) | 54 038 | 36 145 (67%) | 48 655 | 284 (194, 515) | 237 (187, 333) |
|  | Adults (25-60) | 129 599 | 74 240 (57%) | 121 661 | 306 (192, 545) | 225 (180, 325) |
| Norway | Children (4-11) | 18 024 | 4 312 (24%) | 21 716 | 545 (331, 545) | 272 (209, 392) |
|  | Adolescents (12-17) | 10 386 | 5 200 (50%) | 10 853 | 393 (241, 545) | 300 (211, 392) |
|  | Young adults (18-24) | 7 439 | 4 044 (54%) | 7 349 | 350 (211, 545) | 271 (209, 364) |
|  | Adults (25-60) | 17 516 | 7 096 (41%) | 18 667 | 425 (239, 545) | 271 (208, 364) |
| Sweden | Children (4-11) | 51 412 | 11 452 (22%) | 61 590 | 545 (317, 545) | 280 (205, 397) |
|  | Adolescents (12-17) | 52 985 | 22 775 (43%) | 56 691 | 429 (248, 545) | 300 (220, 401) |
|  | Young adults (18-24) | 25 813 | 13 296 (52%) | 26 398 | 381 (217, 545) | 263 (194, 371) |
|  | Adults (25-60) | 64 381 | 24 290 (38%) | 70 547 | 488 (237, 545) | 262 (187, 376) |
| UK | Children (4-11) | 6 932 | 2 324 (34%) | 8 438 | 545 (337, 545) | 274 (180, 398) |
|  | Adolescents (12-17) | 2 985 | 1 506 (50%) | 3 274 | 481 (240, 545) | 264 (180, 392) |
|  | Young adults (18-24) | 867 | 543 (63%) | 759 | 312 (180, 545) | 239 (180, 341) |
|  | Adults (25-60) | 1 731 | 940 (54%) | 1 704 | 377 (212, 545) | 262 (180, 362) |
| USA | Children (4-11) | 90 084 | 54 615 (61%) | 82 005 | 286 (180, 545) | 203 (180, 313) |
|  | Adolescents (12-17) | 56 393 | 38 087 (68%) | 43 856 | 208 (180, 430) | 181 (180, 288) |
|  | Young adults (18-24) | 39 556 | 26 539 (67%) | 30 053 | 199 (180, 405) | 180 (180, 280) |
|  | Adults (25-60) | 139 287 | 94 816 (68%) | 108 987 | 199 (180, 437) | 180 (180, 271) |

**Note**: Discontinuation was defined as a ≥180-day gap between dispensations, with the initial dispensation assumed to last 180 days. To qualify as early discontinuation, the last dispensation had to occur within 365 days of initiation, but the discontinuation date could fall within 1.5 years after initiation, making the time to discontinuation restricted to minimum 180 days and maximum 545 days.

## Table S6. Descriptive statistics of individuals with a second ADHD medication dispensation within 180 days of initiation

| N (%) | Australia ^a^ | Hong Kong | Netherlands < 18^b^ | Netherlands ≥18^b^ | Norway | Sweden | UK | USA |
| --- | --- | --- | --- | --- | --- | --- | --- | --- |
| N total (% of full study population) | 24 034 (79%) | 24 125  (83%) | 86 376  (93%) | 152 996 (83%) | 48 386 (91%) | 179 014 (92%) | 11 048 (88%) | 207 409 (64%) |
| ADHD diagnosis | 1 143 (4.76%) | 18 484 (76.62%) | 49 733 (57.58%) | 84 972 (55.54%) | 44 109 (91.16%) | 159 408 (89.05%) | 8 980 (81.28%) | 173 947 (83.87%) |
| Sex |  |  |  |  |  |  |  |  |
| *Female* | 13 876 (57.73%) | 5 362 (22.23%) | 60 919 (70.53%) | 80 312 (52.49%) | 19 521 (40.34%) | 74 898 (41.84%) | 2 529 (22.89%) | 109 387 (52.74%) |
| *Male* | 10 158 (42.27%) | 18 763 (77.77%) | 25 457 (29.47%) | 72 684 (47.51%) | 28 865 (59.66%) | 104 116 (58.16%) | 8 519 (77.11%) | 98 022 (47.26%) |
| Age category |  |  |  |  |  |  |  |  |
| *Children (4-11)* | na | 19 992 (82.87%) | 56 063 (64.91%) | na | 16 705 (34.52%) | 48 366 (27.02%) | 6 278 (56.82%) | 63 974 (30.84%) |
| *Adolescents (12-17)* | na | 2 994 (12.41%) | 30 313 (35.09%) | na | 9 486 (19.60%) | 49 567 (27.69%) | 2 608 (23.61%) | 35 709 (17.22%) |
| *Young adults (18-24)* | 6 344 (26.40%) | 404 (1.67%) | na | 45 208 (29.55%) | 6 435 (13.30%) | 22 862 (12.77%) | 701 (6.35%) | 24 102 (11.62%) |
| *Adults (25-60)* | 17 690 (73.60%) | 735 (3.05%) | na | 107 788 (70.45%) | 15 760 (32.57%) | 58 219 (32.52%) | 1 461 (13.22%) | 83 624 (40.32%) |
| Age at first dispensing (mean, IQR) | 32 (25, 41) | 9.0 (7.8, 10.8) | 10.3 (8.2, 13.6) | 32 (24, 42) | 16 (10, 29) | 17 (12, 29) | 11 (8, 16) | 19 (10, 35) |
| ADHD medication at first dispensation ^c^ |  |  |  |  |  |  |  |  |
| *Methylphenidate* | 10 303 (42.87%) | 22 970 (95.21%) | 86 376 (100.00%) | 152 996 (100.00%) | 43 214 (89.31%) | 144 329 (80.62%) | 9 556 (86.50%) | 68 019 (32.79%) |
| *Amfetamine* | na | na | na | na | 101 (0.21%) | 384 (0.21%) | na | 76 816 (37.04%) |
| *Dexamfetamine* | 10 531 (43.82%) | na | na | na | 728 (1.50%) | 751 (0.42%) | 92 (0.83%) | 75 247 (36.28%) |
| *Lisdexamfetamine* | 1 601 (6.66%) | na | na | na | 2 780 (5.75%) | 17 014 (9.50%) | 505 (4.57%) | 33 442 (16.12%) |
| *Atomoxetine* | 1 529 (6.36%) | 1 380 (5.72%) | na | na | 2 538 (5.25%) | 15 575 (8.70%) | 854 (7.73%) | 11 447 (5.52%) |
| *Guanfacine* | 144 (0.60%) | na | na | na | 32 (0.07%) | 1 504 (0.84%) | 65 (0.59%) | 20 517 (9.89%) |

**Note**. ^a^ Linked data in Australia was only available individuals ≥18 years. ^b^ In the Netherlands, linked data on children/adolescents <18 years was available from 2011-2014, and data on individuals above ≥18 years from 2011 to 2020. Numbers are therefore shown separately. ^c^ An individual could have more than one type of medication at initiation. na, not available due to lack of data in age groups [Australia, Netherlands], lack of specific ATC codes [Netherlands], or due the medication not being used for ADHD in that country/region [Australia, Hong Kong, United Kingdom]. Denmark did not contribute to secondary analyses due to regulations regarding data extraction.

## Table S7. Ethical approval and data sharing statement by country

| Country/region | Ethical Review Oversight | Data sharing statement |
| --- | --- | --- |
| Australia | This research received ethical approval from the AIHW Human Research Ethics Committee (AIHW HREC) (approval number EO2021/1/1233) and the NSW Population and Health Services Research Ethics Committee (PHSREC) (approval number 2020/ETH02273). | Direct access to the data and analytical files to other individuals or authorities is not permitted without the express permission of the approving human research ethics committees and data custodians. |
| Denmark | The study was approved by the Danish Data Protection Agency, and data access was agreed by Statistics Denmark and the Danish Health Data Authority. Approval by the Ethics Committee and written informed consent were not required for register-based projects, cf. LBK nr 1338 af 01/09/2020, Act no. 1338 of 1 September 2020, section 10 on research ethics for administration of health scientific research projects and health data scientific research projects. | The data that support the findings of this study are available from Statistics Denmark. The data access required the completion of a detailed application form from the Danish Data Protection Agency, the Danish National Board of Health and Statistics Denmark. Researchers at Danish research institutions may obtain the relevant approval and data. International researchers may gain data access if governed by a Danish research institution having needed approval and data access. For more information on accessing the data, see http://www.dst.dk/. |
| Hong Kong SAR | The study was approved by the institutional review board of the  University of Hong Kong/Hospital Authority Hong Kong West Cluster  (reference no. UW 12–136). | Individual-level data is prohibited for sharing or being publicly available due to patient confidentiality and  privacy concerns. Data could be obtained by application to the Hong Kong Hospital Authority data sharing portal (https://  www3.ha.org.hk/data). |
| Netherlands | Analyses of administrative health records are exempt from the requirement of informed consent. Individual-level data was anonymized and did not leave the Statistics Netherlands server. | This study was performed under project agreement 9205 microdata services research. Individual-level data is prohibited for sharing or being publicly available. Data can be obtained by application to Statistics Netherlands. |
| Norway | The study was approved by the Regional Ethics Committee in Western Norway (2020/75421).  Informed consent is not required for pseudo anonymized register-based research. | National regulations and laws prohibit us from sharing or making individual-level data publicly available. The data can be obtained by application to the Health Data Service at the Norwegian Institute of Public Health: <https://helsedata.no/no/helsedataservice/>  and one of the Norwegian Regional Committees for Medical and Health Research Ethics. |
| Sweden | This study was approved by the Swedish Ethical Review Authority (2020-06540). Informed consent is not required for pseudo anonymized register-based research according to Swedish law. | The Public Access to Information and Secrecy Act in Sweden prohibits us from making individual-level data publicly available. Researchers who are interested in replicating our work can apply for individual-level data through Statistics Sweden at: https://www.scb.se/en/services/guidance-for-researchers-and-universities/ |
| The United Kingdom | The study was approved by the Health Improvement Network Scientific Review Committee (17THIN102). | Individual-level data is prohibited for sharing or being publicly available. Data could be obtained by application to the Health Improvement Network. |
| The United States | The study was approved by the Institutional Review Board of the SUNY Upstate Medical University (1933232-2). | The individual-level electronic health record data in the TriNetX database cannot be shared, as they are the property of TriNetX and can only be accessed through direct contract with the company. |

## Figure S1. Percent with psychiatric and neurological indicators by discontinuation status at end of follow-up in Australia


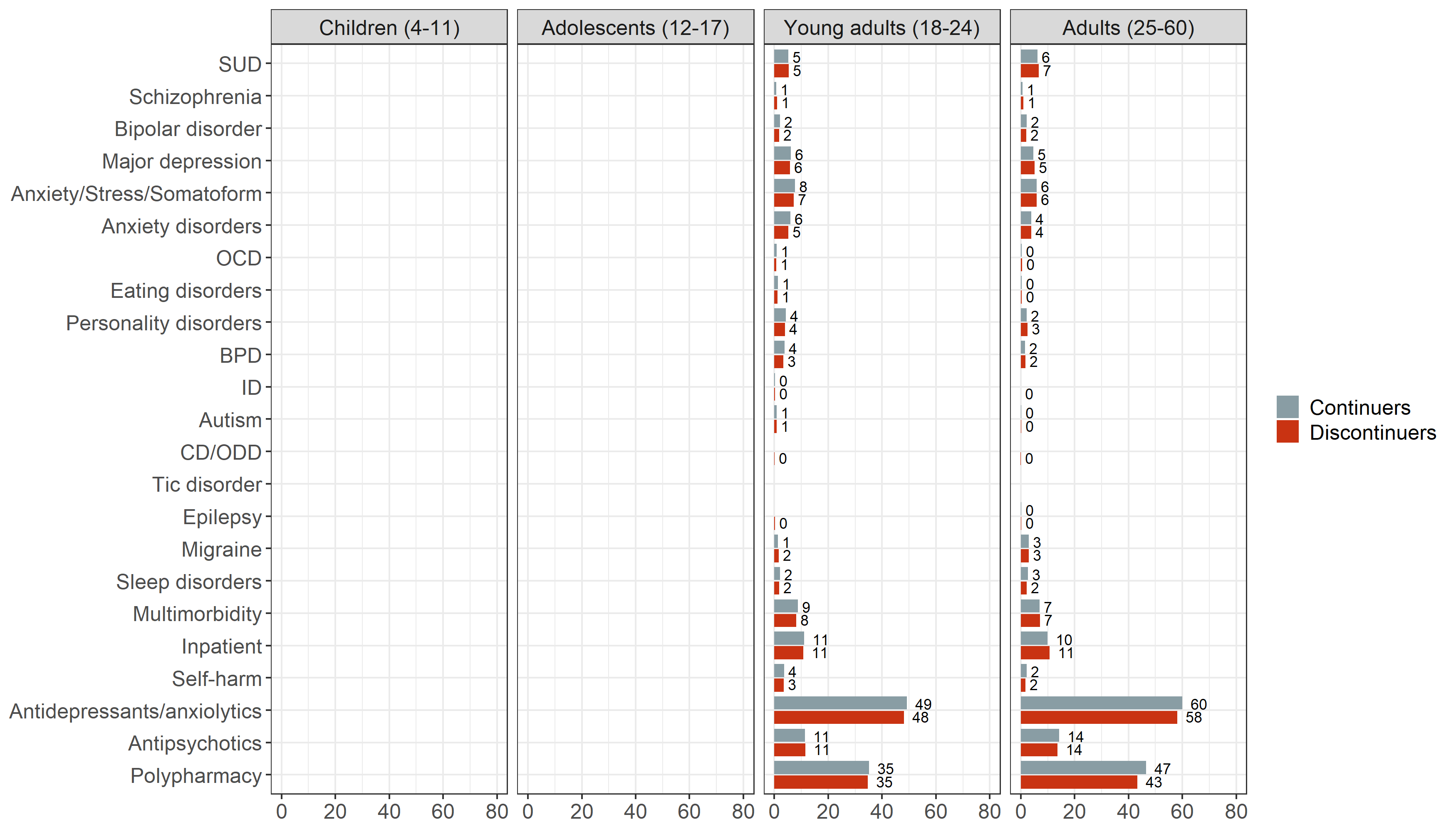


**Note:** Rows with <5 observations are blanked. SUD, substance use disorder; OCD, obsessive compulsive disorders; BPD, borderline personality disorder; ID, intellectual disability; CD/ODD, conduct disorder/oppositional defiant disorder; Inpatient, Psychiatric inpatient admission; Polypharmacy, psychotropic polypharmacy

## Figure S2. Percent with psychiatric and neurological indicators by discontinuation status at end of follow-up in Denmark


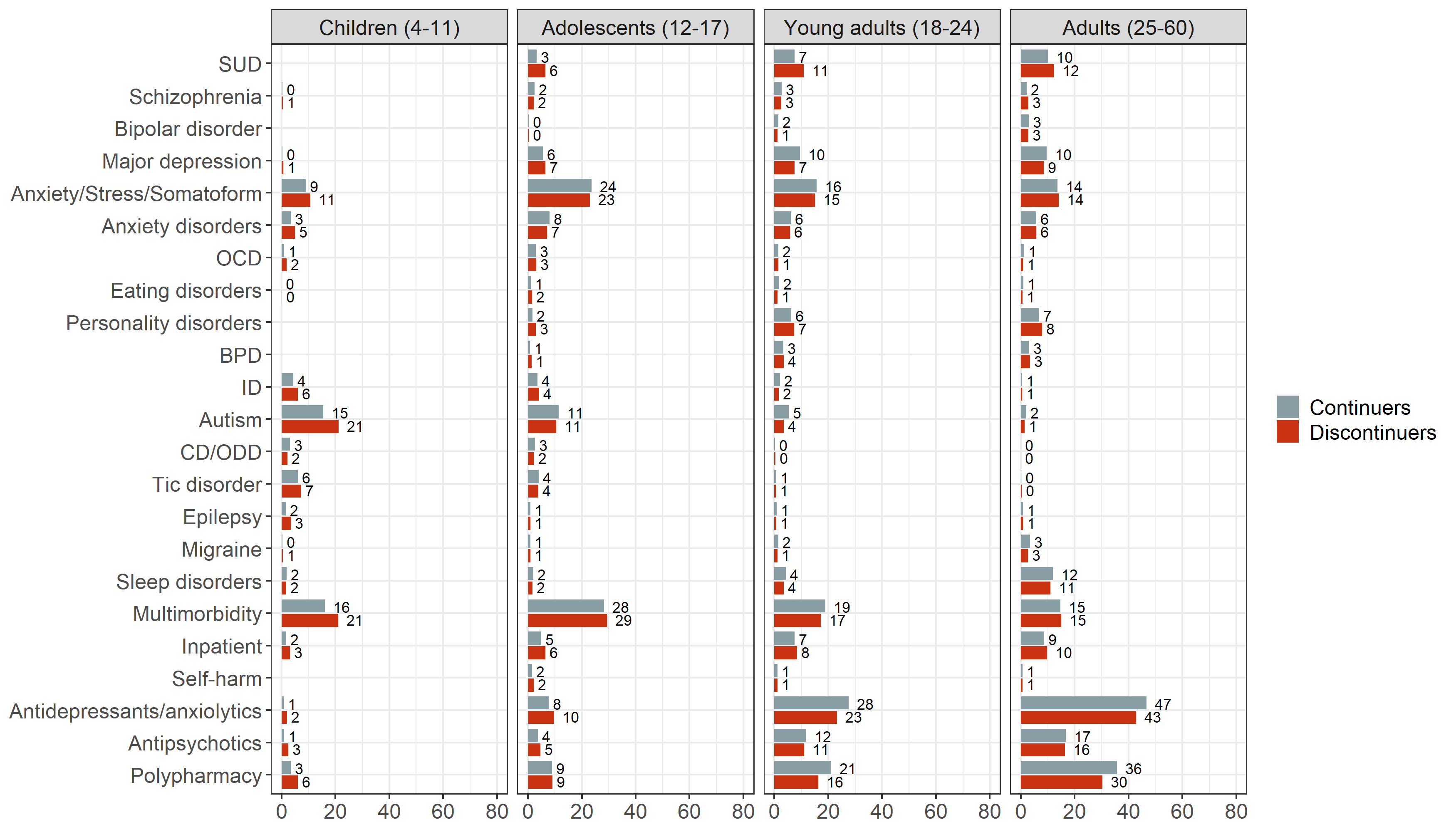


**Note:** Rows with <5 observations are blanked. SUD, substance use disorder; OCD, obsessive compulsive disorders; BPD, borderline personality disorder; ID, intellectual disability; CD/ODD, conduct disorder/oppositional defiant disorder; Inpatient, Psychiatric inpatient admission; Polypharmacy, psychotropic polypharmacy

## Figure S3. Percent with psychiatric and neurological indicators by discontinuation status at end of follow-up in Hong Kong


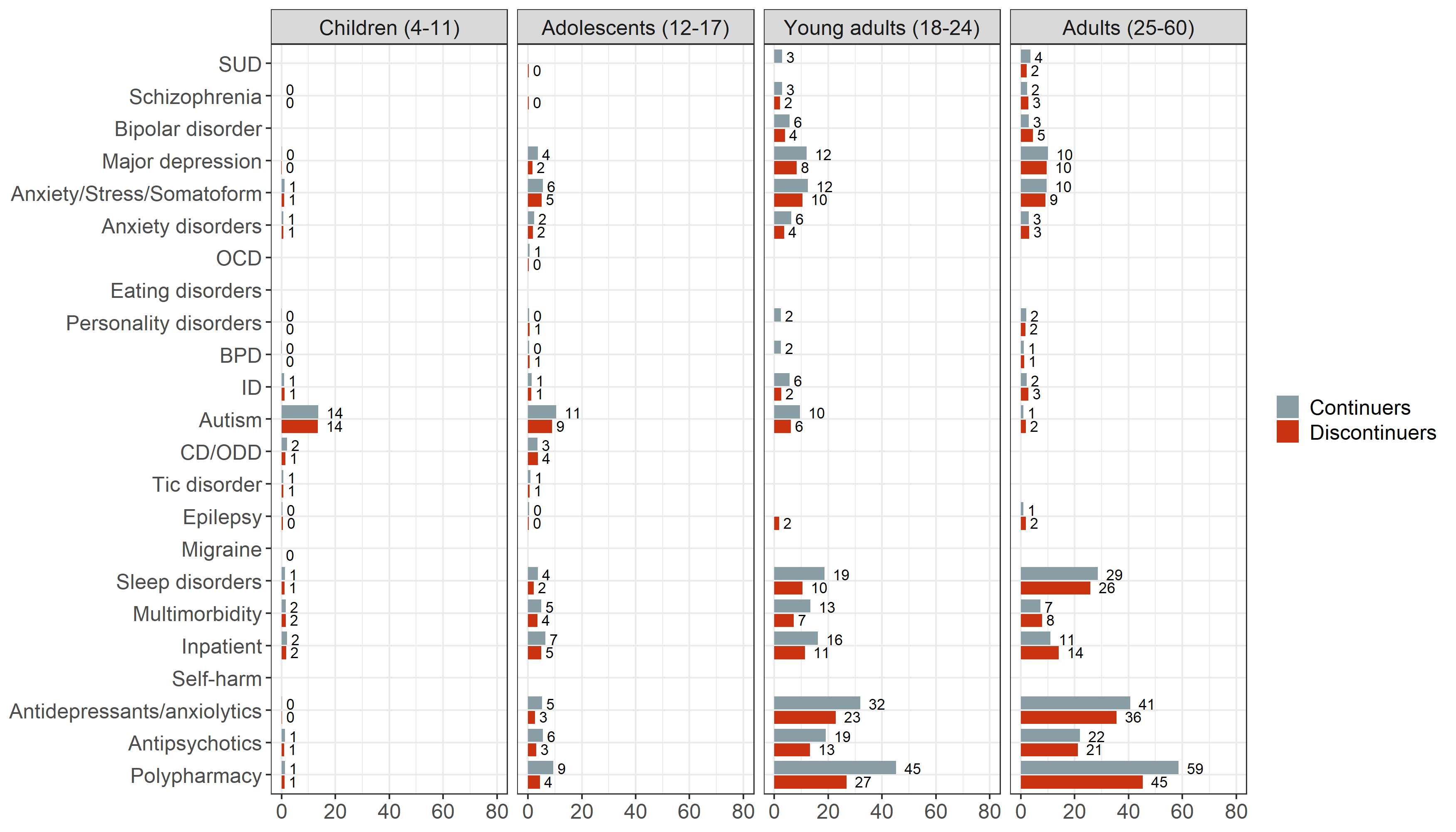


**Note:** Rows with <5 observations are blanked. SUD, substance use disorder; OCD, obsessive compulsive disorders; BPD, borderline personality disorder; ID, intellectual disability; CD/ODD, conduct disorder/oppositional defiant disorder; Inpatient, Psychiatric inpatient admission; Polypharmacy, psychotropic polypharmacy

## Figure S4. Percent with psychiatric and neurological indicators by discontinuation status at end of follow-up in the Netherlands


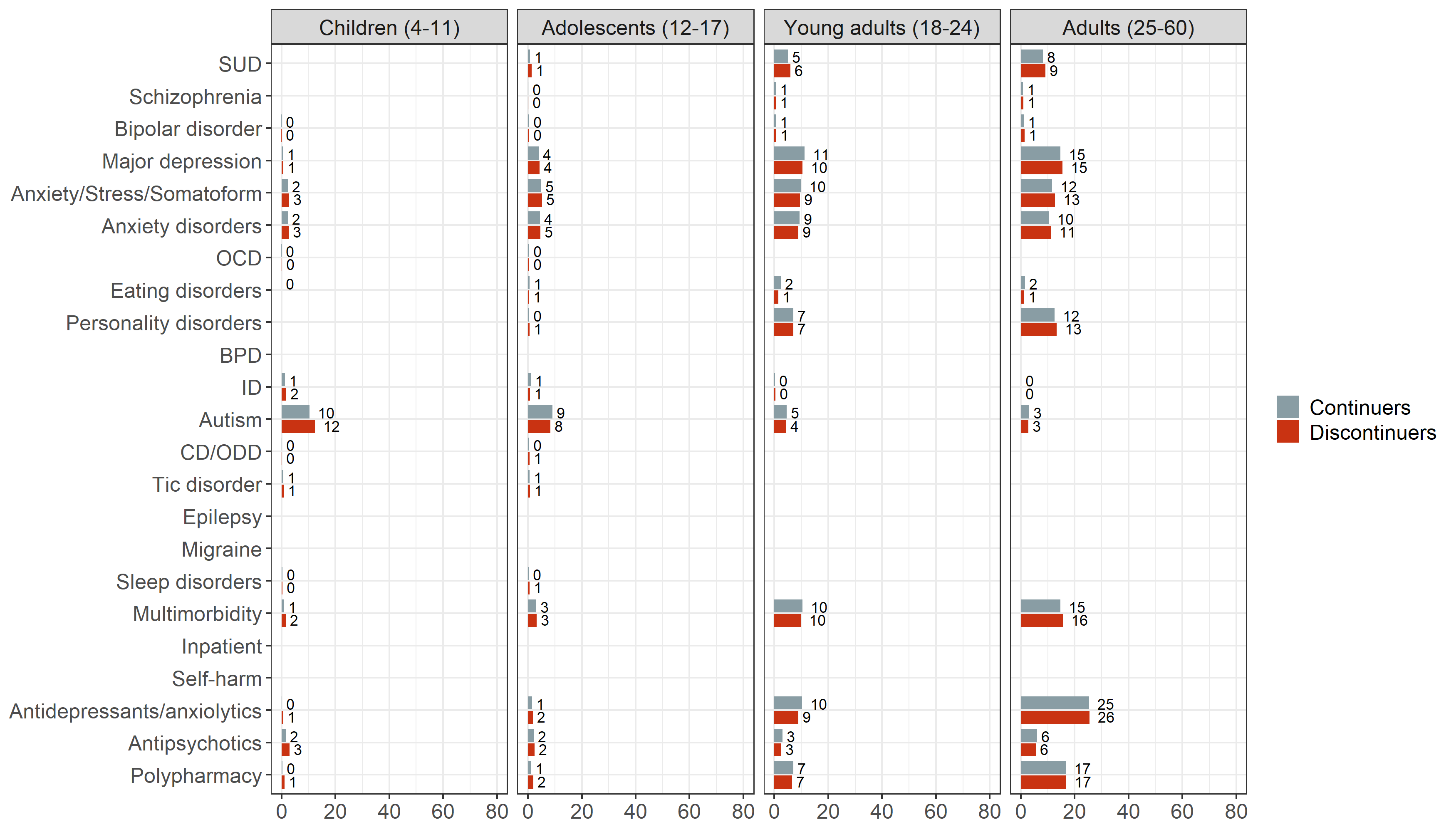


**Note:** Rows with <5 observations are blanked. SUD, substance use disorder; OCD, obsessive compulsive disorders; BPD, borderline personality disorder; ID, intellectual disability; CD/ODD, conduct disorder/oppositional defiant disorder; Inpatient, Psychiatric inpatient admission; Polypharmacy, psychotropic polypharmacy

## Figure S5. Percent with psychiatric and neurological indicators by discontinuation status at end of follow-up in Norway


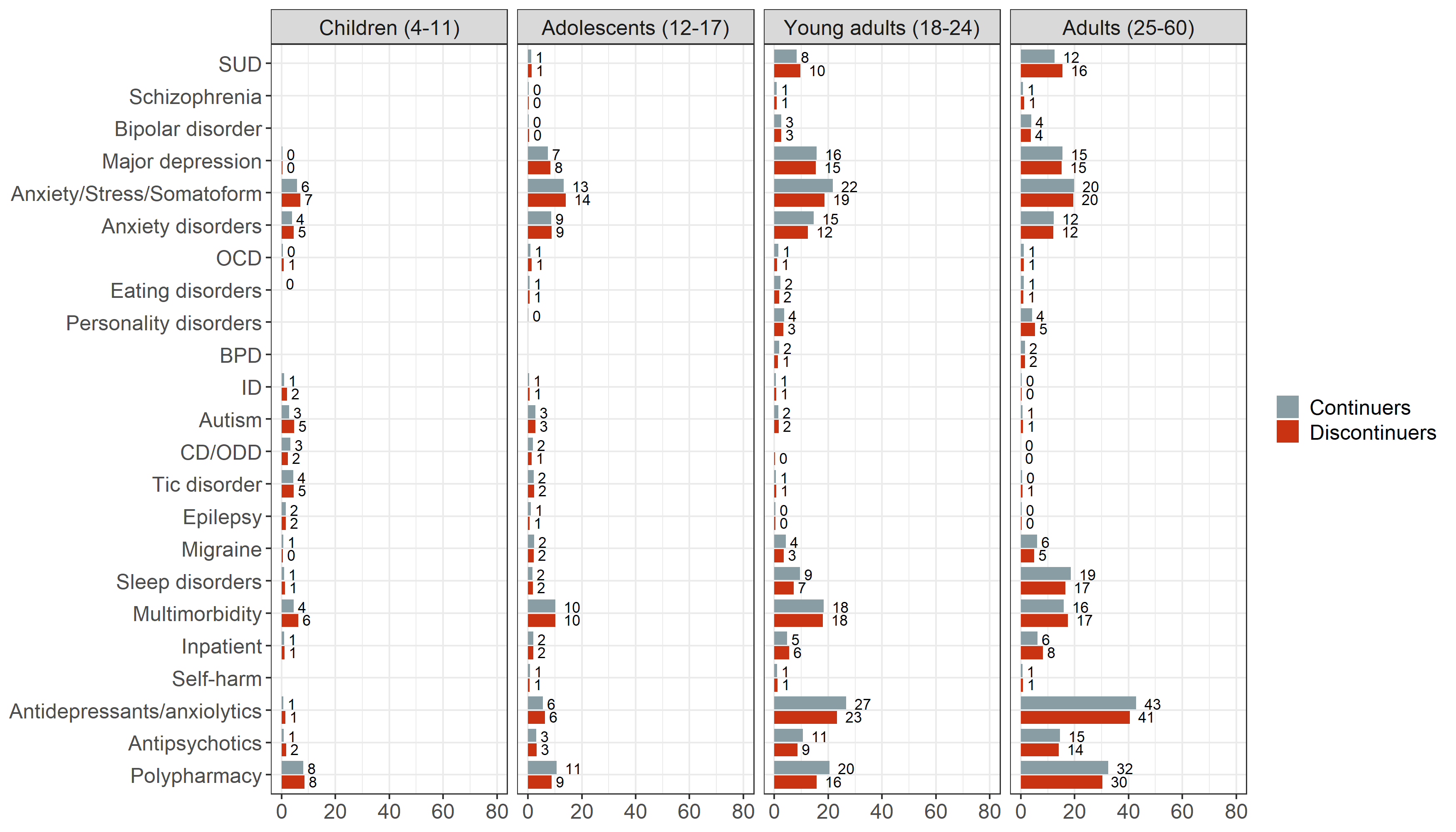


**Note:** Rows with <5 observations are blanked. SUD, substance use disorder; OCD, obsessive compulsive disorders; BPD, borderline personality disorder; ID, intellectual disability; CD/ODD, conduct disorder/oppositional defiant disorder; Inpatient, Psychiatric inpatient admission; Polypharmacy, psychotropic polypharmacy

## Figure S6. Percent with psychiatric and neurological indicators by discontinuation status at end of follow-up in Sweden


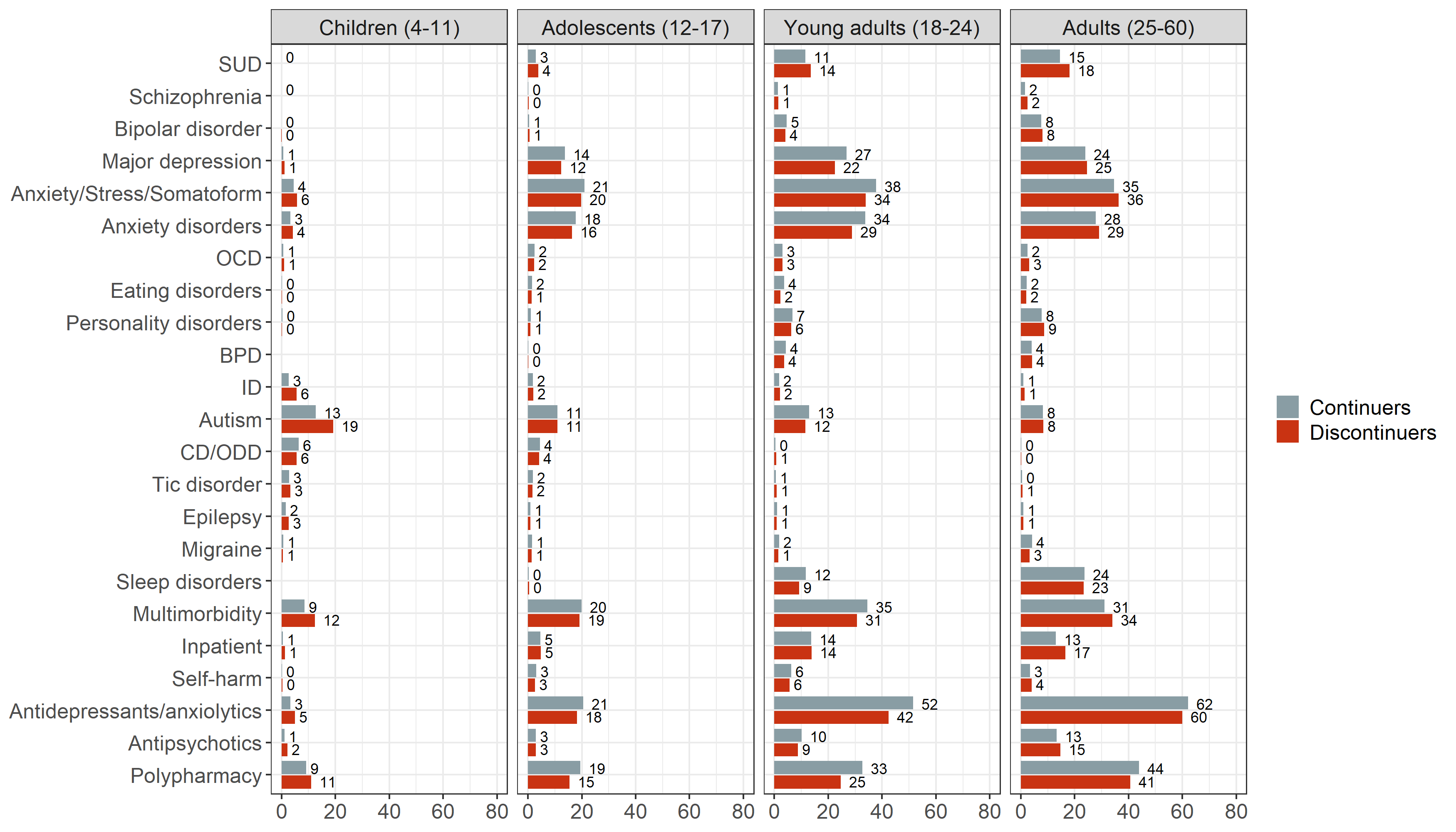


**Note:** Rows with <5 observations are blanked. SUD, substance use disorder; OCD, obsessive compulsive disorders; BPD, borderline personality disorder; ID, intellectual disability; CD/ODD, conduct disorder/oppositional defiant disorder; Inpatient, Psychiatric inpatient admission; Polypharmacy, psychotropic polypharmacy

## Figure S7. Percent with psychiatric and neurological indicators by discontinuation status at end of follow-up in the UK


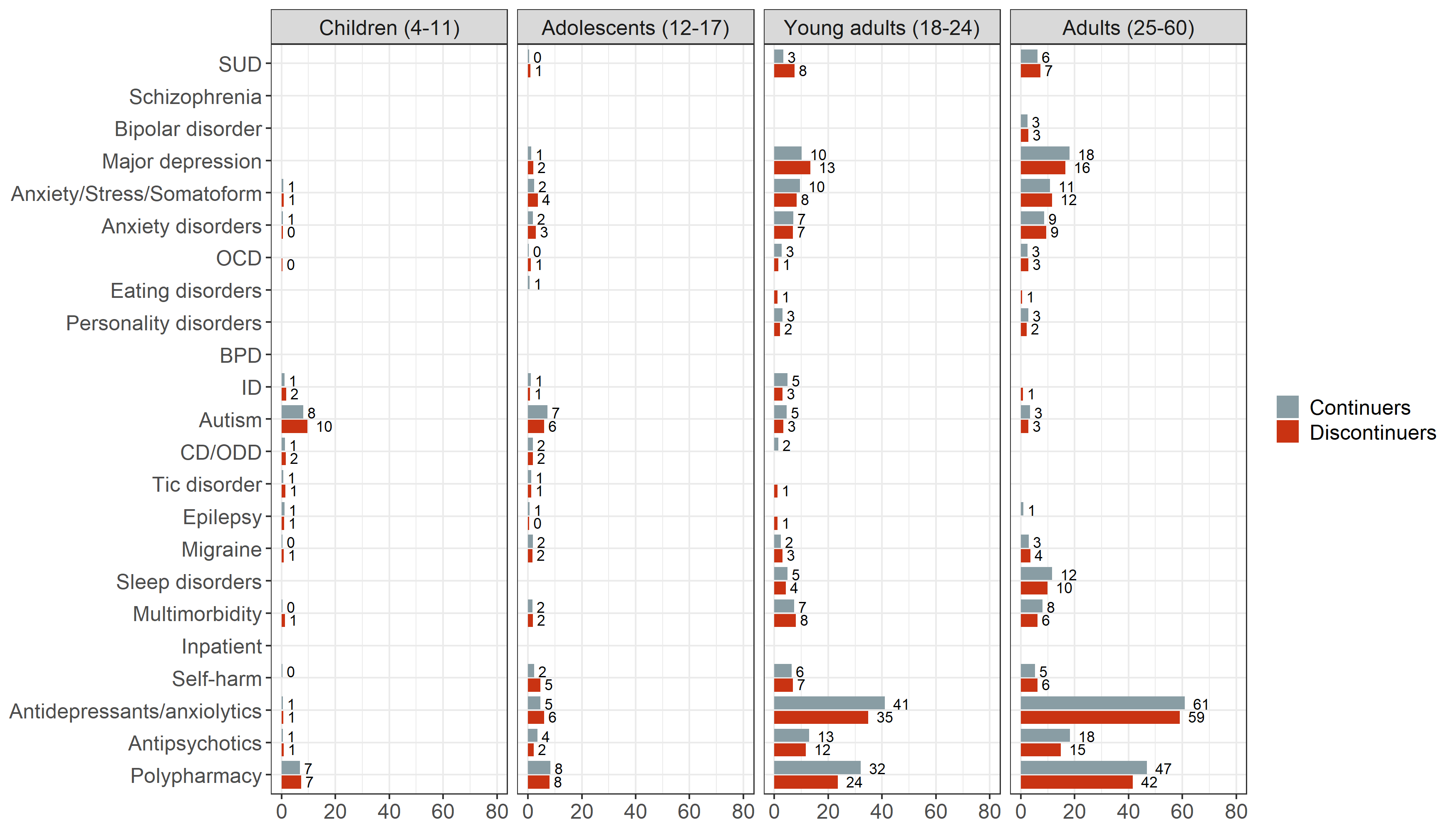


**Note:** Rows with <5 observations are blanked. SUD, substance use disorder; OCD, obsessive compulsive disorders; BPD, borderline personality disorder; ID, intellectual disability; CD/ODD, conduct disorder/oppositional defiant disorder; Inpatient, Psychiatric inpatient admission; Polypharmacy, psychotropic polypharmacy

## Figure S8. Percent with psychiatric and neurological indicators by discontinuation status at end of follow-up in US

**
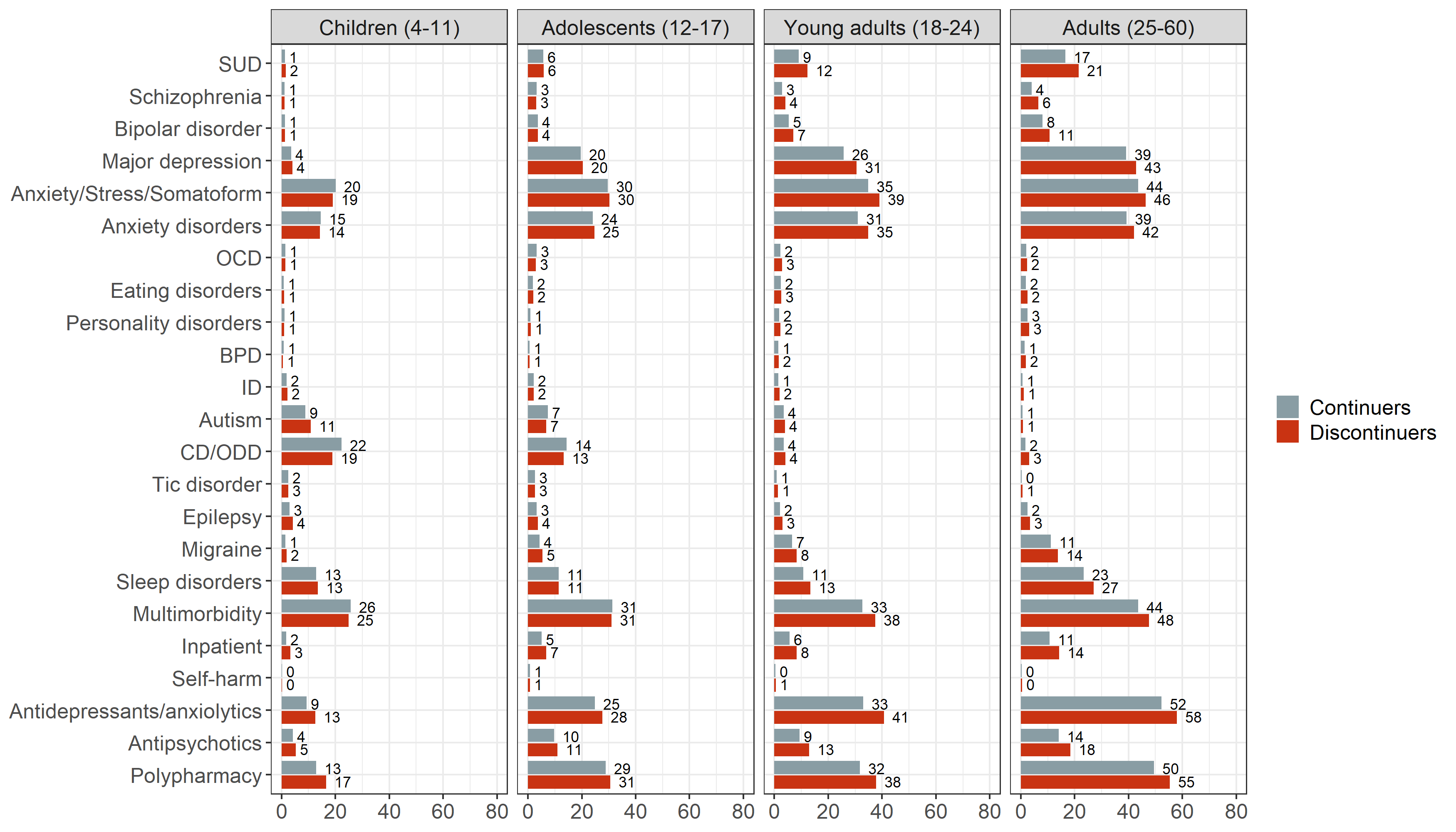
**

**Note:** Rows with <5 observations are blanked. SUD, substance use disorder; OCD, obsessive compulsive disorders; BPD, borderline personality disorder; ID, intellectual disability; CD/ODD, conduct disorder/oppositional defiant disorder; Inpatient, Psychiatric inpatient admission; Polypharmacy, psychotropic polypharmacy

## Figure S9: Forest plots for Children – Schizophrenia spectrum disorders; Children – Bipolar disorder; Children –Major depression; Children – Neurotic, stress related, and somatoform disorders


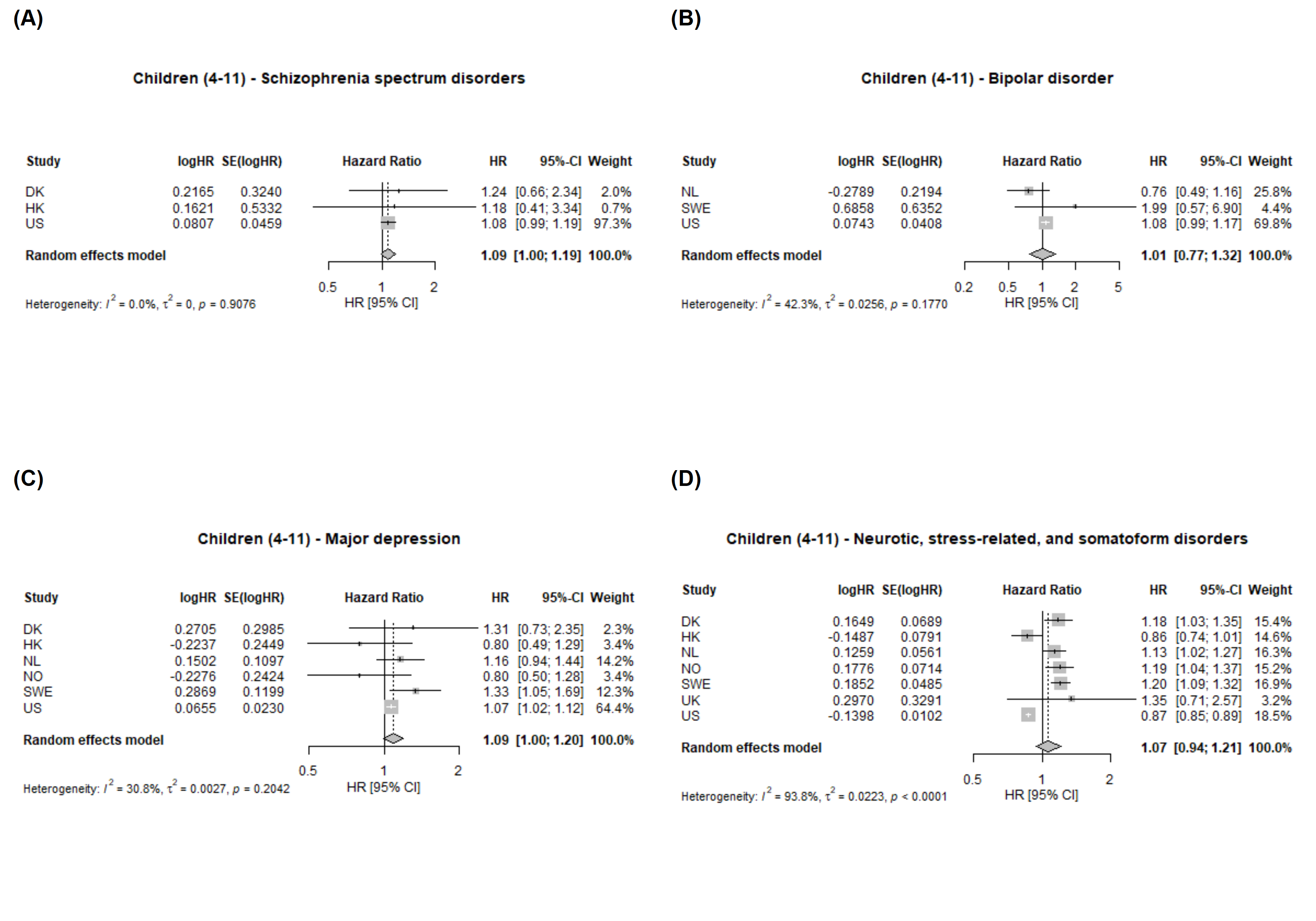


## Figure S10: Forest plots for Children – Anxiety disorders; Children – Obsessive compulsive disorder; Children – Eating disorders; Children – Personality disorders


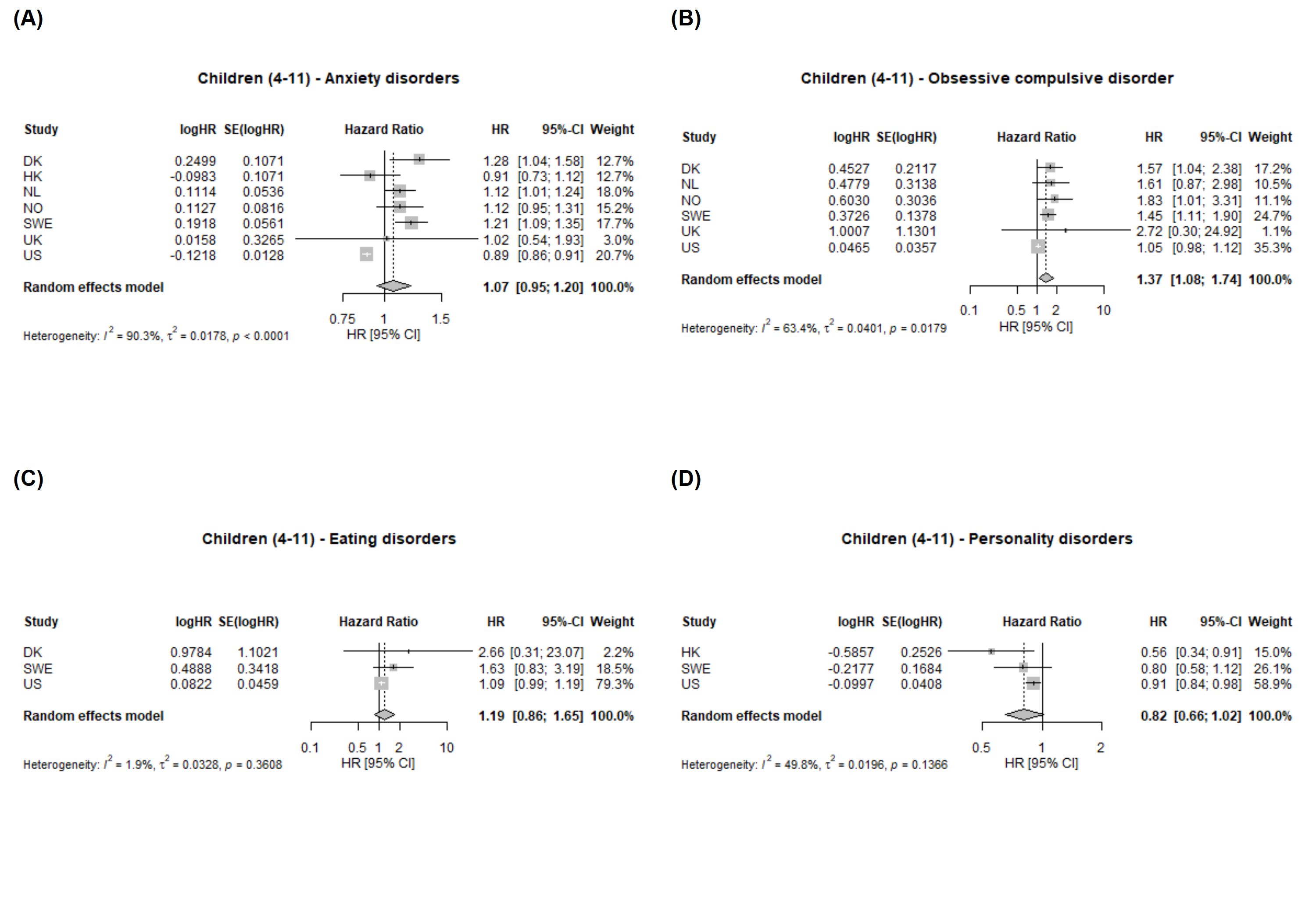


## Figure S11: Forest plots for Children – Borderline personality disorder; Children – Intellectual disability; Children – Autism spectrum disorder; Children – Conduct disorders


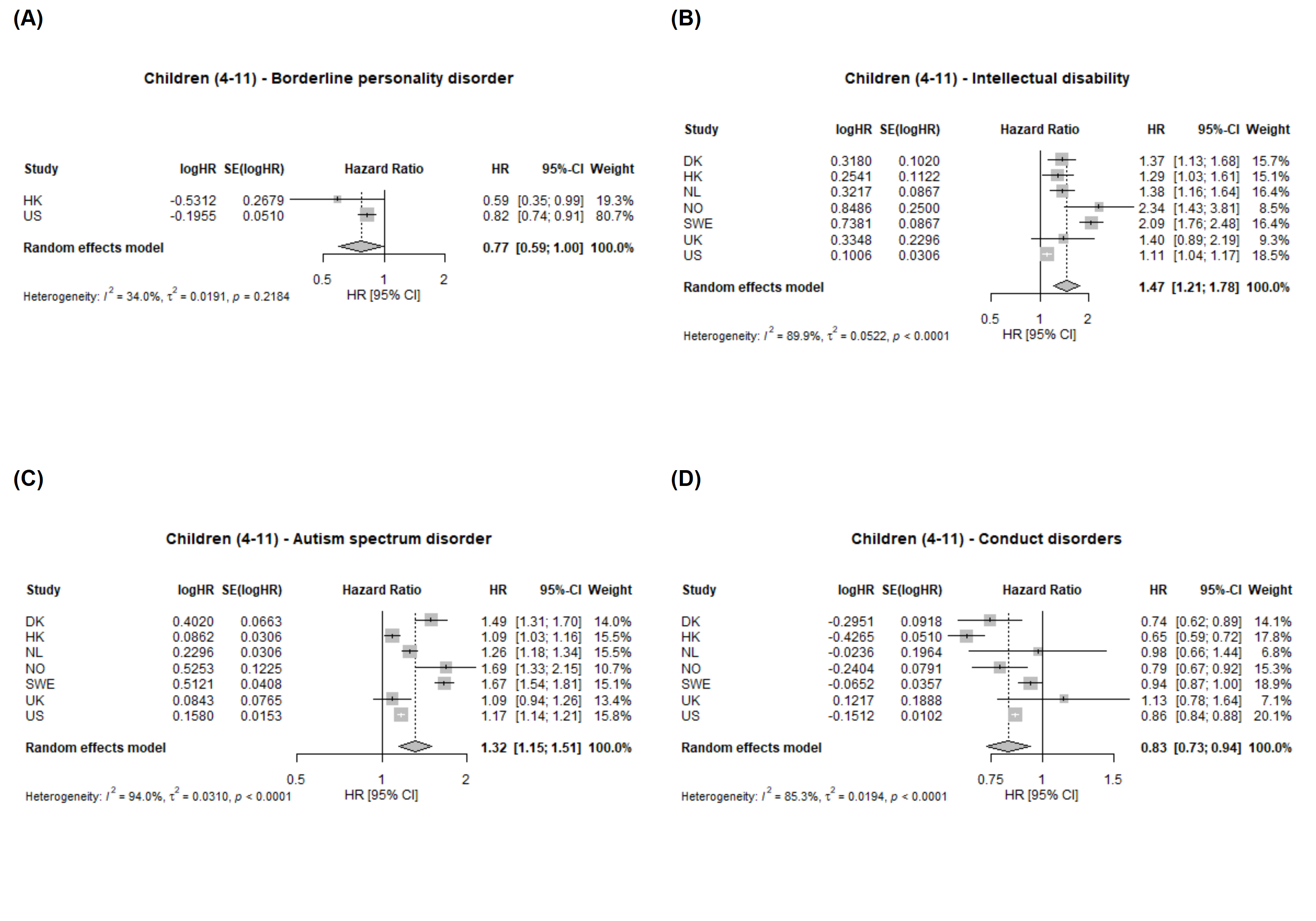


## Figure S12: Forest plots for Children – Tic disorder; Children – Epilepsy; Children – Migraine; Children – Sleep disorders


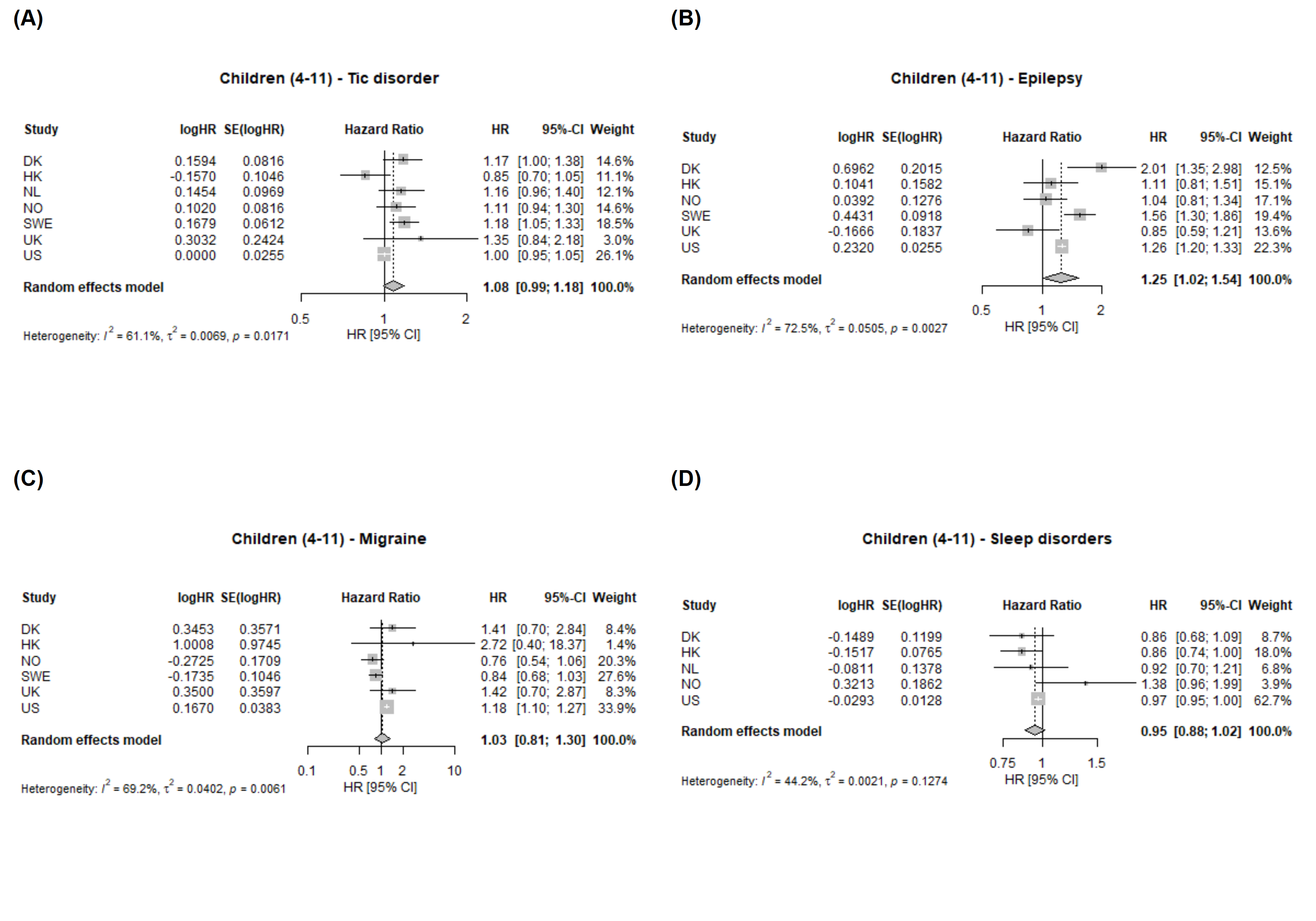


## Figure S13: Forest plots for Children – Psychiatric multimorbidity; Children – Psychiatric inpatient hospitalization; Children – Suicide attempt/intentional self harm; Children – Antidepressants anxiolytics


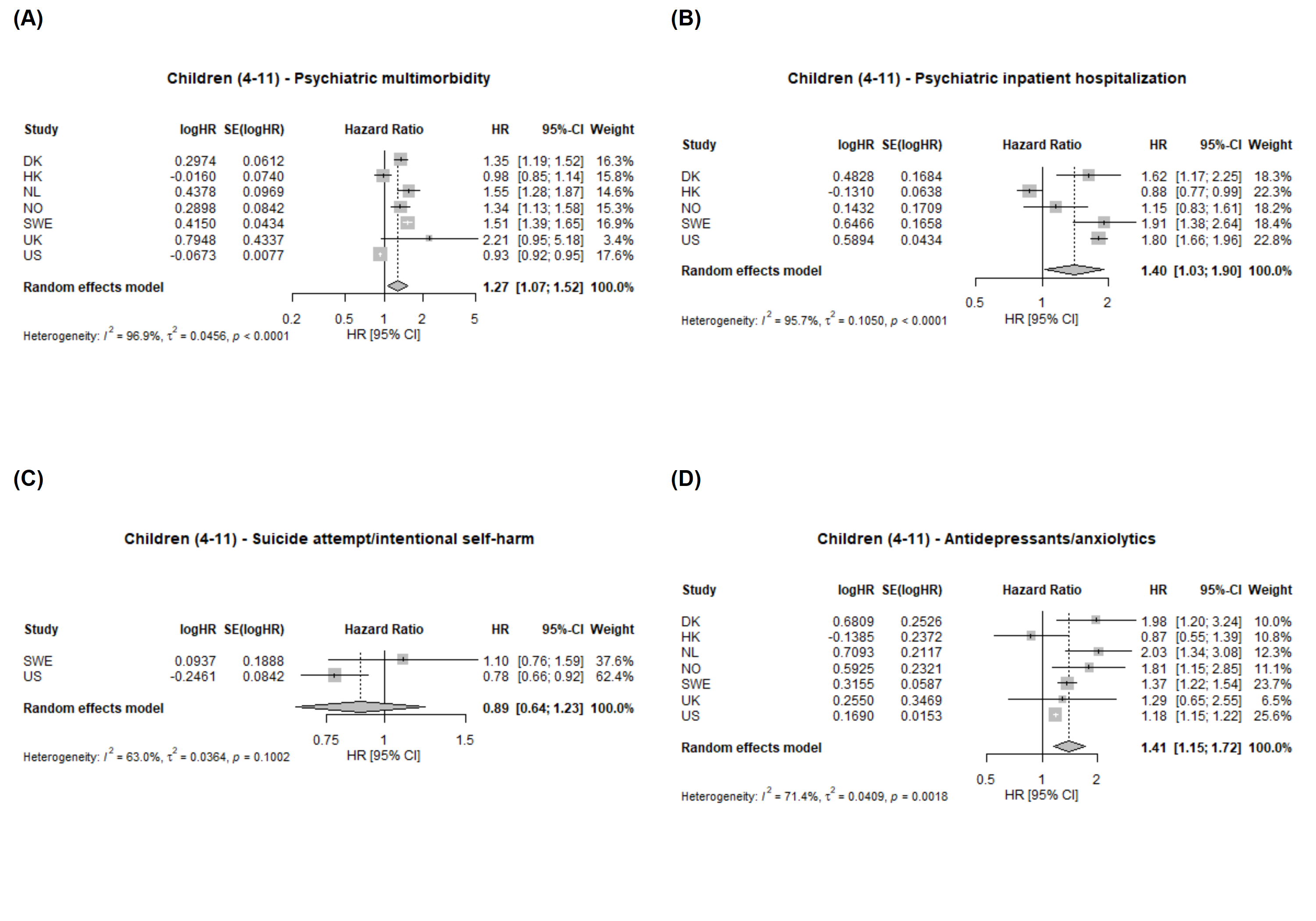


## Figure S14: Forest plots for Children – Antipsychotics; Children – Psychotropic medication; Adolescents – Substance use disorders; Adolescents – Schizophrenia spectrum disorders


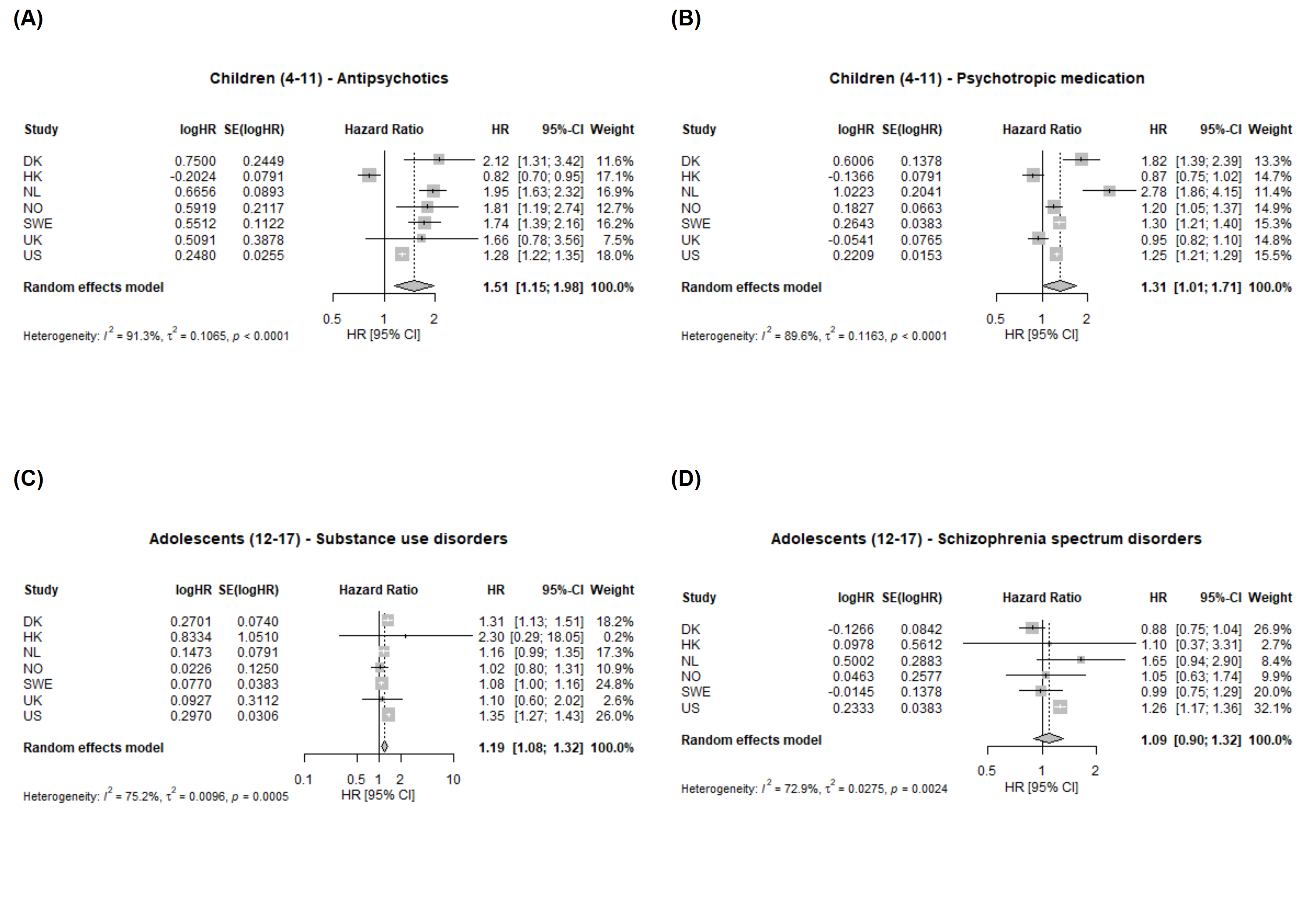


## Figure S15: Forest plots for Adolescents – Bipolar disorder; Adolescents – Major depression; Adolescents – Neurotic, stress related, and somatoform disorders; Adolescents – Anxiety disorders


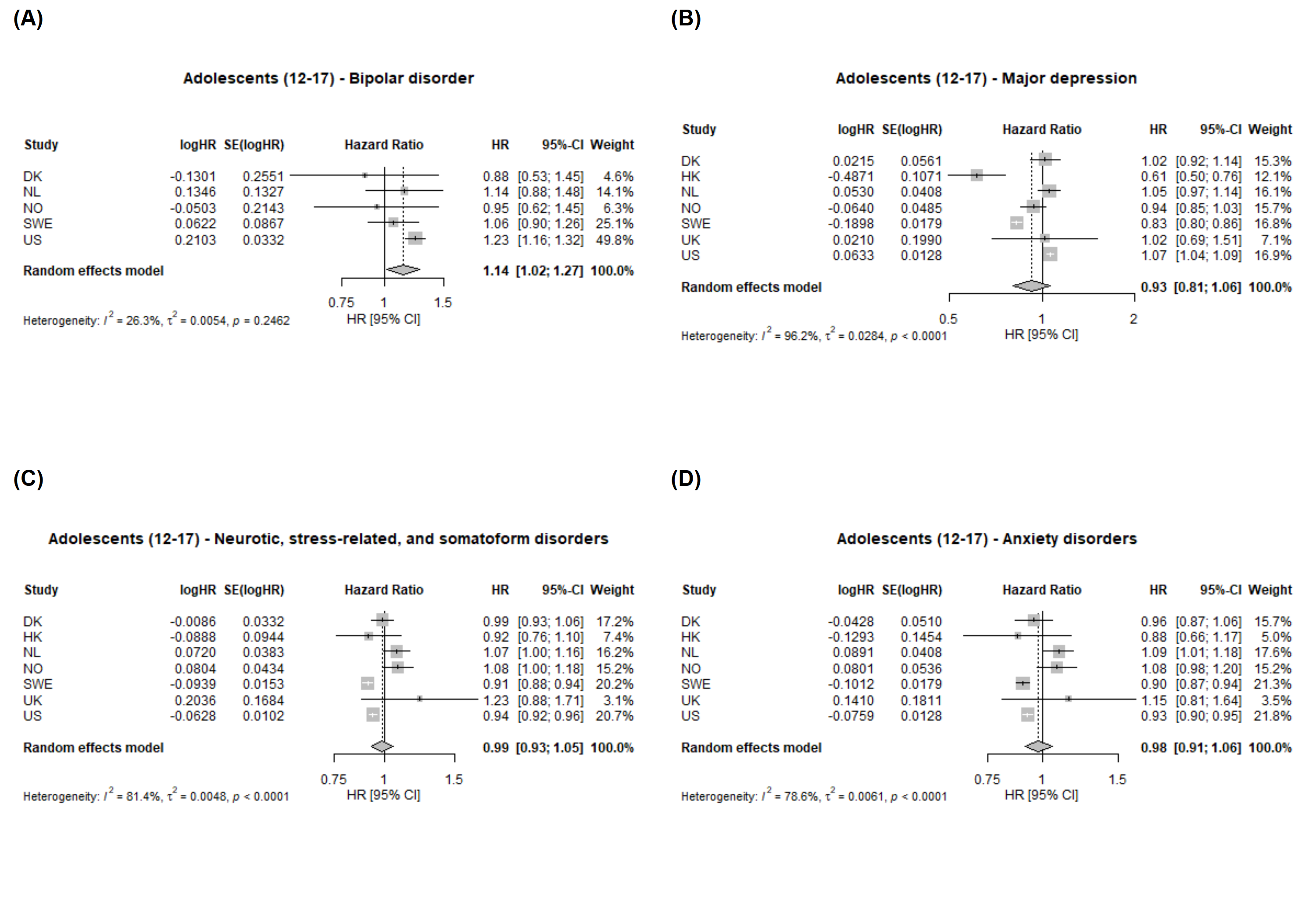


## Figure S16: Forest plots for Adolescents – Obsessive compulsive disorder; Adolescents – Eating disorders; Adolescents – Personality disorders; Adolescents – Borderline personality disorder


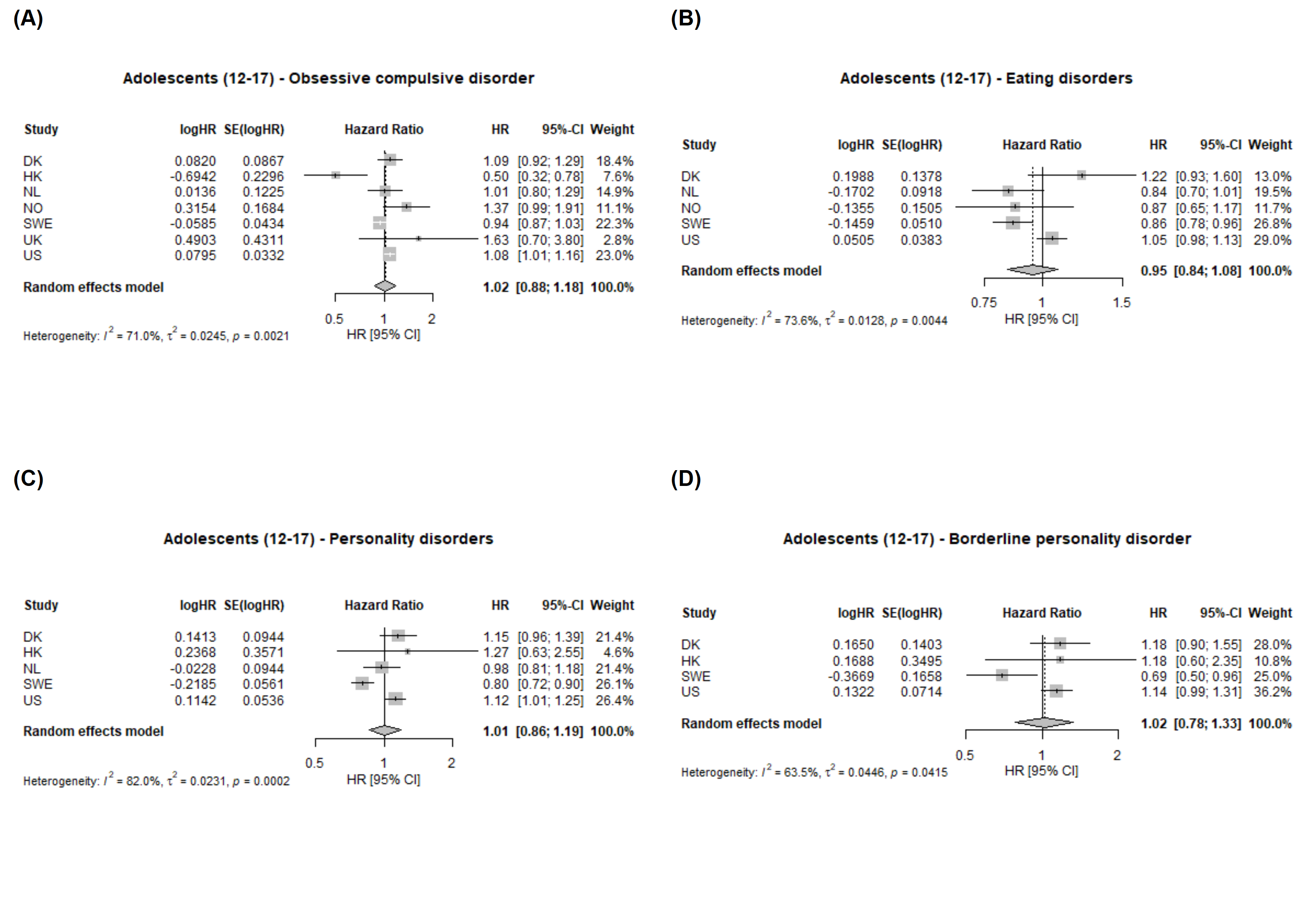


## Figure S17: Forest plots for Adolescents – Intellectual disability; Adolescents – Autism spectrum disorder; Adolescents – Conduct disorders; Adolescents – Tic disorder


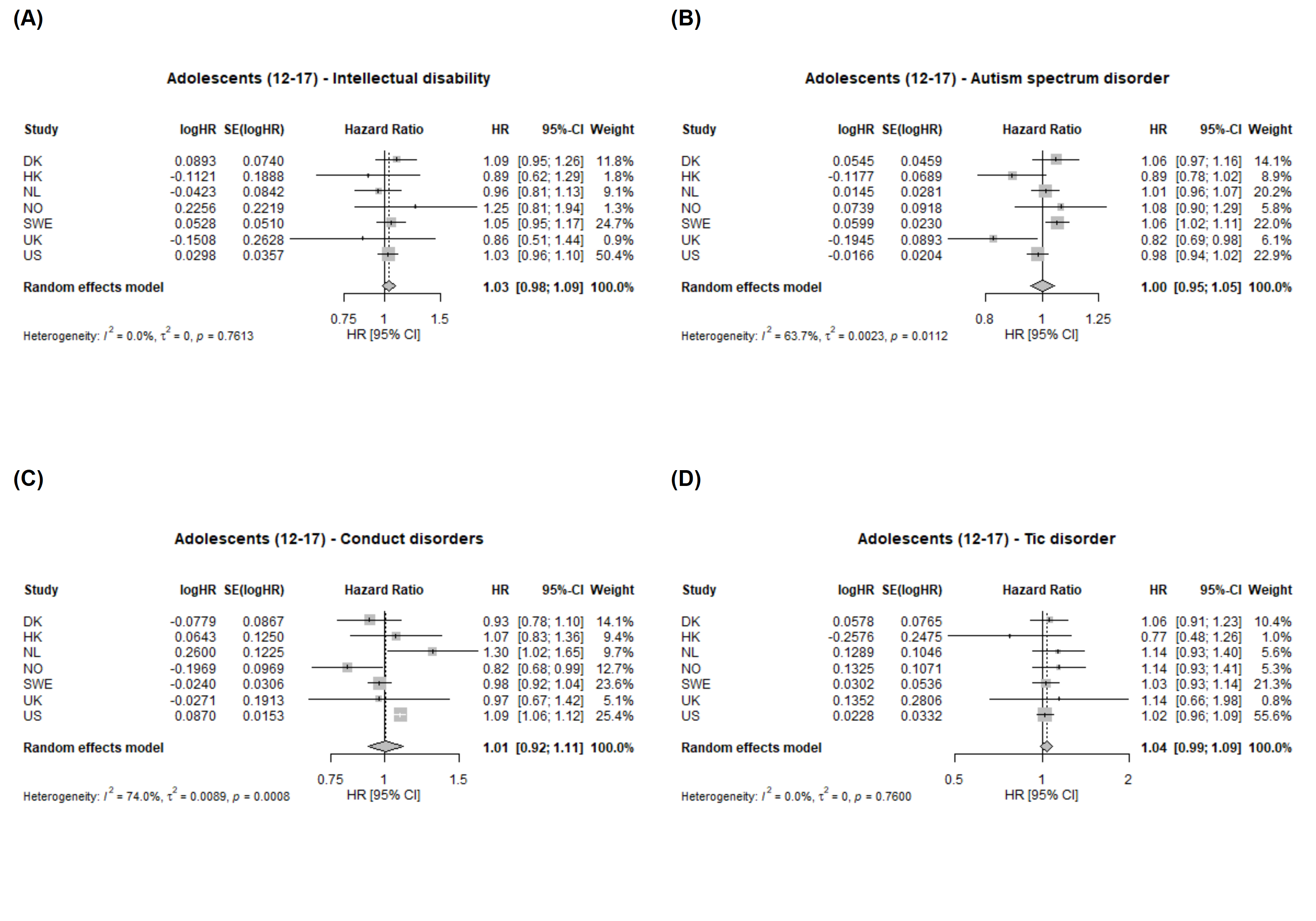


## Figure S18: Forest plots for Adolescents – Epilepsy; Adolescents – Migraine; Adolescents – Sleep disorders; Adolescents – Psychiatric multimorbidity


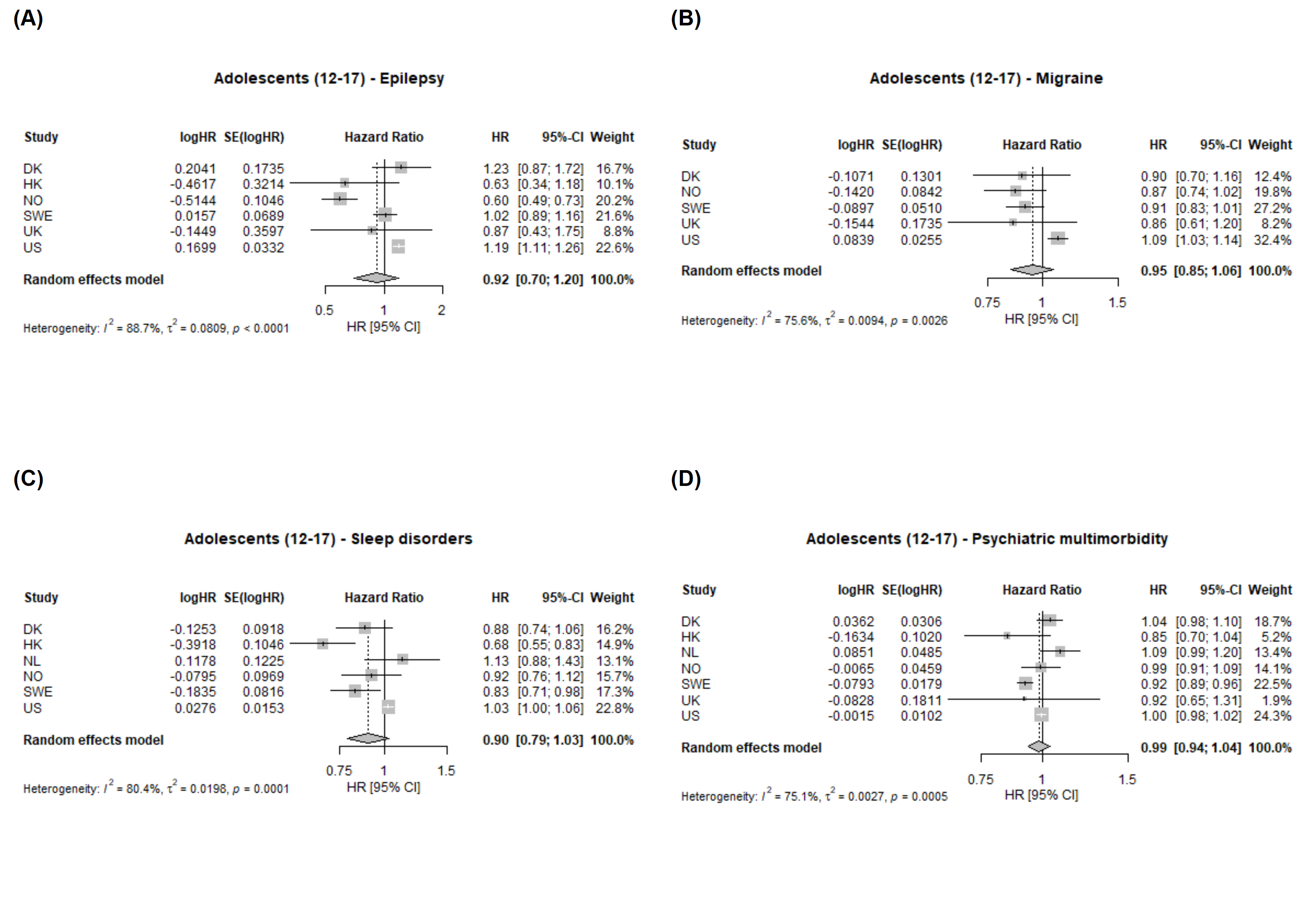


## Figure S19: Forest plots for Adolescents – Psychiatric inpatient hospitalization; Adolescents – Suicide attempt intentional self harm; Adolescents – Antidepressants anxiolytics; Adolescents – Antipsychotics


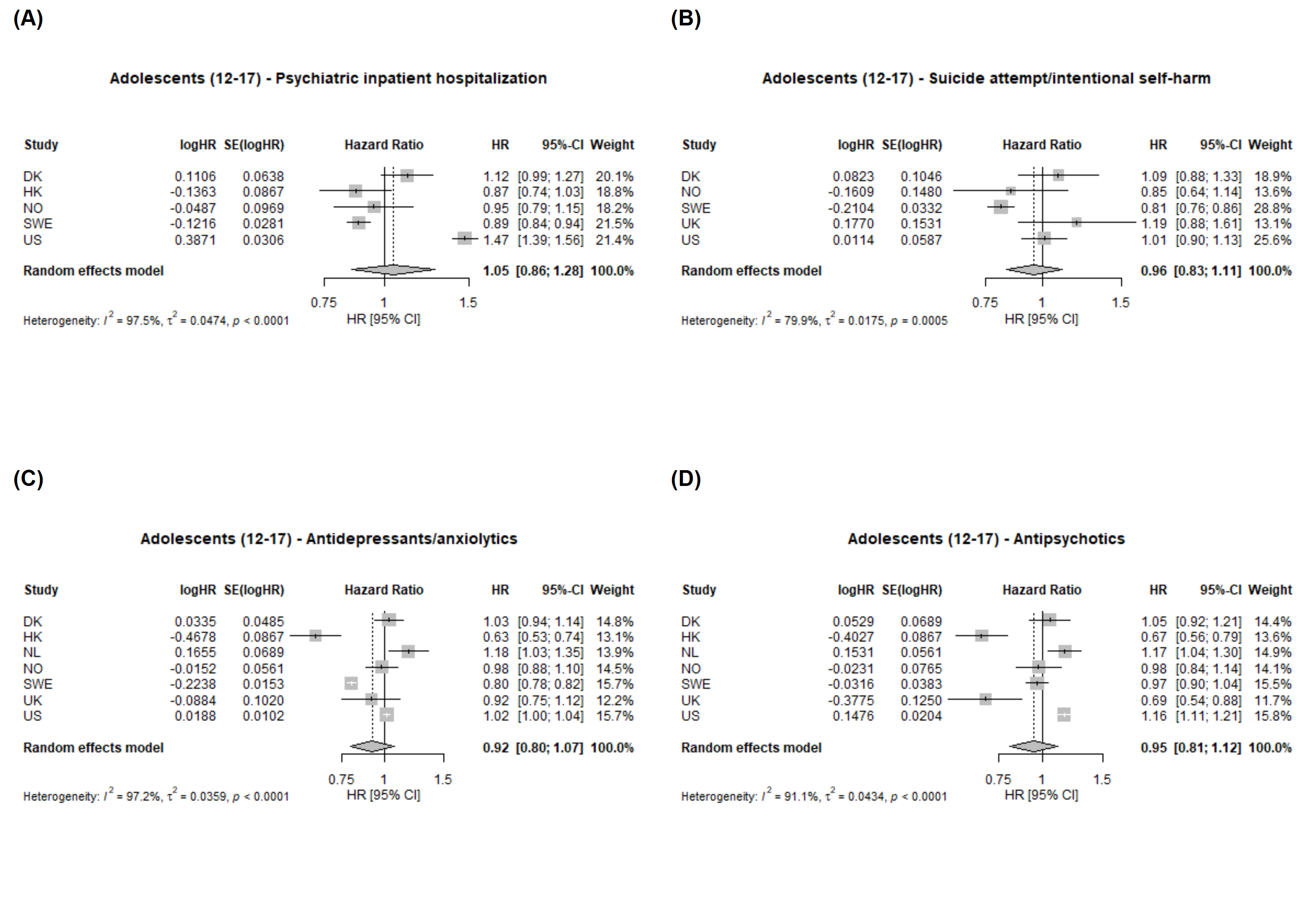


## Figure S20: Forest plots for Adolescents – Psychotropic medication; Young adults – Substance use disorders; Young adults – Schizophrenia spectrum disorders; Young adults – Bipolar disorder


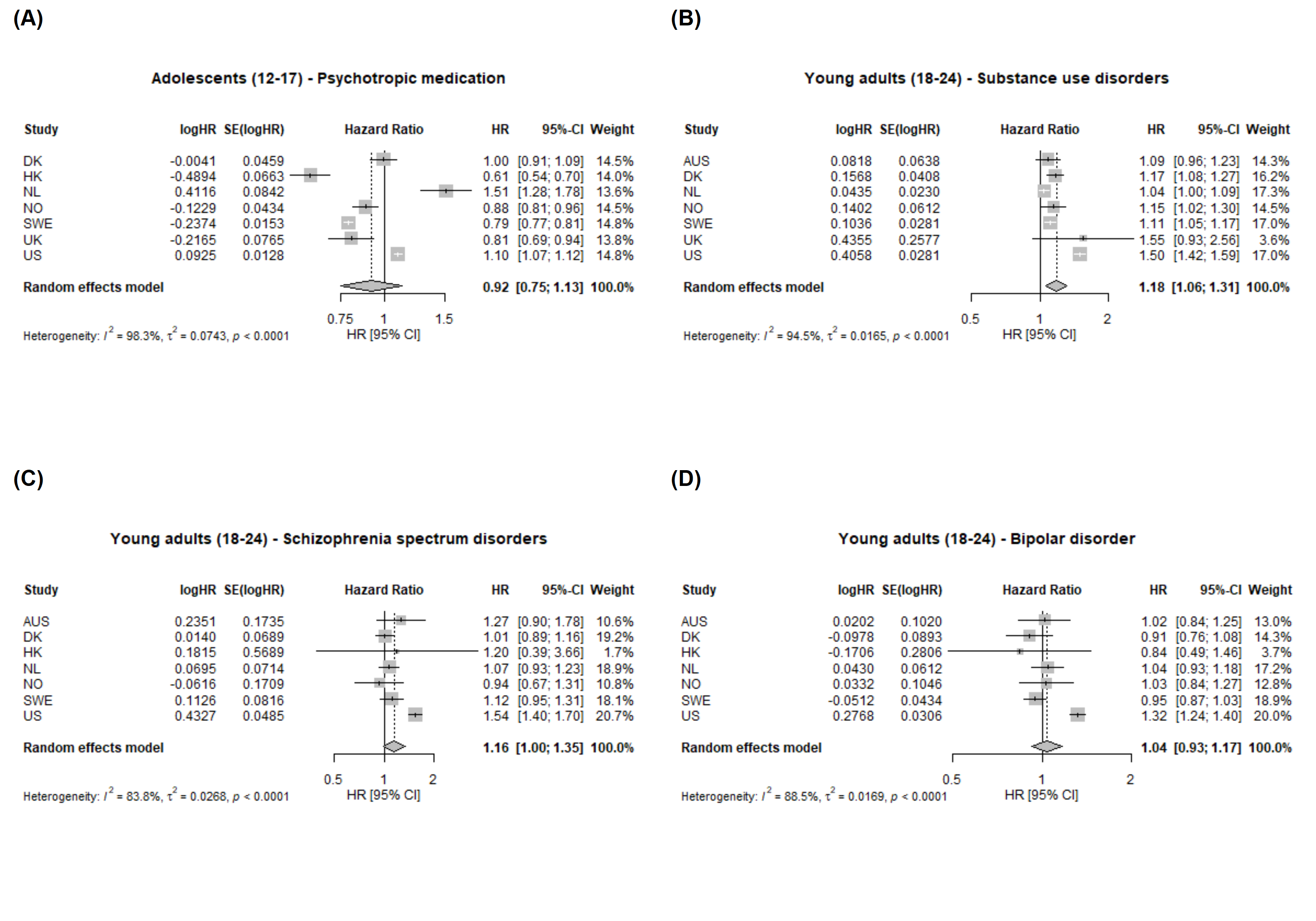


## Figure S21: Forest plots for Young adults – Major depression; Young adults – Neurotic, stress related, and somatoform disorders; Young adults – Anxiety disorders; Young adults – Obsessive compulsive disorder


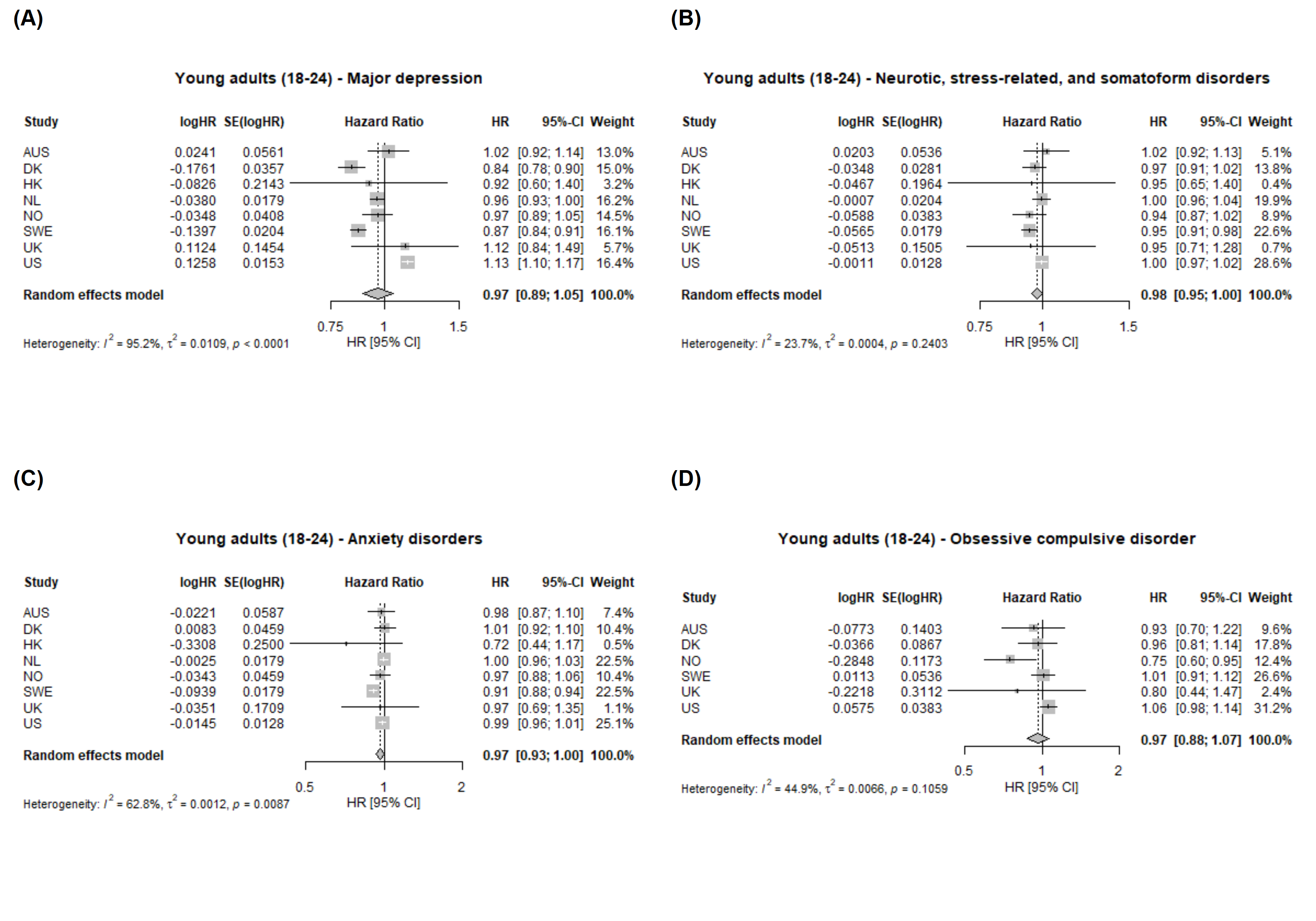


## Figure S22: Forest plots for Young adults – Eating disorders; Young adults – Personality disorders; Young adults – Borderline personality disorder; Young adults – Intellectual disability


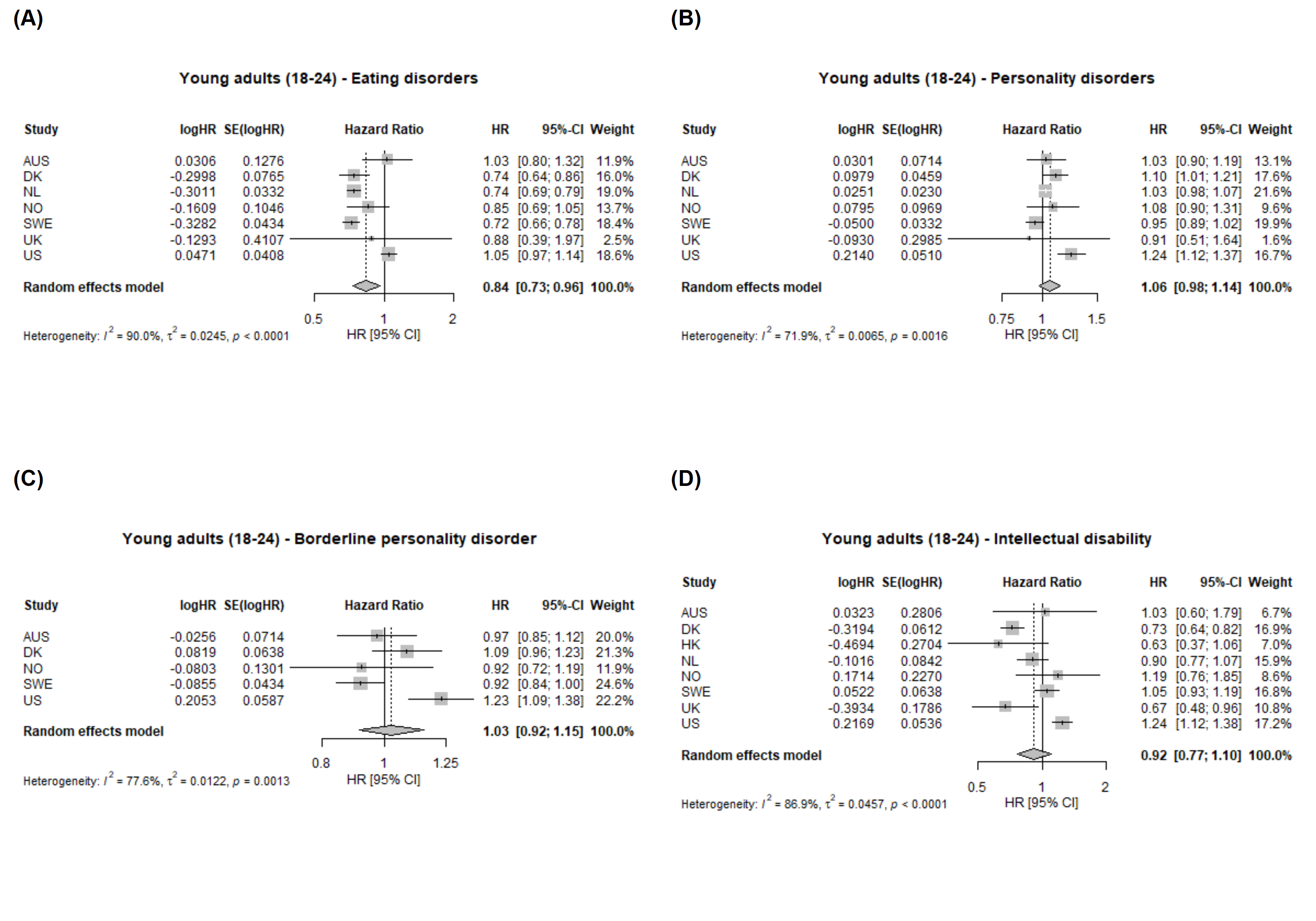


## Figure S23: Forest plots for Young adults – Autism spectrum disorder; Young adults – Conduct disorders; Young adults – Tic disorder; Young adults – Epilepsy


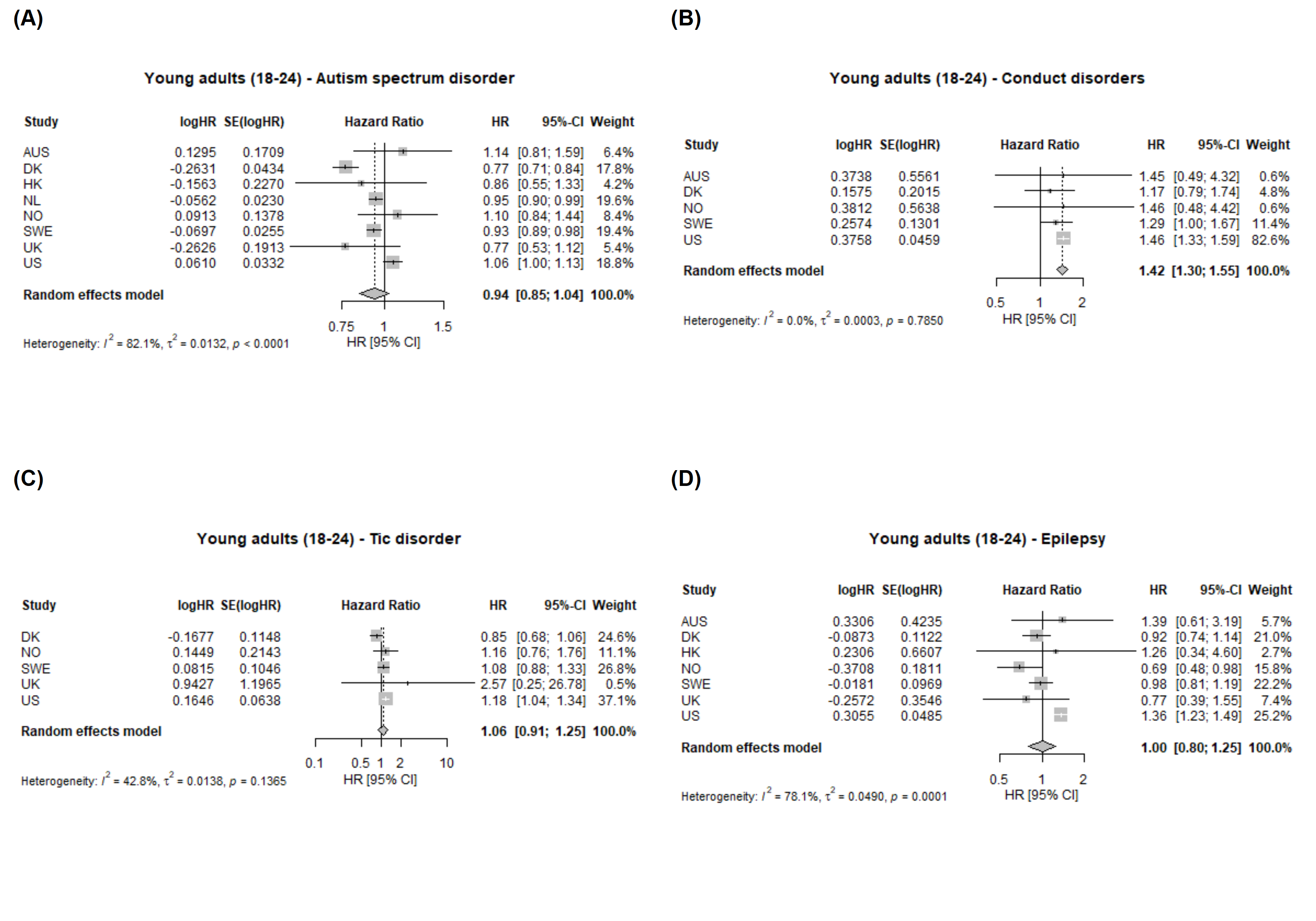


## Figure S24: Forest plots for Young adults – Migraine; Young adults – Sleep disorders; Young adults – Psychiatric multimorbidity; Young adults – Psychiatric inpatient hospitalization


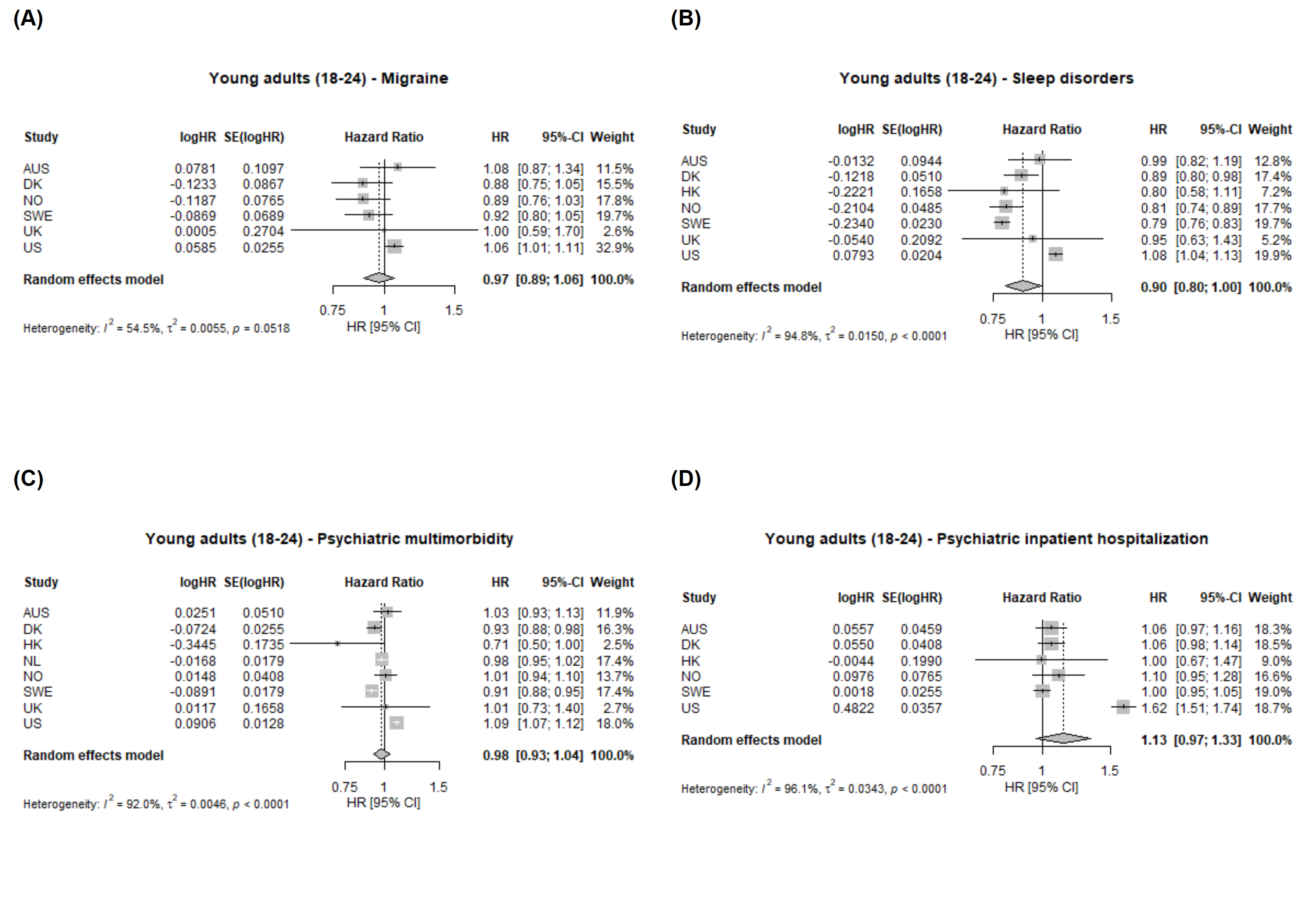


## Figure S25: Forest plots for Young adults – Suicide attempt intentional self harm; Young adults – Antidepressants anxiolytics; Young adults – Antipsychotics; Young adults – Psychotropic medication


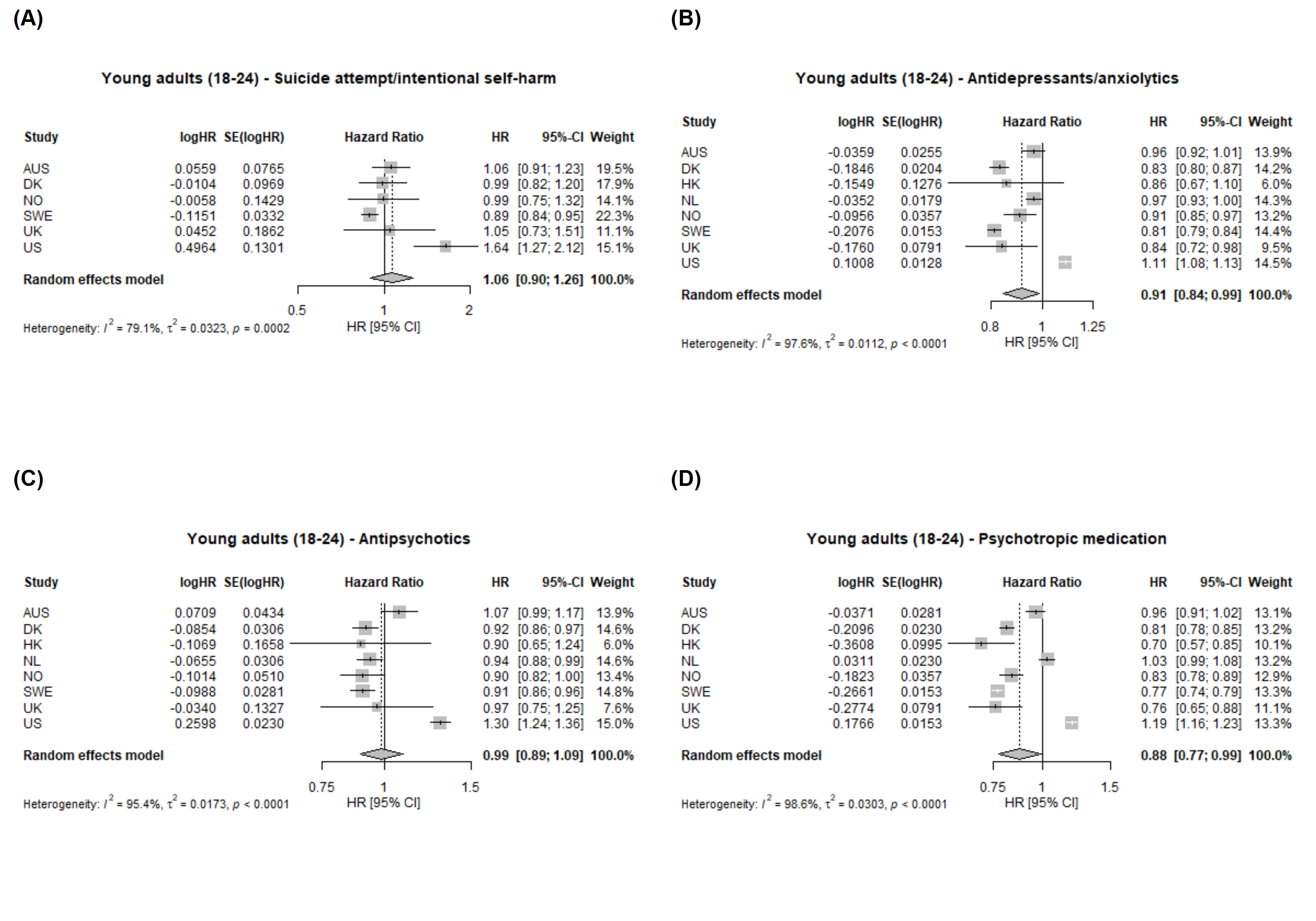


## Figure S26: Forest plots for Adults – Substance use disorders; Adults – Schizophrenia spectrum disorders; Adults – Bipolar disorder; Adults – Major depression


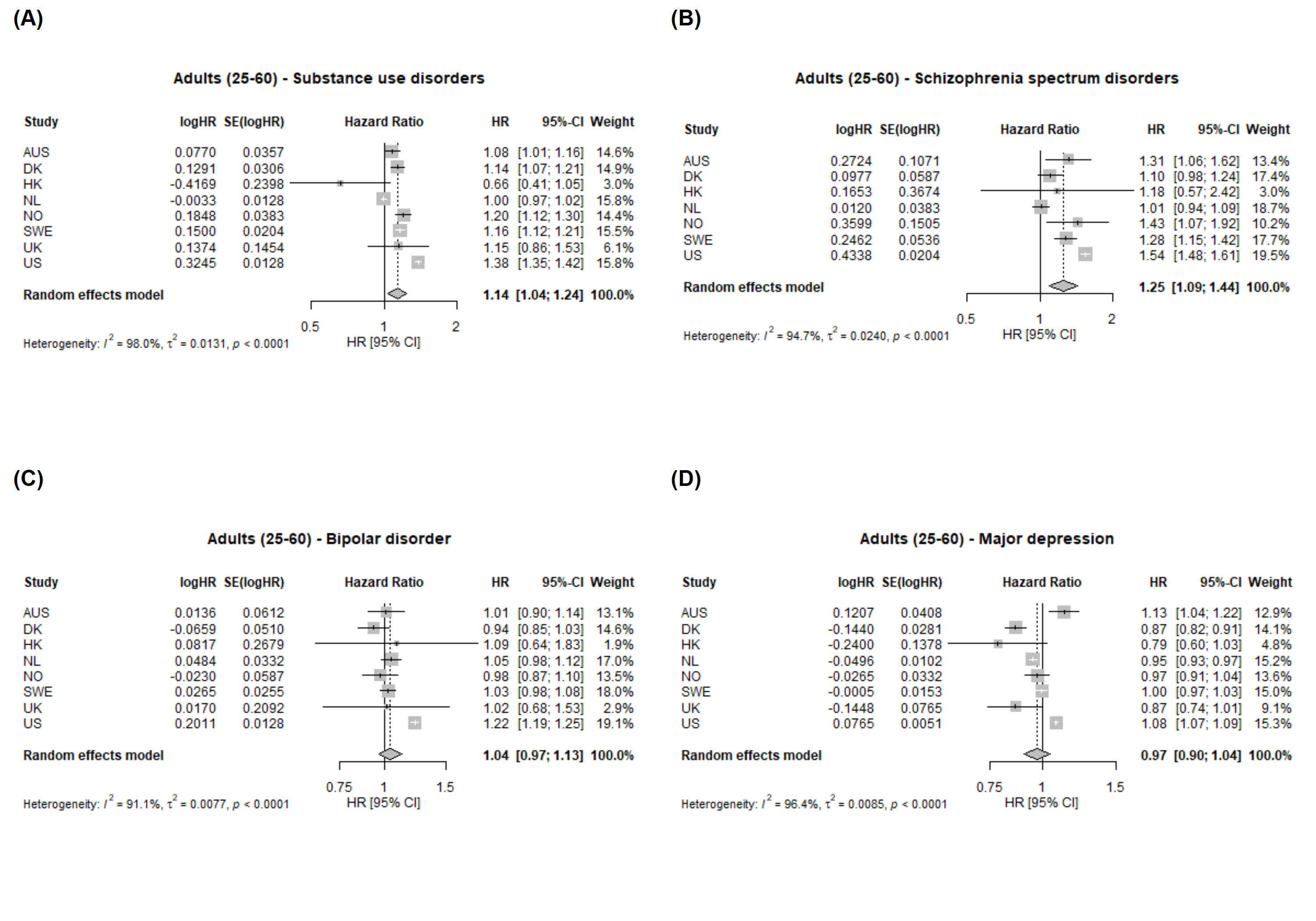


## Figure S27: Forest plots for Adults – Neurotic, stress related, and somatoform disorders; Adults – Anxiety disorders; Adults – Obsessive compulsive disorder; Adults – Eating disorders


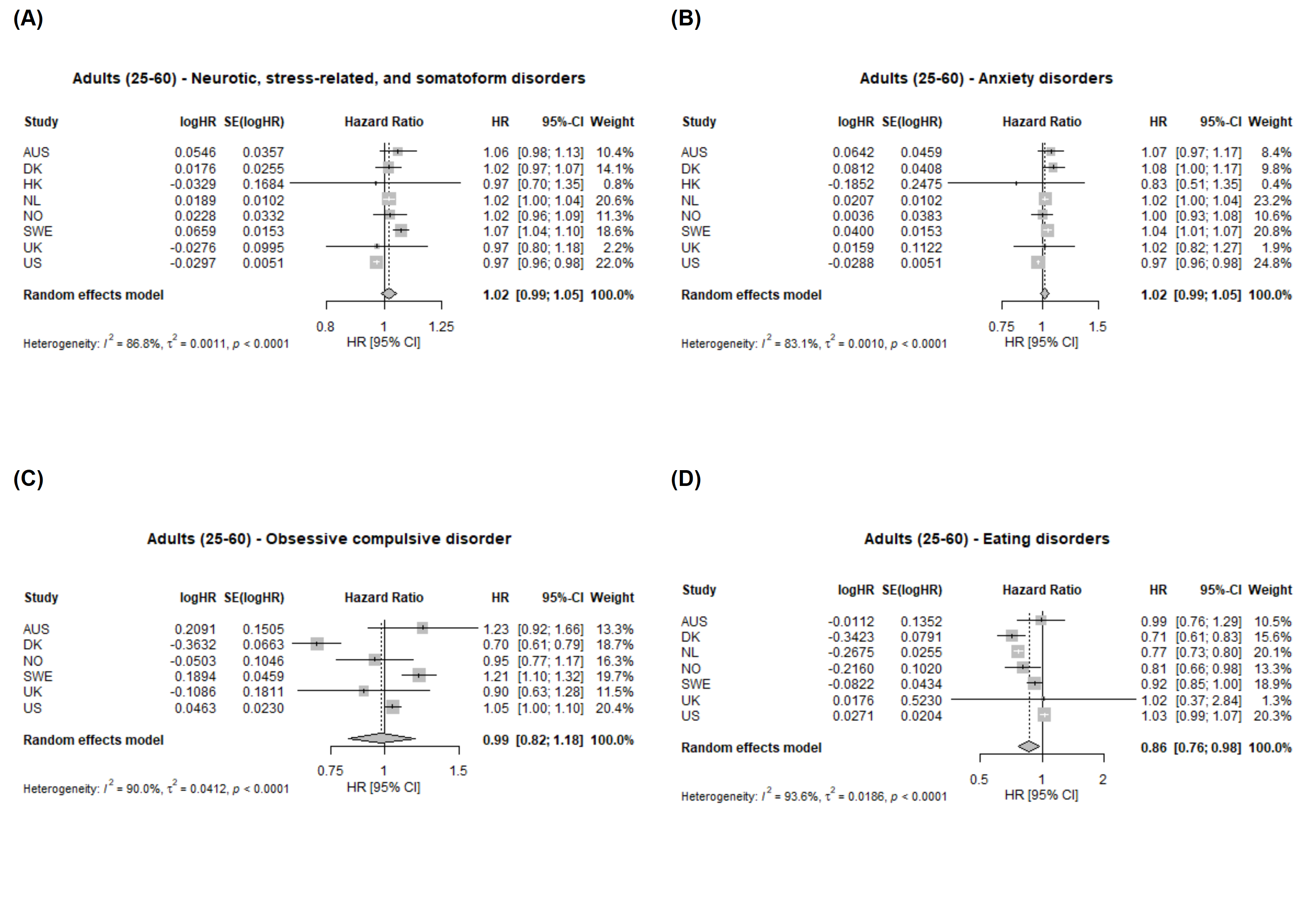


## Figure S28: Forest plots for Adults – Personality disorders; Adults – Borderline personality disorder; Adults – Intellectual disability; Adults – Autism spectrum disorder


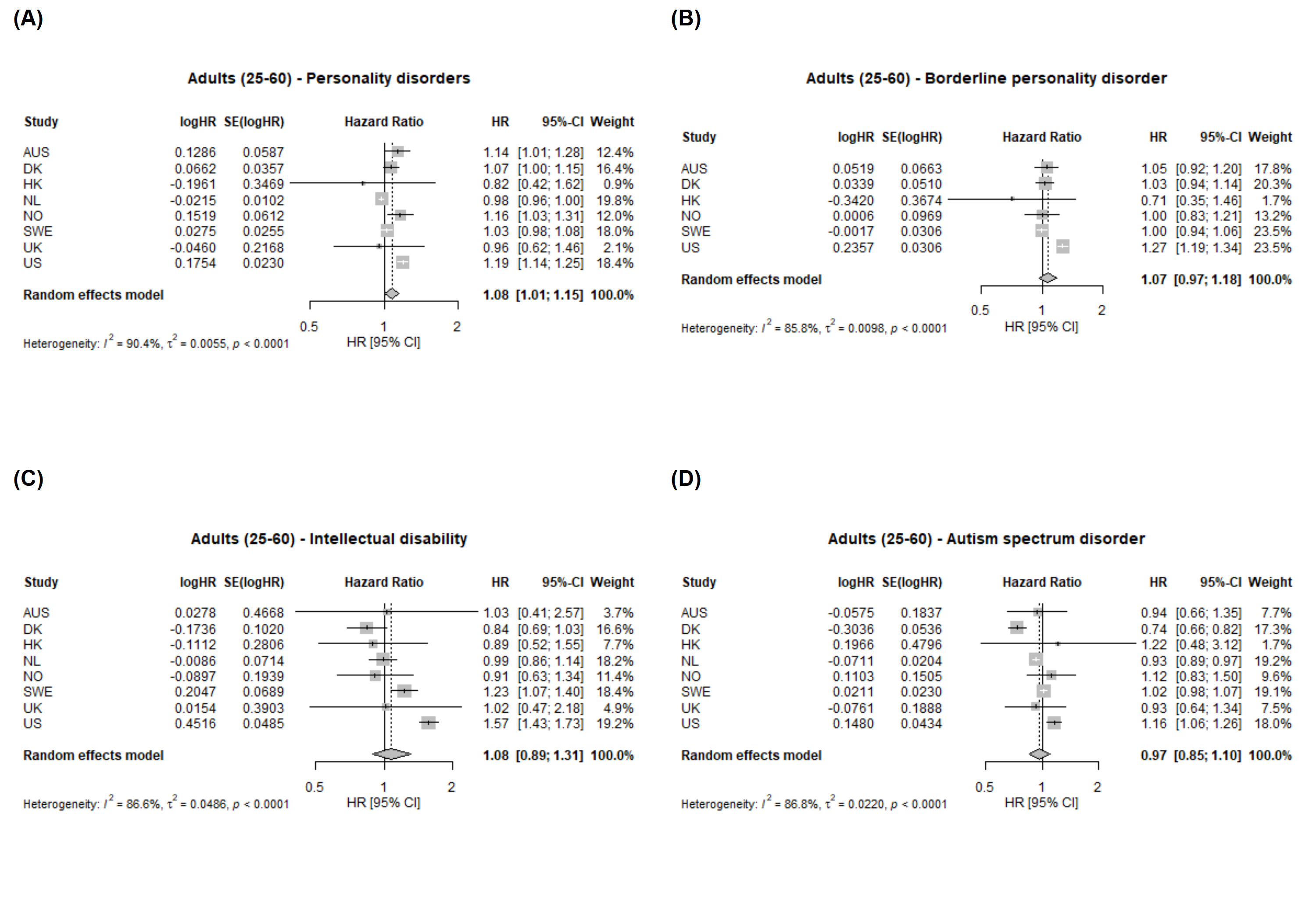


## Figure S29: Forest plots for Adults – Conduct disorders; Adults – Tic disorder; Adults – Epilepsy; Adults – Migraine


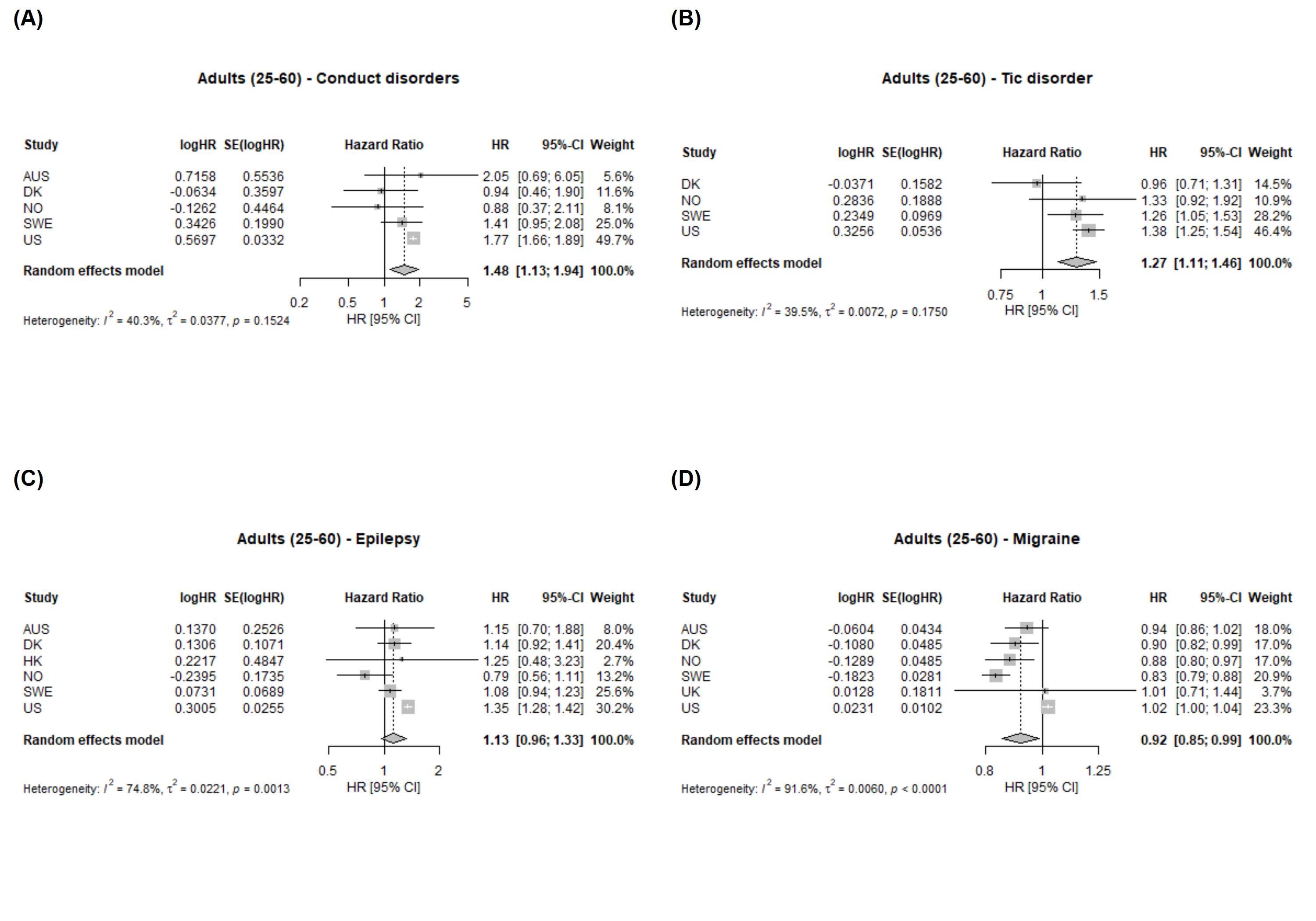


## Figure S30: Forest plots for Adults – Sleep disorders; Adults – Psychiatric multimorbidity; Adults – Psychiatric inpatient hospitalisation; Adults – Suicide attempt intentional self harm


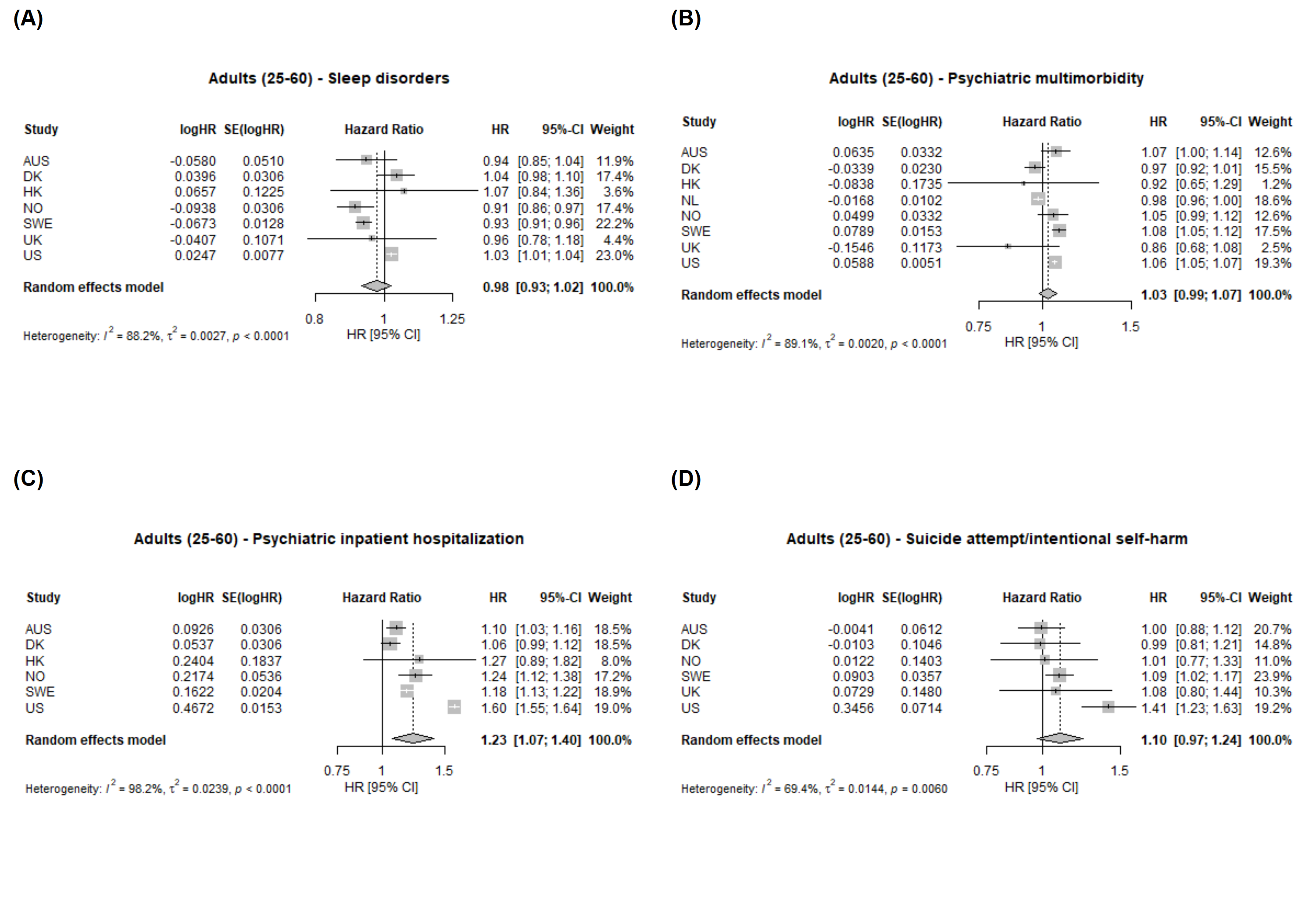


## Figure S31: Forest plots for Adults – Antidepressants anxiolytics; Adults – Antipsychotics; Adults – Psychotropic medication


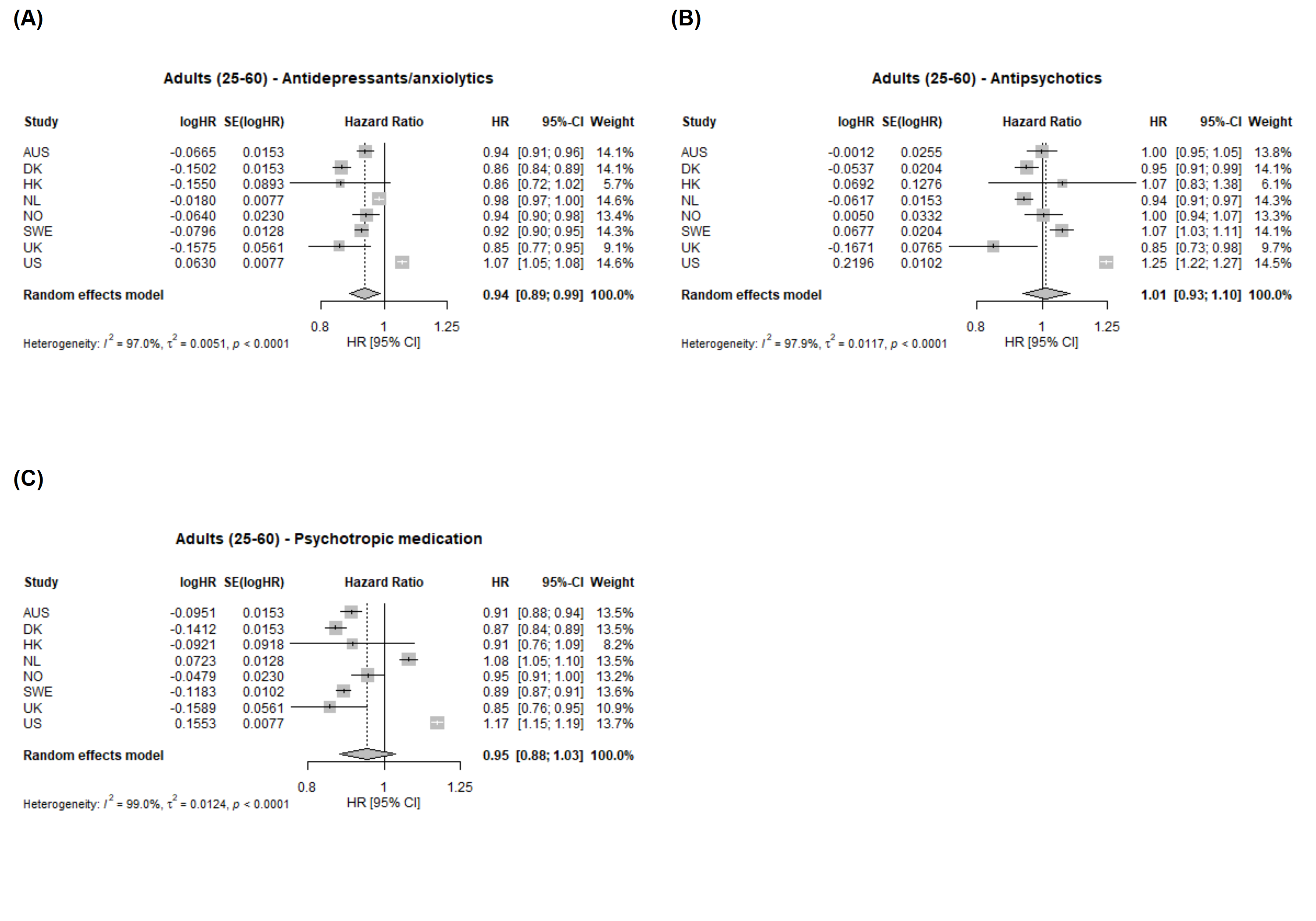


## **Figure S32.** Meta-analytic associations of psychiatric and neurological indicators with ADHD medication discontinuation, excluding US data


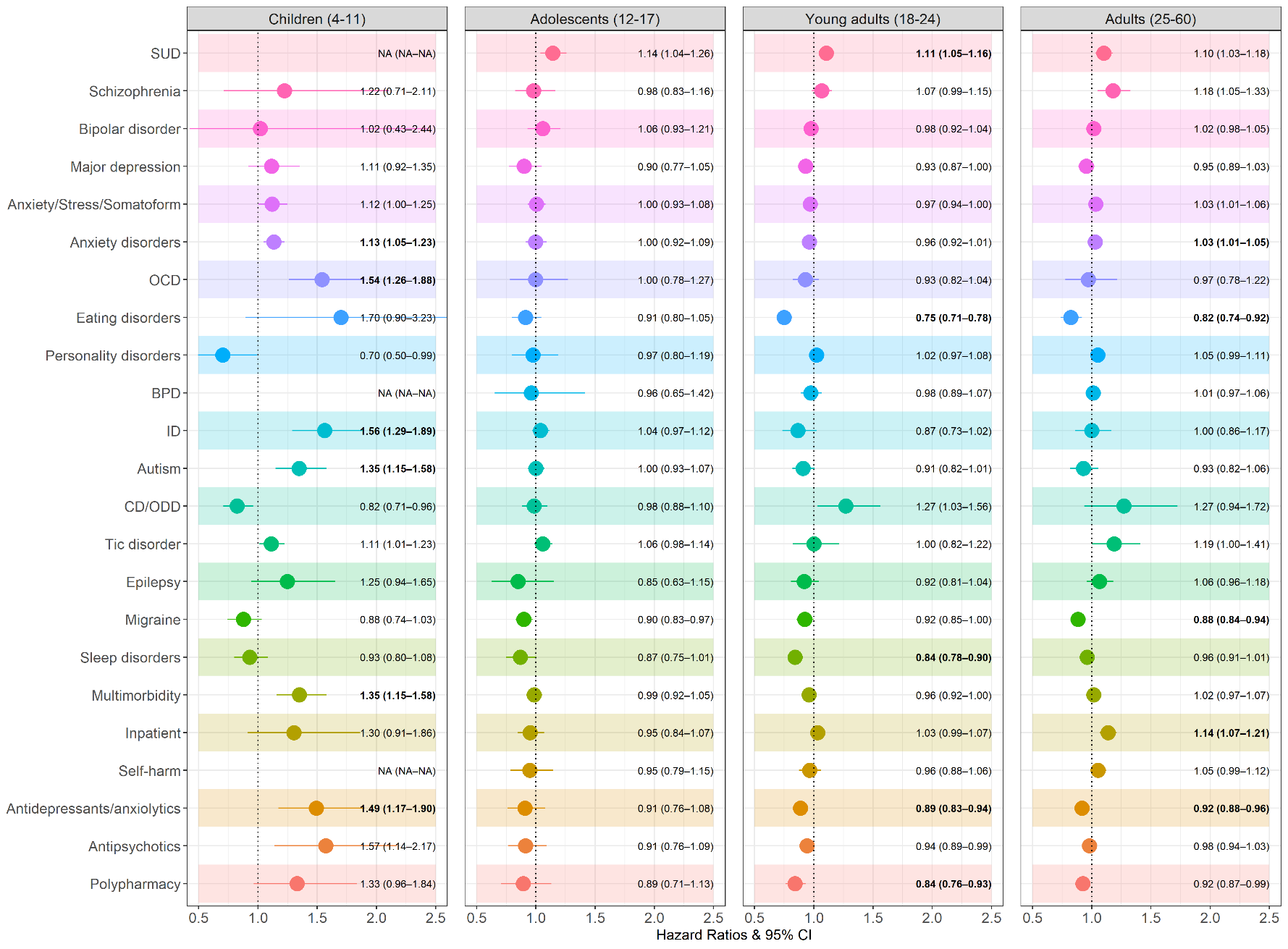


**Note:** Pooled estimates from secondary meta-analyses excluding data from the US. Estimates surviving false discovery rate correction are shown in bold. Abbreviations: SUD, substance use disorder; OCD, obsessive compulsive disorders; BPD, borderline personality disorder; ID, intellectual disability; CD/ODD, conduct disorder/oppositional defiant disorder; Inpatient, Psychiatric inpatient admission; Polypharmacy, psychotropic polypharmacy.

## Figure S33. Multivariable country specific associations of psychiatric and neurological indicators with ADHD medication discontinuation (Australia, Denmark, Hong Kong, the Netherlands)


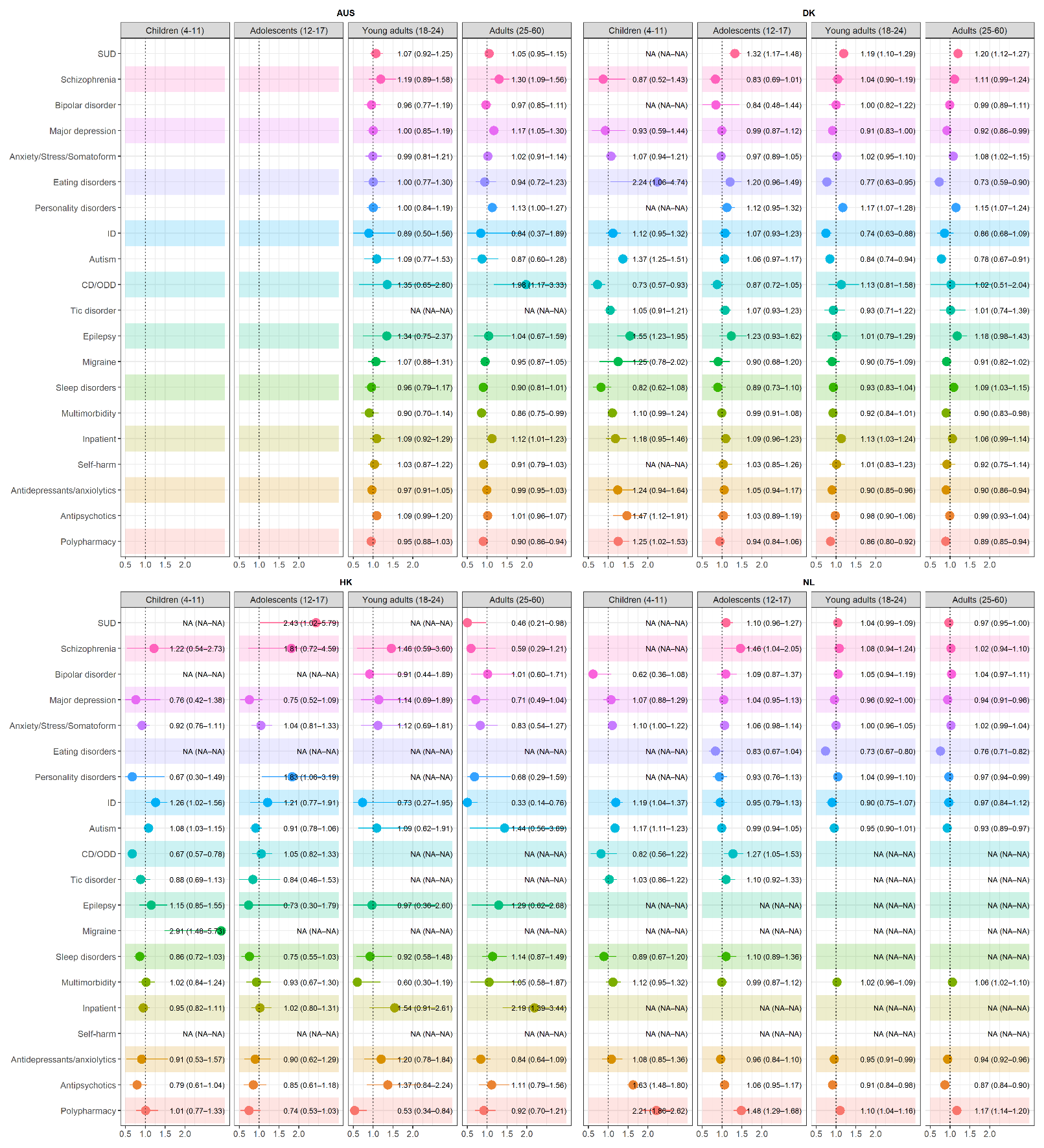


**Note**: Associations shown from a multivariable model including all indicators in the single model, in each country. All indicators were defined in the 2 years prior to ADHD medication initiation, except psychotropic polypharmacy (within 3 months prior to initiation). **Abbreviations**: SUD, substance use disorder; OCD, obsessive compulsive disorders; BPD, borderline personality disorder; ID, intellectual disability; CD/ODD, conduct disorder/oppositional defiant disorder; Inpatient, psychiatric inpatient admission; Polypharmacy, psychotropic polypharmacy

## Figure S34. Multivariable country specific associations of psychiatric and neurological indicators with ADHD medication discontinuation (Norway, Sweden, the United Kingdom, the United States)


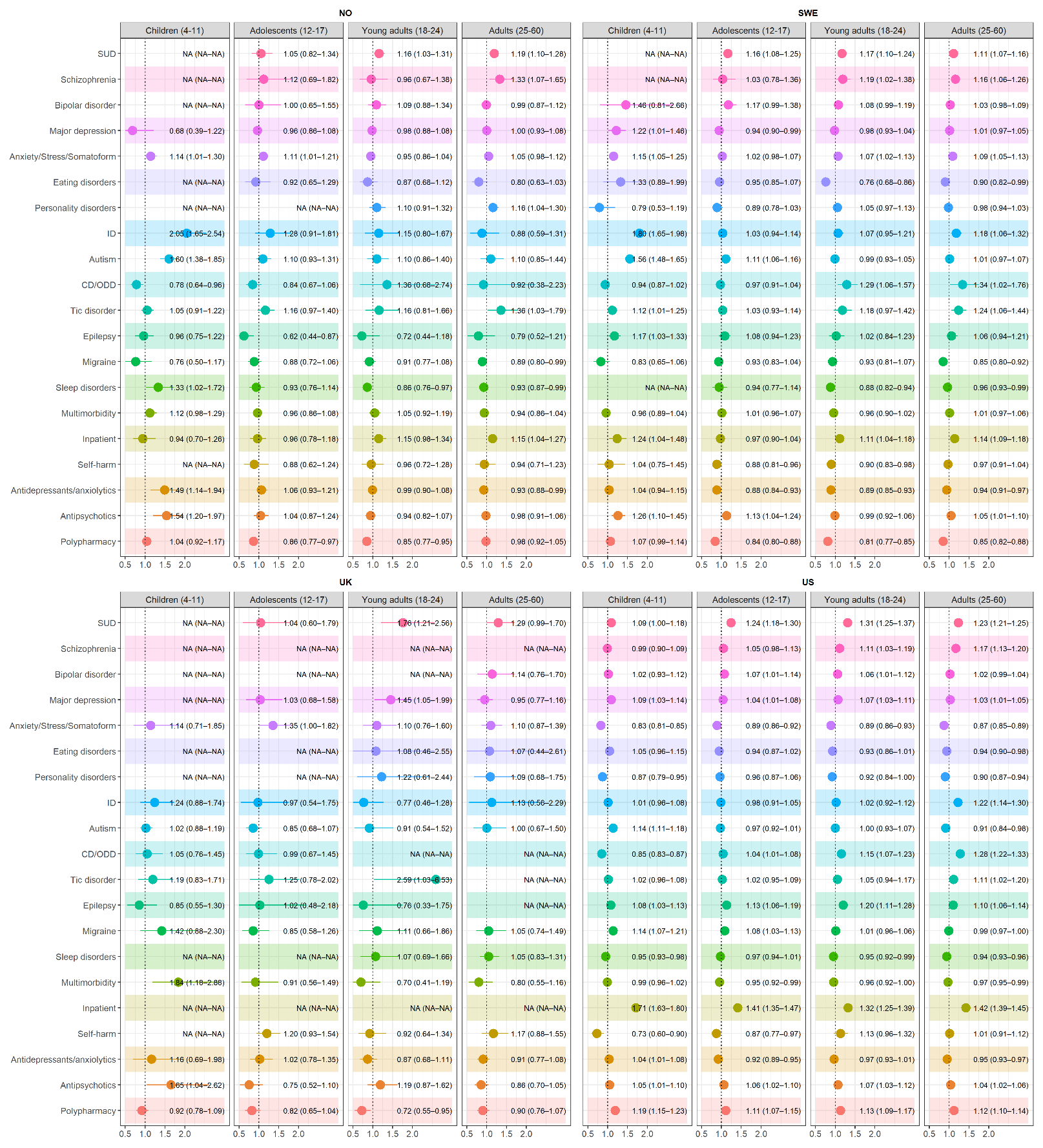


**Note**: Associations shown from a multivariable model including all indicators in the single model, in each country. All indicators were defined in the 2 years prior to ADHD medication initiation, except psychotropic polypharmacy (within 3 months prior to initiation). **Abbreviations**: SUD, substance use disorder; OCD, obsessive compulsive disorders; BPD, borderline personality disorder; ID, intellectual disability; CD/ODD, conduct disorder/oppositional defiant disorder; Inpatient, psychiatric inpatient admission; Polypharmacy, psychotropic polypharmacy

## Figure S35. Meta-analytic associations of psychiatric and neurological indicators with ADHD medication discontinuation in males and females


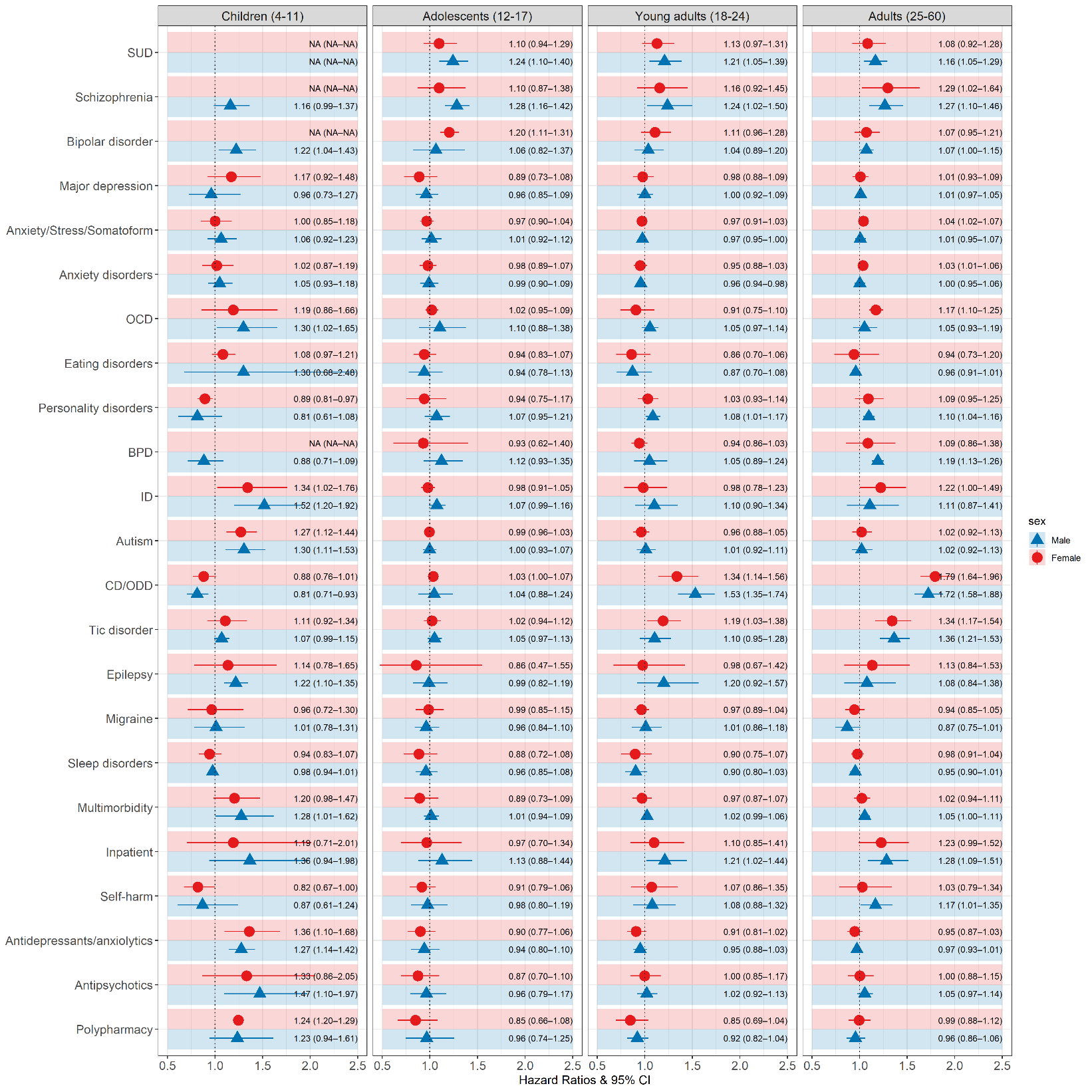


**Abbreviations**: SUD, substance use disorder; OCD, obsessive compulsive disorders; BPD, borderline personality disorder; ID, intellectual disability; CD/ODD, conduct disorder/oppositional defiant disorder; Inpatient, Psychiatric inpatient admission; Polypharmacy, psychotropic polypharmacy

## Figure S36. Sex-stratified associations of psychiatric and neurological indicators with ADHD medication discontinuation in Australia


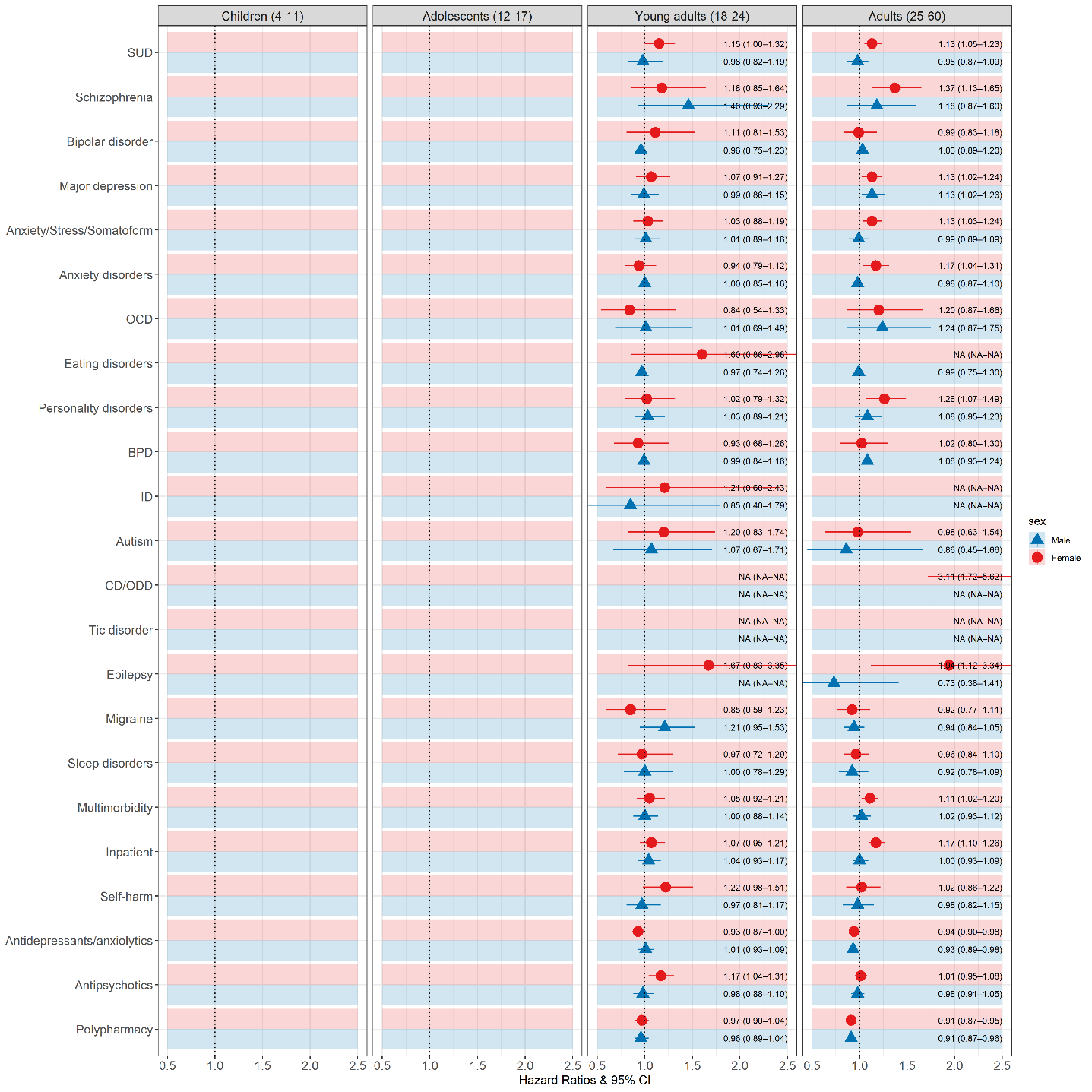


**Abbreviations:** SUD, substance use disorder; OCD, obsessive compulsive disorders; BPD, borderline personality disorder; ID, intellectual disability; CD/ODD, conduct disorder/oppositional defiant disorder; Inpatient, Psychiatric inpatient admission; Polypharmacy, psychotropic polypharmacy

## Figure S37. Sex-stratified associations of psychiatric and neurological indicators with ADHD medication discontinuation in Hong Kong


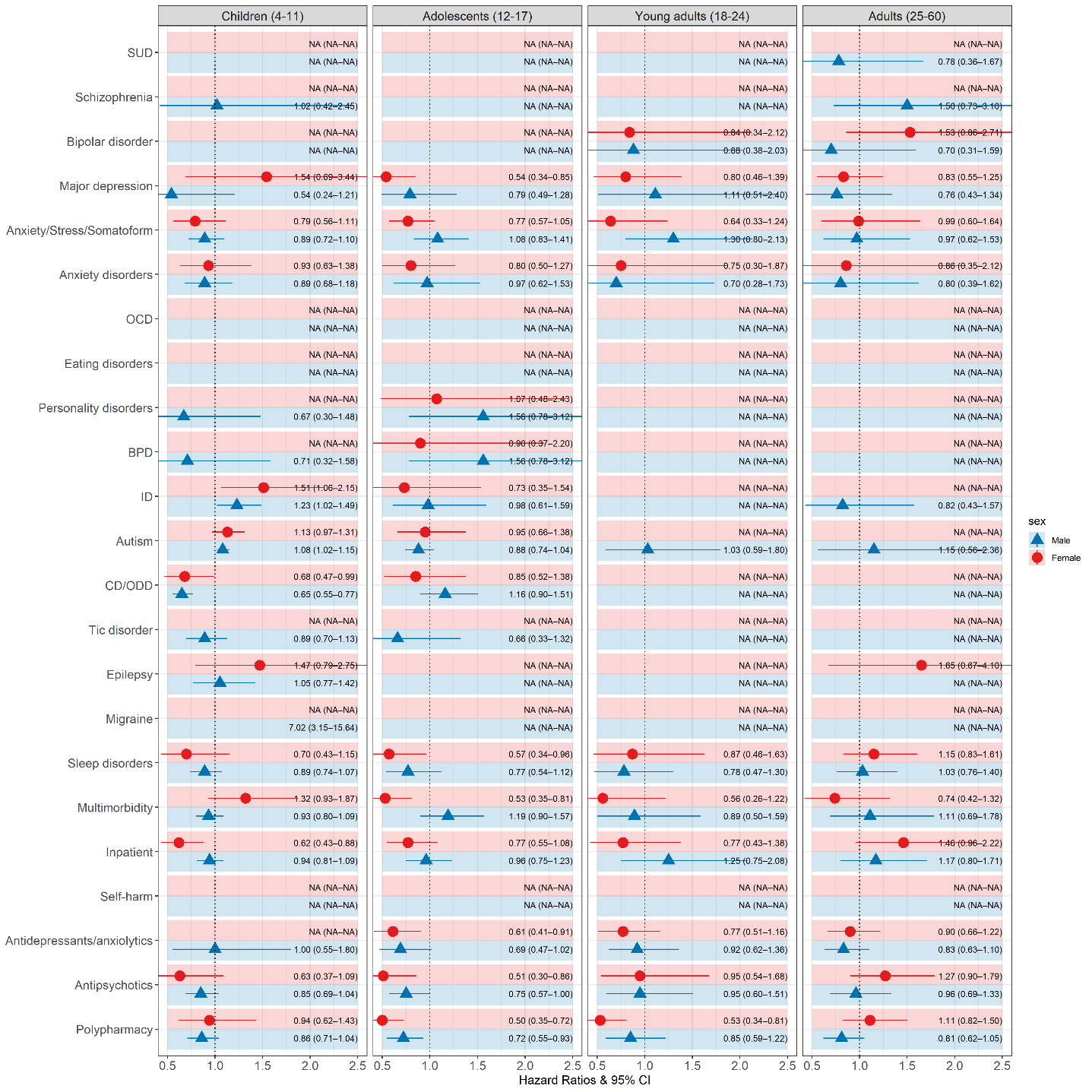


**Abbreviations:** SUD, substance use disorder; OCD, obsessive compulsive disorders; BPD, borderline personality disorder; ID, intellectual disability; CD/ODD, conduct disorder/oppositional defiant disorder; Inpatient, Psychiatric inpatient admission; Polypharmacy, psychotropic polypharmacy

## Figure S38. Sex-stratified associations of psychiatric and neurological indicators with ADHD medication discontinuation in the Netherlands


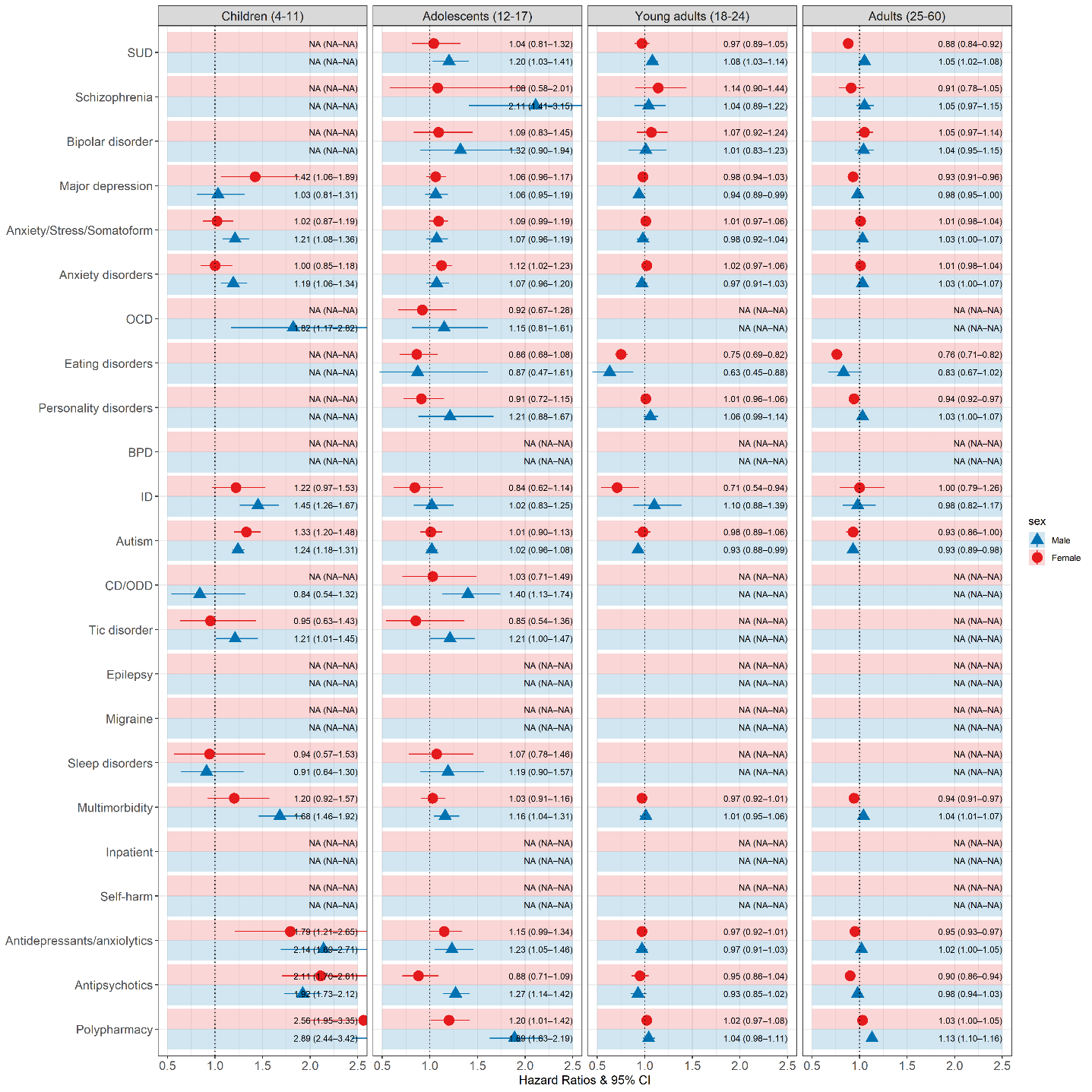


**Abbreviations:** SUD, substance use disorder; OCD, obsessive compulsive disorders; BPD, borderline personality disorder; ID, intellectual disability; CD/ODD, conduct disorder/oppositional defiant disorder; Inpatient, Psychiatric inpatient admission; Polypharmacy, psychotropic polypharmacy

## Figure S39. Sex-stratified associations of psychiatric and neurological indicators with ADHD medication discontinuation in Norway


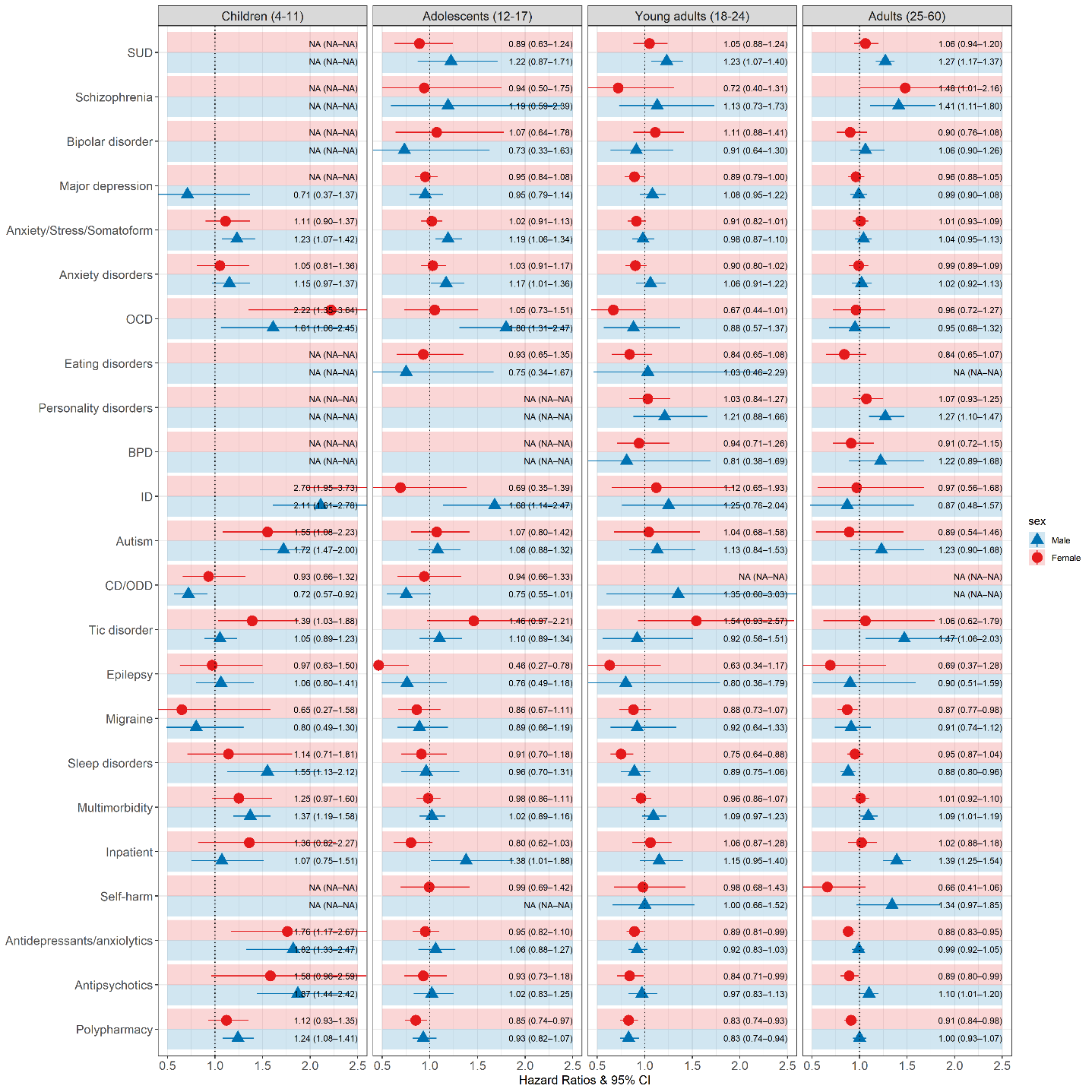


**Abbreviations:** SUD, substance use disorder; OCD, obsessive compulsive disorders; BPD, borderline personality disorder; ID, intellectual disability; CD/ODD, conduct disorder/oppositional defiant disorder; Inpatient, Psychiatric inpatient admission; Polypharmacy, psychotropic polypharmacy

## Figure S40. Sex-stratified associations of psychiatric and neurological indicators with ADHD medication discontinuation in Sweden


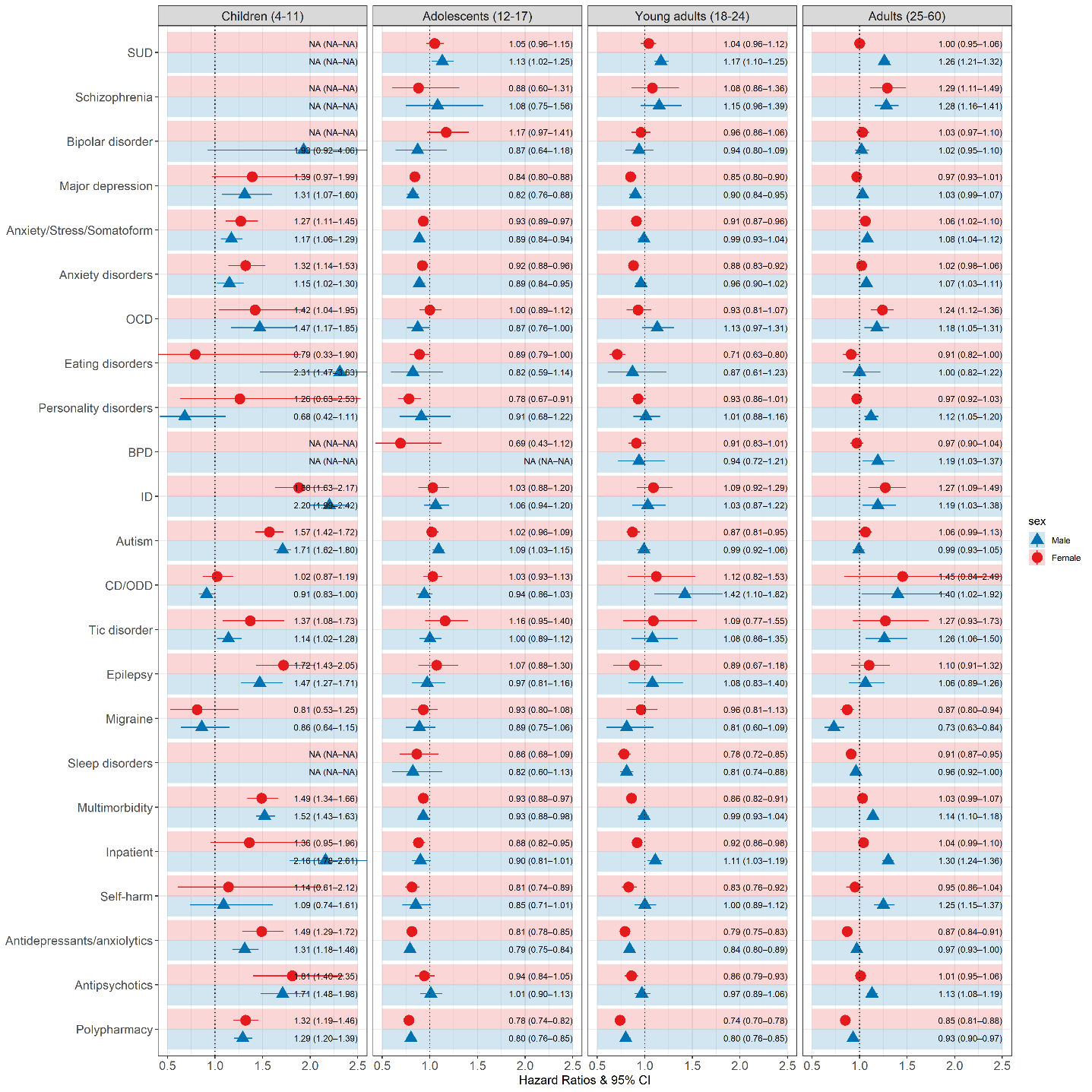


**Abbreviations:** SUD, substance use disorder; OCD, obsessive compulsive disorders; BPD, borderline personality disorder; ID, intellectual disability; CD/ODD, conduct disorder/oppositional defiant disorder; Inpatient, Psychiatric inpatient admission; Polypharmacy, psychotropic polypharmacy

## Figure S41. Sex-stratified associations of psychiatric and neurological indicators with ADHD medication discontinuation in the UK


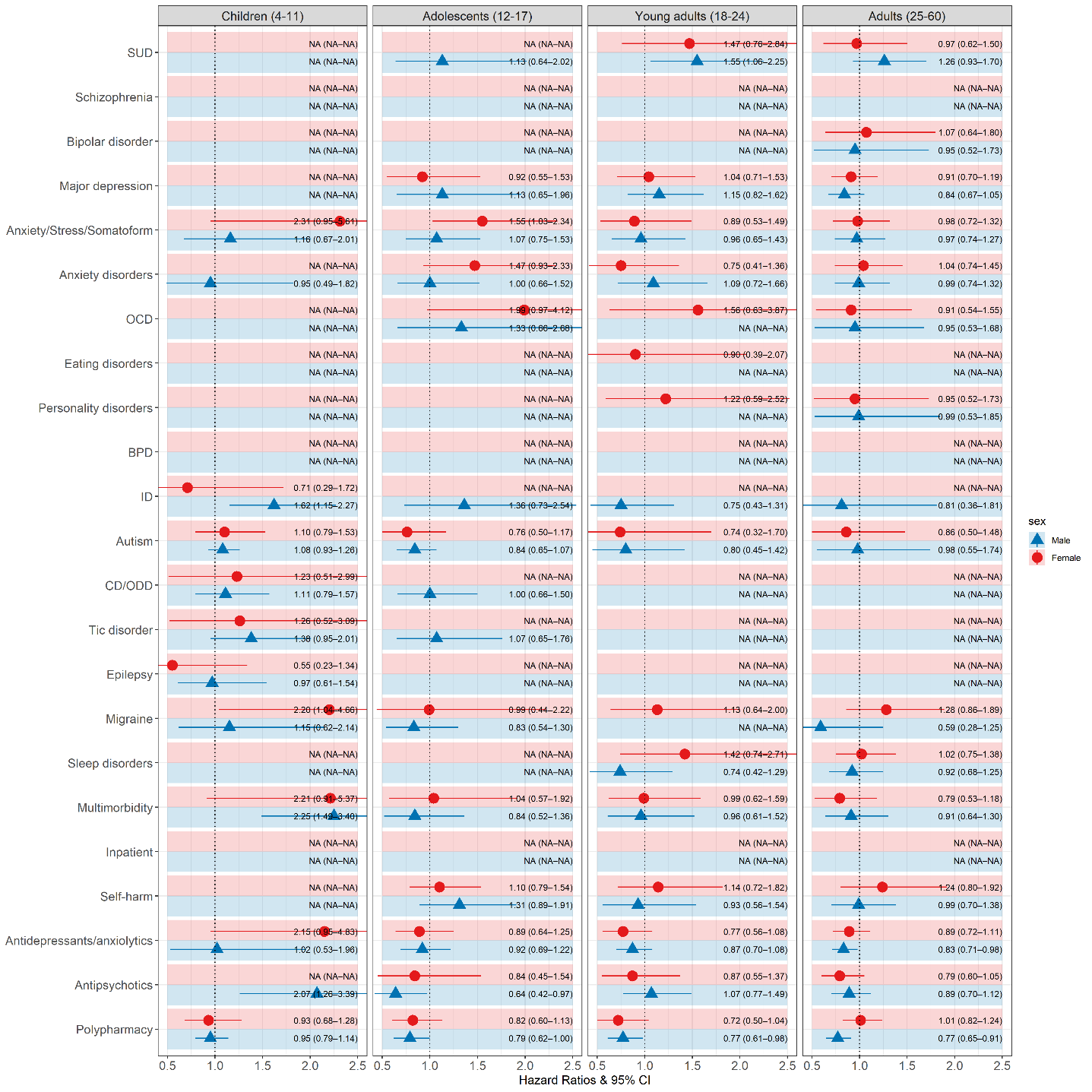


**Abbreviations:** SUD, substance use disorder; OCD, obsessive compulsive disorders; BPD, borderline personality disorder; ID, intellectual disability; CD/ODD, conduct disorder/oppositional defiant disorder; Inpatient, Psychiatric inpatient admission; Polypharmacy, psychotropic polypharmacy

## Figure S42. Sex-stratified associations of psychiatric and neurological indicators with ADHD medication discontinuation in the US


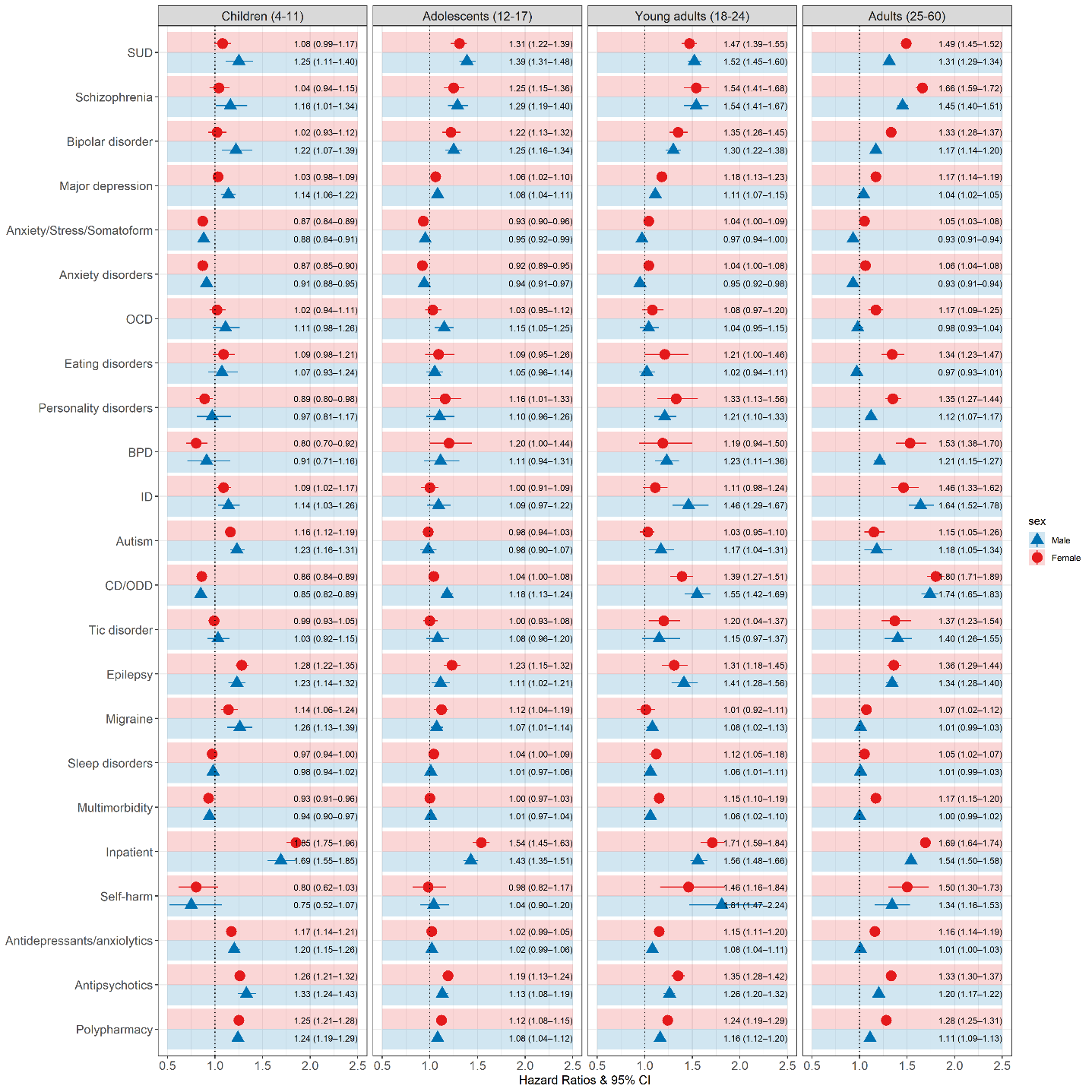


**Abbreviations:** SUD, substance use disorder; OCD, obsessive compulsive disorders; BPD, borderline personality disorder; ID, intellectual disability; CD/ODD, conduct disorder/oppositional defiant disorder; Inpatient, Psychiatric inpatient admission; Polypharmacy, psychotropic polypharmacy

## Figure S43. Meta-analytic associations of psychiatric and neurological indicators with ADHD medication discontinuation in individuals with a second ADHD medication dispensation within 180 days of initiation


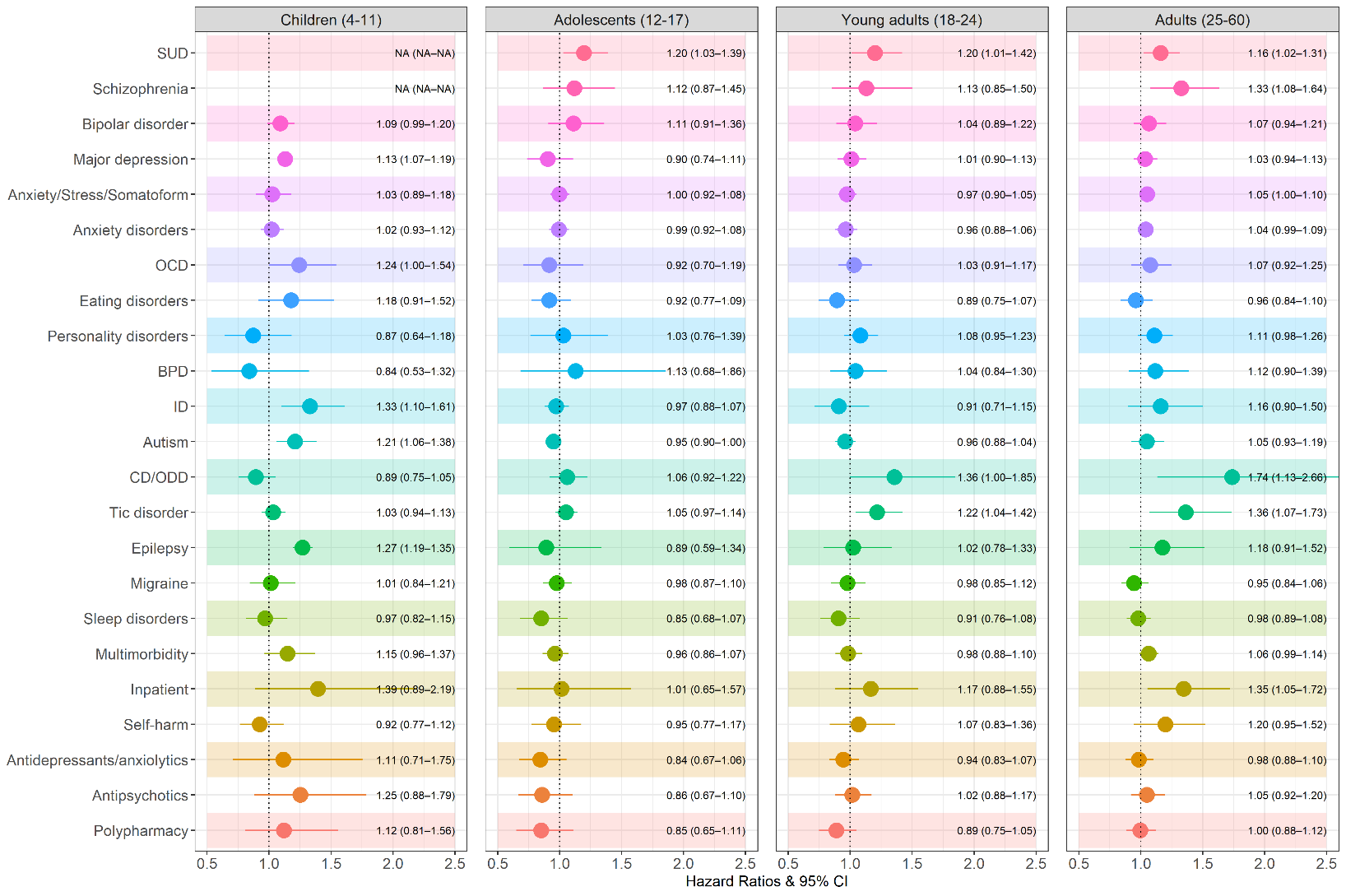


**Note:** Analyses restricted to individuals with a second ADHD medication dispensation within 180 days of their initial dispensation, with follow-up starting as the 2^nd^ dispensation. Abbreviations: SUD, substance use disorder; OCD, obsessive compulsive disorders; BPD, borderline personality disorder; ID, intellectual disability; CD/ODD, conduct disorder/oppositional defiant disorder; Inpatient, Psychiatric inpatient admission; Polypharmacy, psychotropic polypharmacy.

## Figure S44. Country specific associations of psychiatric and neurological indicators with ADHD medication discontinuation in individuals with a 2^nd^ dispensation within 180 days of initiation (Australia, Hong Kong, the Netherlands, Norway)


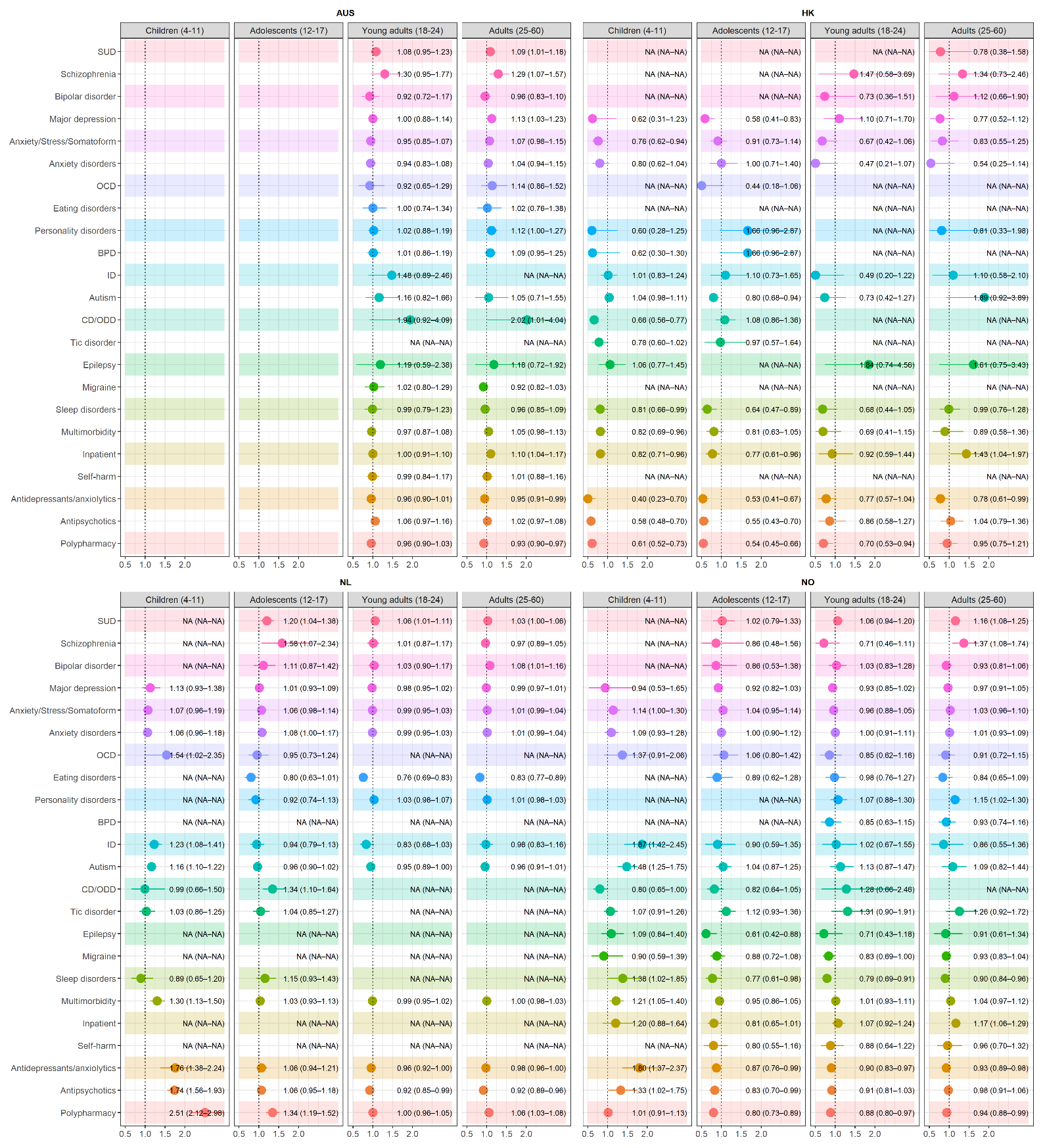


**Note:** Analyses restricted to individuals with a second ADHD medication dispensation within 180 days of their initial dispensation, with follow-up starting as the 2^nd^ dispensation. Abbreviations: SUD, substance use disorder; OCD, obsessive compulsive disorders; BPD, borderline personality disorder; ID, intellectual disability; CD/ODD, conduct disorder/oppositional defiant disorder; Inpatient, Psychiatric inpatient admission; Polypharmacy, psychotropic polypharmacy.

## Figure S45. Country specific associations of psychiatric and neurological indicators with ADHD medication discontinuation in individuals with a 2^nd^ dispensation within 180 days of initiation (Sweden, the United Kingdom, the United States)


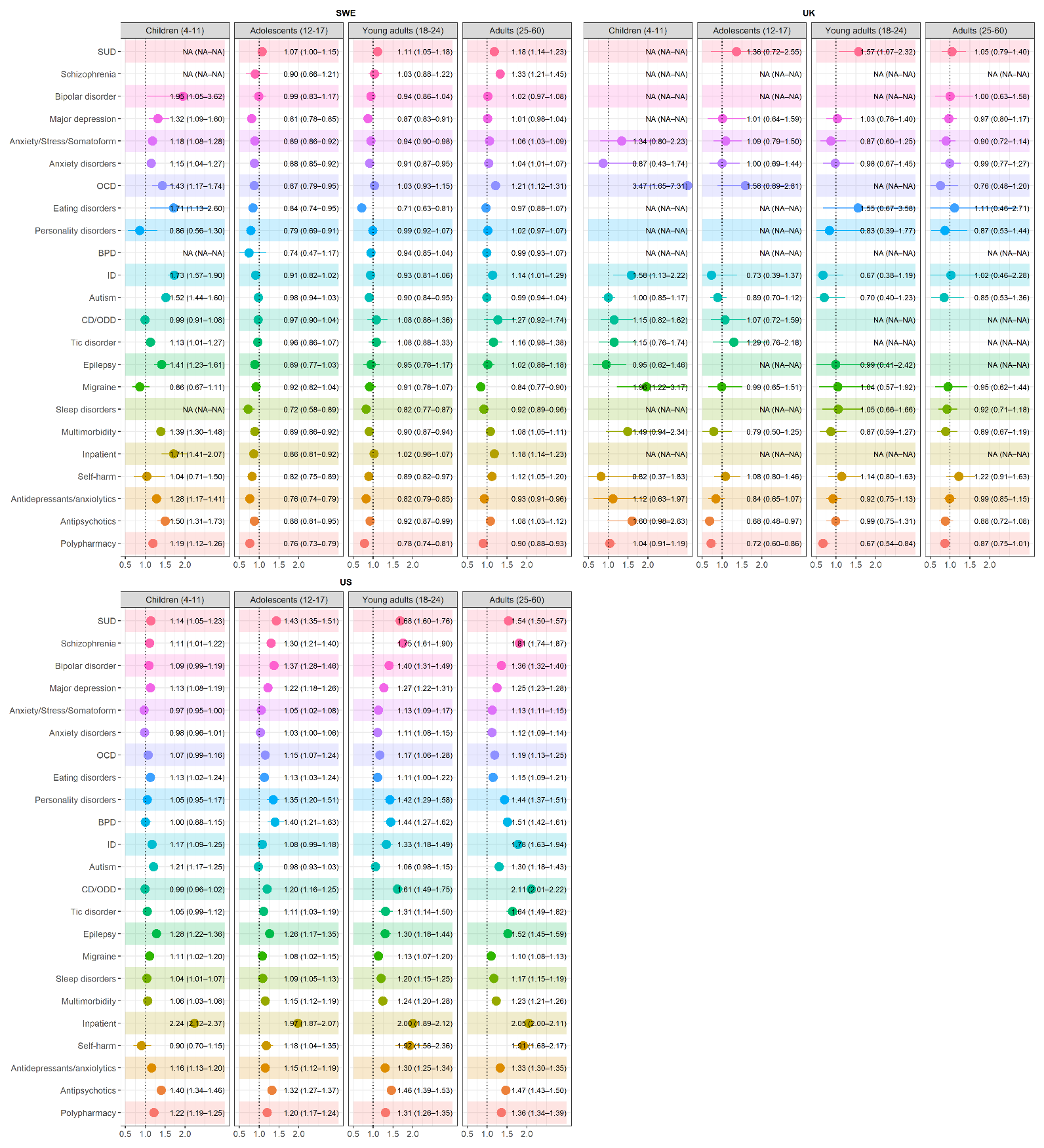


**Note:** Analyses restricted to individuals with a second ADHD medication dispensation within 180 days of their initial dispensation, with follow-up starting as the 2^nd^ dispensation. Abbreviations: SUD, substance use disorder; OCD, obsessive compulsive disorders; BPD, borderline personality disorder; ID, intellectual disability; CD/ODD, conduct disorder/oppositional defiant disorder; Inpatient, Psychiatric inpatient admission; Polypharmacy, psychotropic polypharmacy.

## Figure S46. Meta-analytic associations of psychiatric and neurological indicators defined as life-time with ADHD medication discontinuation


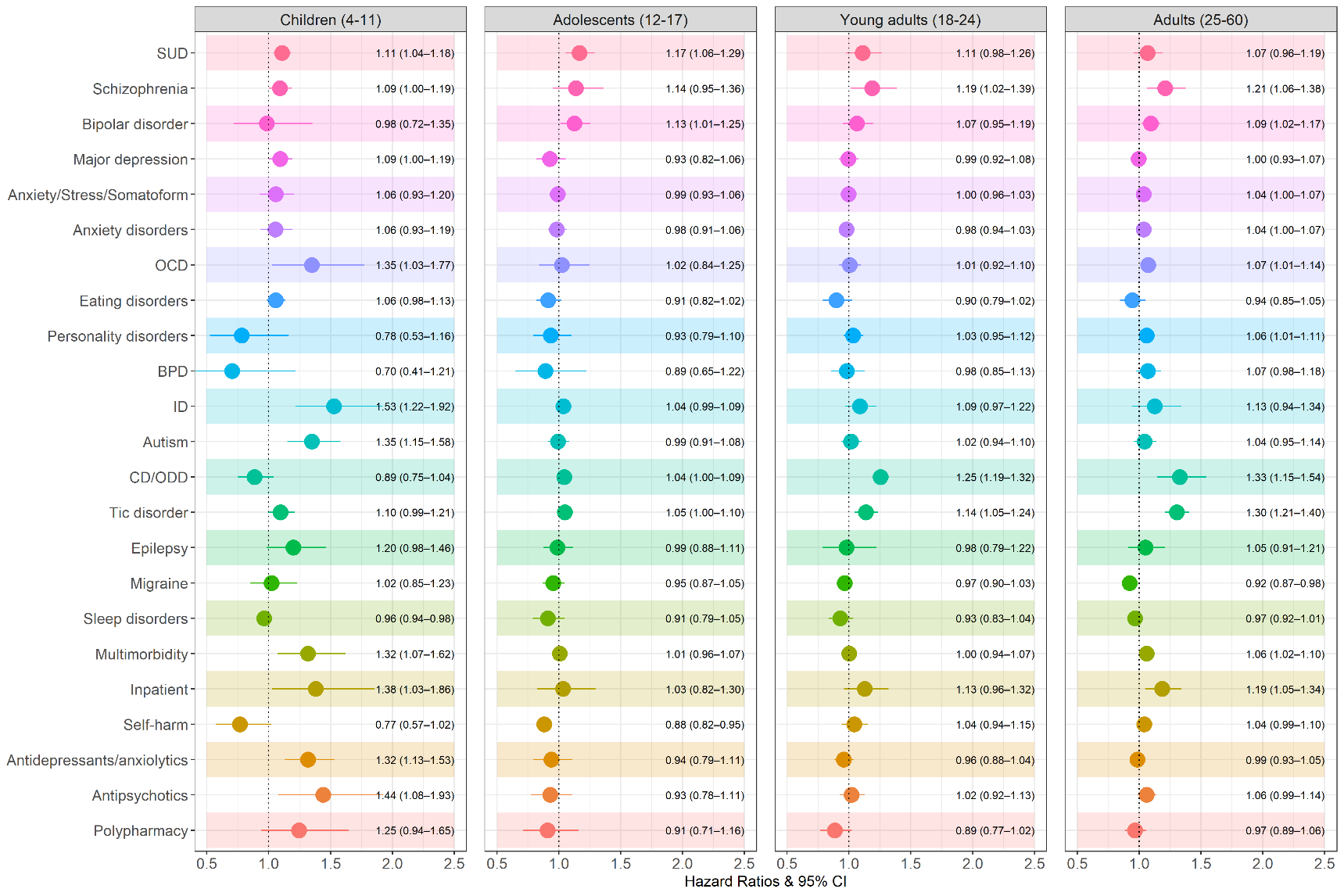


**Note**: Life-time indicators were defined using dispensations and ICD-10 coded diagnoses at any time preceding initiation, using data as far back as possible in each country/region. Abbreviations: SUD, substance use disorder; OCD, obsessive compulsive disorders; BPD, borderline personality disorder; ID, intellectual disability; CD/ODD, conduct disorder/oppositional defiant disorder; Inpatient, Psychiatric inpatient admission; Polypharmacy, psychotropic polypharmacy

## Figure S47. Country specific associations of psychiatric and neurological indicators defined as life-time with ADHD medication discontinuation (Australia, Hong Kong, the Netherlands, Norway)


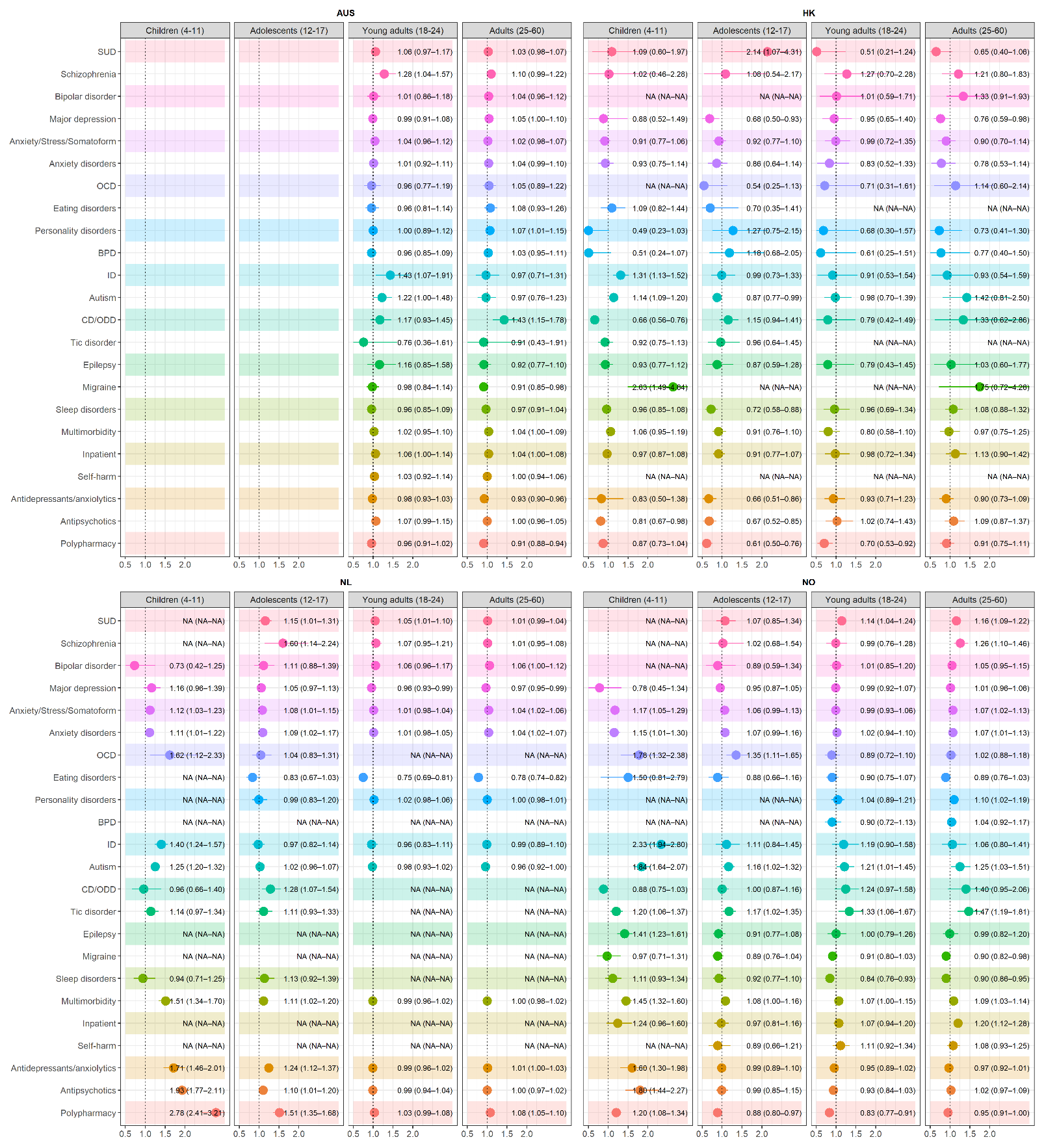


**Note**: Life-time indicators were defined using dispensations and ICD-10 coded diagnoses at any time preceding initiation, using data going as far back as possible in each country. Abbreviations: SUD, substance use disorder; OCD, obsessive compulsive disorders; BPD, borderline personality disorder; ID, intellectual disability; CD/ODD, conduct disorder/oppositional defiant disorder; Inpatient, Psychiatric inpatient admission; Polypharmacy, psychotropic polypharmacy.

## Figure S48. Country specific associations of psychiatric and neurological indicators defined as life-time with ADHD medication discontinuation (Sweden, the United Kingdom, the United States)


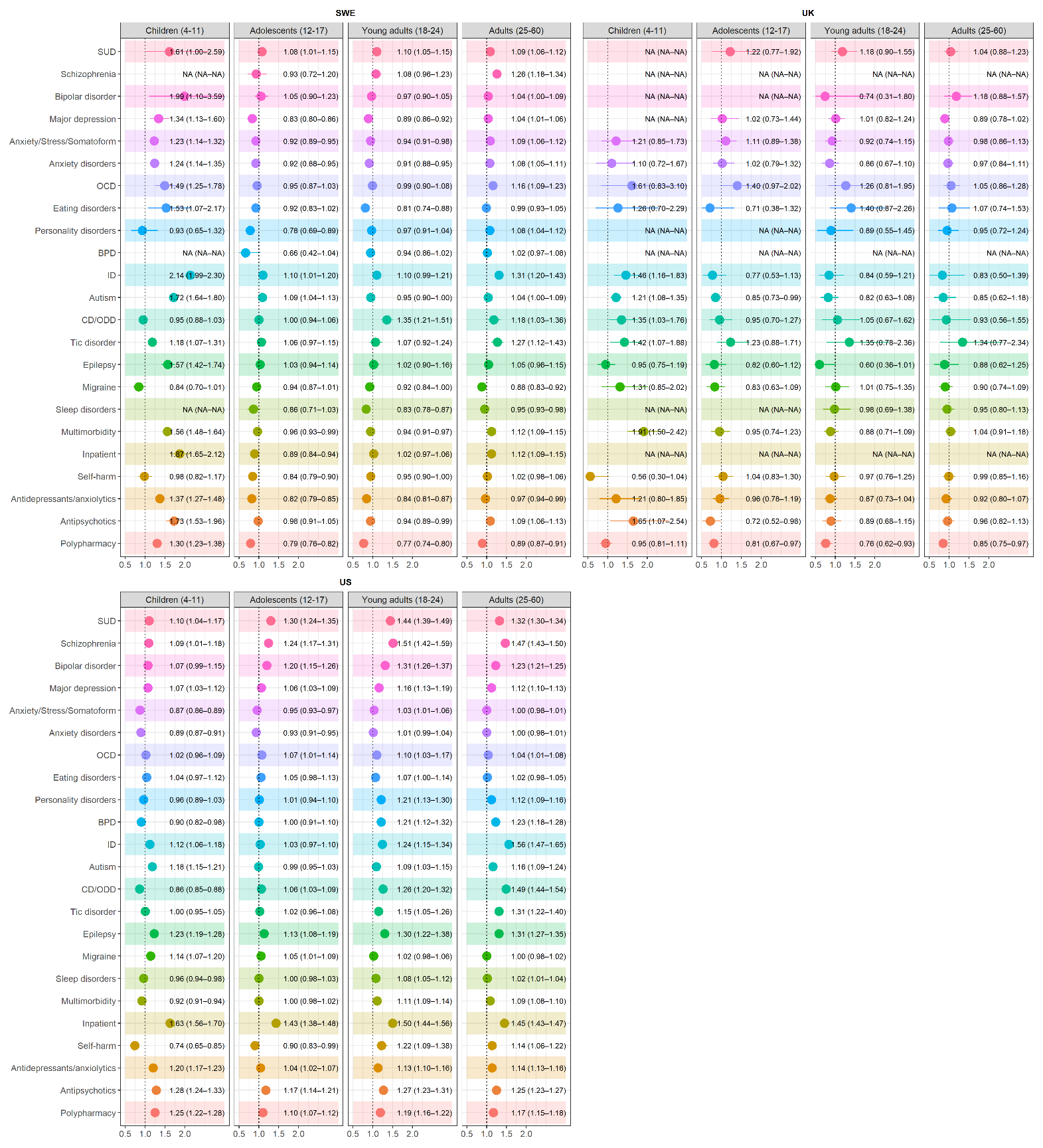


**Note**: Life-time indicators were defined using dispensations and ICD-10 coded diagnoses at any time preceding initiation, using data going as far back as possible in each country. Abbreviations: SUD, substance use disorder; OCD, obsessive compulsive disorders; BPD, borderline personality disorder; ID, intellectual disability; CD/ODD, conduct disorder/oppositional defiant disorder; Inpatient, Psychiatric inpatient admission; Polypharmacy, psychotropic polypharmacy.
